# Whole Genome Sequencing Highlights Genetic Changes Associated with Laboratory Domestication of *C. elegans*

**DOI:** 10.1371/journal.pone.0013922

**Published:** 2010-11-11

**Authors:** Katherine P. Weber, Subhajyoti De, Iwanka Kozarewa, Daniel J. Turner, M. Madan Babu, Mario de Bono

**Affiliations:** 1 Medical Research Council Laboratory of Molecular Biology, Cambridge, United Kingdom; 2 Wellcome Trust Sanger Institute, Hinxton, United Kingdom; Centre for Genomic Regulation, Spain

## Abstract

Defining the mutational landscape when individuals of a species grow separately and diverge over many generations can provide insights into trait evolution. A specific example of this involves studying changes associated with domestication where different lines of the same wild stock have been cultivated independently in different standard environments. Whole genome sequence comparison of such lines permits estimation of mutation rates, inference of genes' ancestral states and ancestry of existing strains, and correction of sequencing errors in genome databases. Here we study domestication of the *C. elegans* Bristol strain as a model, and report the genome sequence of LSJ1 (Bristol), a sibling of the standard *C. elegans* reference wild type N2 (Bristol). The LSJ1 and N2 lines were cultivated separately from shortly after the Bristol strain was isolated until methods to freeze *C. elegans* were developed. We find that during this time the two strains have accumulated 1208 genetic differences. We describe phenotypic variation between N2 and LSJ1 in the rate at which embryos develop, the rate of production of eggs, the maturity of eggs at laying, and feeding behavior, all the result of post-isolation changes. We infer the ancestral alleles in the original Bristol isolate and highlight 2038 likely sequencing errors in the original N2 reference genome sequence. Many of these changes modify genome annotation. Our study provides a starting point to further investigate genotype-phenotype association and offers insights into the process of selection as a result of laboratory domestication.

## Introduction

Selective breeding during domestication can lead to rapid and dramatic changes in phenotype [1]. Understanding domestication is important both because it can link phenotypic selection with genetic change, and because it has been central to human success [2], [3]. Laboratory strains of model organisms are unusual examples of domestication in which extensive inbreeding has maximized phenotypic uniformity and artificial selection has been used to improve laboratory handling [4]–[6]. Inbred lab strains often exhibit significant phenotypic differences from wild isolates of the same species. For example, olfactory responses of lab strains of *Drosophila* differ from those of recently caught wild strains [7]. Lab-adapted mouse strains show reduced exploratory behavior [8], agility and strength [9], and risk aversion [10] compared to wild caught mice. However, the consequences of laboratory domestication have not been systematically investigated in any animal.

**Figure 1:**
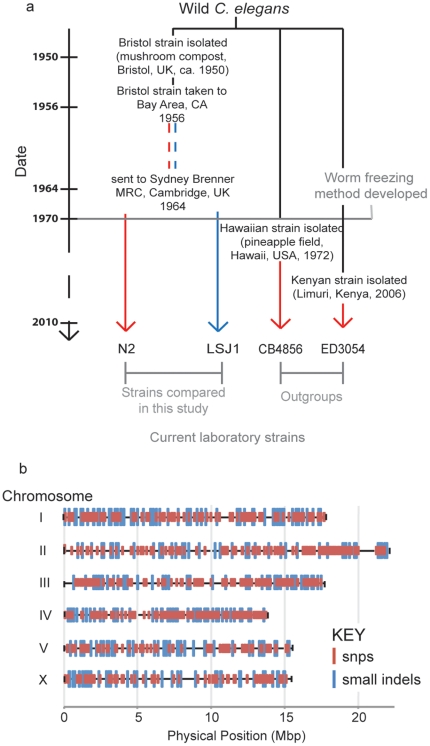
Laboratory strain cultivation history (a) and chromosomal distribution of N2/LSJ1 polymorphisms (b). (a) Red lines indicate that worms were maintained in solid culture, and blue lines in liquid culture. (b) SNPs are indicated by red hatches, and small insertions and deletions by blue hatches. Changes are distributed uniformly on chrmosomes I, II and X and slightly enriched on the arms of chromosomes III, IV and V (K-S test, P values adjusted using FDR).

With a few exceptions [11]–[16], laboratory studies of the nematode *C. elegans* have used the same wild type reference strain: N2 (Bristol). The progenitor of this strain was collected in Bristol, England, sometime before 1956 by L.N. Staniland [17], [18]. E. Dougherty took its descendants to California, where two stocks of the strain were maintained from 1956 by serial transfer, one in axenic liquid culture and the other on nutrient agar inoculated with *E. coli* [17]. In 1964, Dougherty sent Sydney Brenner at the MRC Laboratory of Molecular Biology one of these *C. elegans* sub-strains. Brenner used this stock to establish the laboratory reference *C. elegans* wild strain, which he called N2 (Bristol). At the MRC, N2 was maintained on agar plates with an *E. coli* food source until c. 1970, when the strain was frozen (Figure 1a).

The sibling stock of the Bristol isolate that was cultivated in axenic liquid culture appears to have also survived, and is called LSJ1(Berkeley) [16]. Here we refer to it as LSJ1(Bristol) to indicate its origins. Distinct growth conditions and inadvertent selective breeding are likely to have exposed the N2 and LSJ1 sub-cultures to different selective pressures. Additionally, both strains likely experienced genetic bottlenecks, with associated founder affects, prior to freezing. Comparing the genomes of N2 and LSJ1 Bristol strains would highlight genetic changes associated with lab domestication as well as errors in the *C. elegans* reference genome. The ancestral and derived alleles at each polymorphic locus could also be established by comparing the genomes of Bristol-derived strains and of other wild *C. elegans* isolates.

One striking difference between N2 and most other wild *C. elegans* isolates is in foraging [11]. N2 feeds alone, whereas most wild isolates feed in groups [11], [15]. Dimorphism in foraging is associated with two alleles of the neuropeptide receptor *npr-1* that differ at codon 215. Aggregating strains encode the ancestral *npr-1 215F* allele whereas N2 encodes *npr-1 215V* [11], [19]. Twelve other wild isolates that are genetically closely related to N2 also show solitary behavior and encode *npr-1 215V*, which would be consistent with recent occurrence of the polymorphism in wild populations. Surprisingly however, the LSJ1 strain feeds in groups and encodes the *npr-1 215F* allele, which would suggest that the *npr-1 215V* allele arose during laboratory domestication of N2 [13], [20]. These conflicting data suggest two hypotheses: that the 12 solitary wild isolates collected by several individuals over many years are all cases of switched strain identities or of strain cross-contamination, and are in fact N2 (Bristol)-derived; alternatively, that the LSJ1 strain is a wild strain distinct from Bristol. Whole genome sequencing of LSJ1 provides an opportunity to distinguish between these hypotheses.

Illumina sequencing has been successfully used in *C. elegans* both to sequence a wild isolate [21] and to identify lesions in mutants isolated in genetic screens [22]. Here we report the sequence of the LSJ1 (Bristol) strain and compare it to the reference N2 (Bristol) genome sequence. We confirm that LSJ1 and N2 are derived from the same Bristol isolate. We identify only 3246 predicted differences between the LSJ1 and N2 genome sequences. Of these changes, 2038 are likely errors in the original N2 sequence, since we find the LSJ1 allele in N2 strains; correcting them alters annotation of the *C. elegans* genome. The remaining 1208 polymorphisms arose in either the N2 or the LSJ1 lineages during lab domestication. By sequencing two recently isolated wild *C. elegans* strains, CB4856 (Hawaii) and ED3054 (Kenya), we infer the ancestral state of the original Bristol isolate. Alleles that arose during domestication include at least 88 changes that alter protein sequences. Side-by-side comparison of N2 and LSJ1 strains reveals significant phenotypic differences: N2 animals develop more quickly, lay eggs at a faster rate, lay eggs that have reached later developmental stages, and forage differently from LSJ1 animals. For one trait, aggregation/dispersal on food, our data suggest that standard laboratory *C. elegans* husbandry selects strongly for the allele found in the N2 domesticated strain. For another, the stage at which eggs are laid, we map N2/LSJ1 phenotypic variation to two regions on *C. elegans* chromosome V. Our work sets the scene for using *C. elegans* as a model for studying animal domestication. Additionally, the sequence differences we highlight between the N2 reference genome and LSJ1 provide a first pass filter to screen out false positives when using Next Generation Sequencing to identify induced mutations.

## Results

### Comparing the LSJ1(Bristol) genome with the N2(Bristol) reference sequence

To examine how the *C. elegans* Bristol wild isolate has evolved during laboratory cultivation we sequenced the LSJ1 strain of this isolate using Illumina technology. To minimize sequencing bias we omitted PCR amplification steps in making our genome libraries [23]. We targeted an average library insert size of 200 bp, which we sequenced at both ends. From four sequencing lanes, two each with 54 bp and 76 bp reads, we obtained an average of 79.94-fold coverage of the LSJ1 genome. The average per-lane yield of LSJ1 sequence was 1.45 Gb using 54 bp reads and 2.57 Gb using 76 bp reads, corresponding to 14.20× and 25.11× coverage of the *C. elegans* genome, respectively. Alignment to the N2 reference genome (release WS203) [24] showed that over 99% of positions in the genome were covered by this sequence. The remaining positions represented repeat DNA or regions of low-complexity sequence. Thus a single Illumina Genome Analyzer IIx flow cell lane was sufficient to obtain >20 fold coverage of the *C. elegans* genome when using 76 bp reads. Raw sequence data for LSJ1, as well as for the wild strains ED3054 and CB4856, which were used as outgroups in this study, is available in the NCBI Short Read Archive (Accession number SRA024308).

The N2 and LSJ1 strains were genetically very similar: we identified 1425 potential single nucleotide polymorphisms (SNPs) between them, and 1821 small (1–3 bp) insertions and deletions (indels) (Tables S1 and S2). Small indels can be difficult to identify using Illumina data, so we sequenced a subset of 67 indels, with a variety of alignment quality scores, using traditional Sanger sequencing. We found that Sanger sequencing confirmed almost all (38/40) indels that were called unambiguously by the BWA alignment program, and those called ambiguously were almost never (2/27) confirmed. We therefore filtered our small indel data for only unambiguously called indels. We also found 31 larger deletions (between 5 and 297bp) and 10 larger insertions (between 5 and 33bp) (Table S3).

The changes we identified were expected to include both genuine variation between the N2 and LSJ1 genomes and sequencing errors in the N2 reference sequence or in our Illumina data. Errors would be flagged as N2/LSJ1 polymorphisms in our comparison. To examine how frequently this occurred, we verified a small subset of the N2/LSJ1 polymorphisms by traditional Sanger sequencing. We chose 12 SNPs and 12 indels and sequenced them in both the LSJ1 strain and in our lab's N2 (Bristol) stock. The N2 stock we used was one of the earliest N2 samples frozen at the MRC. All LSJ1 sequences confirmed the data obtained by Illumina sequencing. Interestingly, however, for many of the polymorphisms (9 of 12 SNPs and 10 of 12 indels) the N2 strain in our lab had the LSJ1 and not the N2 allelic variant. This suggested that many apparent polymorphisms between the LSJ1 and N2 sequences represent errors in the reference sequence. Alternatively, the strains of N2 sequenced by the genome consortium had accumulated many more mutations than our N2 stock.

### Illumina sequence data of LSJ1 and different N2 stocks permits the N2 reference sequence to be updated

Since many putative N2/LSJ1 polymorphisms in our small test sample validated as LSJ1 alleles in our N2 strain, we extended our study across the genome using new N2 sequencing data. The original N2 reference sequence was obtained from DNA cloned in cosmid, phage and YAC libraries [24], but stocks of the N2 strains used to generate these libraries are not available. We therefore examined new Illumina sequence data from six other N2-derived strains obtained from 3 labs (we thank S. Nurrish, B. Olofsson and W. Schafer for the sequence). Specifically, we found that for 548 of the 1425 predicted N2/LSJ1 SNPs, at least 4 of the 6 N2 strains encoded the LSJ1 allele. Similarly, 1490 of 1821 indels occurred as LSJ1 alleles in at least 4 of the 6 N2 strains (Table S1). Note that because we do not have the original sequencing traces for the six N2 Illumina genome sequences, we cannot ascertain whether N2 strains that do not show the LSJ1 sequence at the positions shown in Table S1 actually have the N2 allele or have poor sequencing coverage at that position. Regardless, the bulk of these unconfirmed N2/LSJ1 polymorphisms are likely to be errors in the *C. elegans* reference sequence. This is not surprising: the *C. elegans* sequencing consortium predicted an error rate of less than 1 in 10,000 bp. Our data suggest an actual error rate in the region of 1 in 50,000 bp. The alternative hypothesis, that the N2 strains sequenced by the consortium had diverged substantially from currently used N2 strains is less likely for the bulk of the changes. Mutation accumulation studies suggest an average mutation rate of 2.7×10^−9^ per base pair per generation for the *C. elegans* genome [25]. This would predict accumulation of only 27 SNPs across the N2 genome over 100 generations of continuous lab cultivation. Fewer generations are likely to separate the N2 strains sequenced by the consortium and in this study.

We next examined how many of the sequencing errors altered gene predictions by identifying polymorphisms mapping to exons. We found 45 SNPs that lead to non-synonymous codon substitutions and 159 exonic indels (Table S4). The indels may have a range of effects on coding predictions, including frameshifts and exon skipping. Of the 159 exonic indels, 14 occurred in genes currently annotated in WormBase as pseudogenes, but that in reality, based on our data, may be functional (Table 1).

**Table 1:** Small indels found in genes currently annotated as pseudogenes that are putative errors in the N2 reference genome.

Chr	Variation	Gene Name	Gene Function	LSJ1_allele	Ancestral Status
I	8161379	T28B8.4	unknown	+G/+G	LSJ1
I	10315724	Y106G6G.5	unknown	+A/+A	LSJ1
II	6818451	F31E8.6	homology to DNA topoisomerase II	+G/+G	LSJ1
II	7825758	*srw-62*	serpentine receptor, class W	+C/+C	LSJ1
II	9527712	*sra-15*	serpentine receptor, class A	+C/+C	LSJ1
III	4684391	*clec-155*	c-type lectin	−g/−g	LSJ1
IV	9187483	C46C2.4	unknown	+G/+G	LSJ1
IV	9524145	C33A12.20	unknown	+G/+G	LSJ1
V	3556386	*grd-30*	ground-like related	+C/+C	LSJ1
V	10230413	C12D8.2	Major Sperm Protein (MSP) family	+G/+G	LSJ1
V	15440054	T26H5.6	unknown	+C/+C	LSJ1
V	19485465	Y43F8B.17	unknown	+G/+G	LSJ1
X	3293917	*hsp-2*	heat shock protein	−c/−c	LSJ1
X	7310131	*his-40*	histone-like	+C/+C	N2

### The number and distribution of genetic changes between N2 and LSJ1 is consistent with their expected degree of separation during domestication

Once we eliminated presumed N2 reference sequence errors, we identified 1208 differences between N2 and LSJ1, comprising 877 SNPs and 331 indels (Table S2). These polymorphisms were distributed uniformly among the six *C. elegans* chromosomes, with a slight enrichment on chromosome III (Figure 1b). The density of polymorphisms across each chromosome was also fairly uniform – chromosomes III, IV and V showed slight enrichment for polymorphisms on the chromosome arms (K-S test, p values corrected using FDR). This pattern recapitulates that observed in mutation accumulation studies [25], as would be expected for two strains that diverged in the laboratory. By contrast, polymorphisms found between N2 and other wild isolates show blocks of highly polymorphic regions interspersed with regions of low variation, and substantial enrichment of polymorphisms at the chromosome arms [26].

By comparing our data to genome-wide mutation accumulation studies in N2 (Bristol) we could estimate how many generations separate the N2 and LSJ1 strains. Denver et al estimate a substitution mutation rate m of 2.7×10^−9^ per base pair per generation for the *C. elegans* N2 genome [25]. Given this value, the 877 SNPs we identified between N2(Bristol) and LSJ1(Bristol) suggest the two strains were separated 1620 generations ago. Assuming a 4-day generation time, this translates to about 18 years. The number of generations that LSJ1 and N2 were bred separately is difficult to estimate, but an 18-year separation is consistent with the available information about the histories of these strains.

### Inferring the ancestral genotype of the Bristol strain

To infer the ancestral allele present in the original Bristol wild isolate, we used Illumina technology to determine the genome sequence of two other wild *C. elegans* isolates: CB4856 (Hawaii) and ED3054 (Kenya) (variation identified in these two strains, along with that found in LSJ1, will be available in Wormbase). These strains provided out-groups for the two Bristol sub-strains: we reasoned that whichever allele (N2 or LSJ1) was present in both non-Bristol wild strains was likely to be ancestral in Bristol. For 99% of polymorphisms, CB4856 and ED3054 gave concurring predictions (Table S2). In 12 cases (0.92%), we found the N2 allele in one strain and the LSJ1 allele in the other; we annotated such polymorphisms as having an unknown ancestral state. In total, 719 confirmed changes (59.8%) were found to have the N2 allele and 472 to have the LSJ1 allele (39.3%) as the ancestral state.

The mutational biases observed in mutation accumulation studies are consistent with base oxidation being a pre-eminent cause of DNA damage [25]. The N2 and LSJ1 strains were cultivated under different conditions: N2 on agar in 21% O_2_, and LSJ1 in axenic liquid culture that is typically hypoxic. We therefore examined if mutational bias in each lineage differed. As observed in the mutation accumulation lines studied by Denver et al. we saw enrichment of G∶C A∶T and G∶C T∶A mutations (the type of change expected from oxidative damage) (Figure S2), but we did not see a decrease in the frequency of these mutations in the LSJ1 lineage.

Mutation accumulation lines exhibited an average Ts/Tv (transitions/transversions) ratio of 0.45, with individual line values ranging from 0.19 to 0.79, which is lower than the ratios of 1.2 to 3.0 observed in wild *C. elegans* strains [25]–[29]. We observe a Ts/Tv rate of 0.74 overall, and 0.67 and 0.84 for ancestral-N2 and ancestral-LSJ1 changes, respectively (Figure S2). These data support the hypothesis that transversions are more likely to be selectively purged in the wild than in the laboratory [25].

### Trait variation between N2 and LSJ1 after domestication

**Figure 2:**
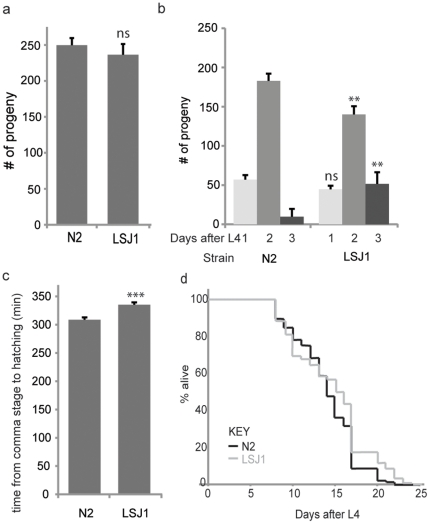
N2 and LSJ1 show life trait differences. (a and b) Although N2 and LSJ1 have similar brood sizes (a), LSJ1 lays eggs over a longer period (b) (n = 11 for both strains). (c) LSJ1 embryos (n = 64) develop slower than N2 (n = 78) but (d) show no difference in lifespan N2 (n = 76) and LSJ1 (n = 85) (K-S test). Asterisks correspond to significances of differences from N2 under identical conditions; ** indicates P<0.001, *** indicates P<0.0001; error bars indicate s.e.m.

Domestication is often associated with accelerated growth and reproduction. Standard lab husbandry of *C. elegans* N2 may also have selected for animals that grow faster and reproduce earlier than their wild progenitors. This is because animals used to set up fresh cultures are typically picked from among the first batch of progeny, which reach adulthood before growing animals exhaust food. To test this hypothesis we compared the egg-laying profiles of N2 and LSJ1 animals. The brood sizes of the two strains were not statistically different (Figure 2a). However whereas N2 animals that were picked as late L4 larvae laid 96.1% of their brood in two days, identically staged LSJ1 animals laid only 78.2% of their eggs during that time, and were still producing many eggs on the third day after L4 (Figure 2b). Thus N2 animals produce the same number of eggs as LSJ1 but in a shorter time period, as predicted.

As a measure of developmental rate we monitored the duration of embryonic development. We dissected N2 and LSJ1 gravid hermaphrodites and transferred early embryos from both strains to M9 buffer. Using time-lapse microscopy we recorded images of embryos every 5 minutes for 10 hours. We then measured the time it took for embryos to progress from the comma stage (the beginning of gastrulation) to hatching, and found that N2 embryos took an average of 309 minutes, while LSJ1 embryos took 325 minutes (Figure 2c). Thus N2 embryos also develop faster than their LSJ1 counterparts.

Since *C. elegans* that grow more slowly often live longer, we also examined the lifespan of LSJ1. However we found that LSJ1 did not live significantly longer than N2 (Figure 2d).

**Figure 3:**
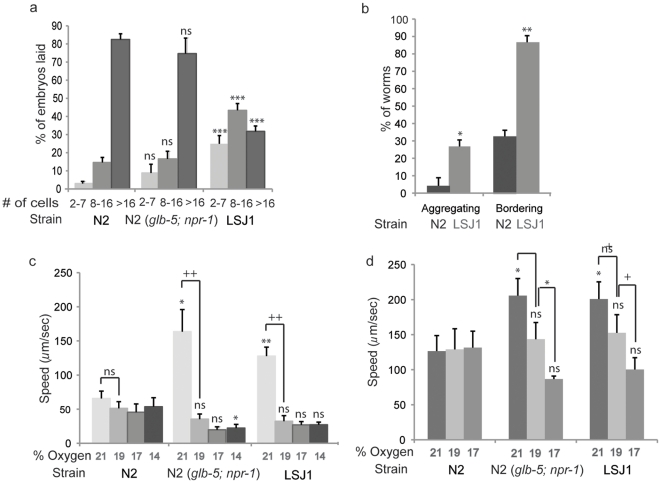
N2 and LSJ1 show behavioral variation. (a) Variation in egg laying (n = 25 for both strains). Egg laying results were fitted to a binomial model and assessed with an ANOVA (2 degrees of freedom, F = 71.05, P = 2.2×10^−15^). Significances of differences in egg laying were assessed using a Mann-Whitney test with a posthoc Bonferroni correction. (b) Variation in aggregation and bordering behaviors (n = 116 for N2, n = 121 for LSJ1). (c) Variation in oxygen responses on food (n = 30 for both strains). (d) Variation in oxygen response off food (n = 40 for LSJ1, n = 30 for N2). In all panels, asterisks indicate significances of differences from N2 under identical conditions, while plusses indicate significance of speed alteration within strains in response to oxygen changes; ns = not significant, */+ indicates p<0.01, **/++ indicates p<0.001, and *** indicates p<0.0001; error bars indicate s.e.m.

We next examined the stage at which LSJ1 lays its eggs by letting animals lay eggs for one hour and then examining their developmental stage. We classified embryos into 3 groups: the 8-cell stage or younger, between the 8- and 16-cell stages, and older than the 16-cell stage. We found about 50% of LSJ1 eggs were laid when embryos were younger than the 16 cell stage, whereas less than 10% of N2 eggs were laid when embryos were at this early stage (Figure 3a). Whether laying early eggs reflects changes in egg-production, egg-laying frequency, or both, is unclear.

Using standard genetic mapping methods (see Materials and Methods) we found that the LSJ1 early egg laying phenotype was linked to LSJ1 DNA on chromosomes II and V (Figure S3a). Overall, the phenotype was dominant in an LSJ1 x N2 cross, (Figure S3b). However genetic dissection using introgressed lines revealed both a recessive locus or loci on the second chromosome that had a weak but not significant early egg laying phenotype on its own (Figure S3c), and a dominant locus or loci on chromosome V that had a strong early egg laying phenotype (Figure S3c). When we picked recombinants to map the locus on V we found it did not map to a single interval, but could be reconstituted in the transheterozygous F1 progeny of recombinants that had LSJ1 DNA on opposite arms of chromosome V, and N2 DNA at other genomic locations (Figure S3c). These data suggest that LSJ1 alleles of one or more genes on each arm of chromosome V act together to generate the early egg laying phenotype. Our genome sequencing studies have identified a small number of genes in these two intervals that have coding region changes between N2 and LSJ1. These include, on the left arm: F53E10.1 (encoding an FeS cluster protein), T22F3.2 (a ubiquitin carboxyl terminal transferase), K09D9.12 (function unknown), C18G1.8 (glycosyl transferase), and on the right arm: *str-200* (seven transmembrane receptor) and M162.7 (currently annotated as a pseudogene). These genes are good candidates for the loci responsible for the different early/late egg-laying phenotypes of N2 and LSJ1.

Finally, we examined aggregation, bordering, and oxygen responses of N2 and LSJ1 animals. As expected given their genotype at the *npr-1* locus [13], LSJ1 animals aggregated and bordered strongly compared to N2. Nearly 90% of LSJ1 animals were found at the border of a 2-day lawn of OP50 after 1 hour, while only 32% of N2 animals were at the border (Figure 3b). Over a quarter of LSJ1 worms were found in groups of two or more worms, while less than 5% of N2 animals formed aggregates (Figure 3b).

*C. elegans* aggregation partly reflects avoidance of high ambient O_2_ [30] [31] [32]. We therefore subjected N2 and LSJ1 animals to changes in ambient O_2_ and measured their locomotory responses. Feeding N2 animals did not significantly alter their speed when exposed to O_2_ decreases and moved relatively slowly (Figure 3c). In contrast, LSJ1 animals exhibited high locomotory activity at 21% O_2_ and progressively reduced speed at 19% and 17% oxygen (Figure 3c). These results recapitulate those observed in an N2 strain that bears the LSJ1 alleles of *glb-5* and *npr-1* [15], (Figure 3c). LSJ1 also behaved similarly to a *glb-5; npr-1* (N2 background) strain when exposed to gradual changes in ambient O_2_ in the absence of food (Figure 3d). Under these conditions, LSJ1 moved faster than N2 at 21% O_2_ and reduced its rate of locomotion as O_2_ fell to 19 and 17%, although not as dramatically as when food was present.

### Standard *C. elegans* husbandry strongly selects for solitary feeding

**Figure 4:**
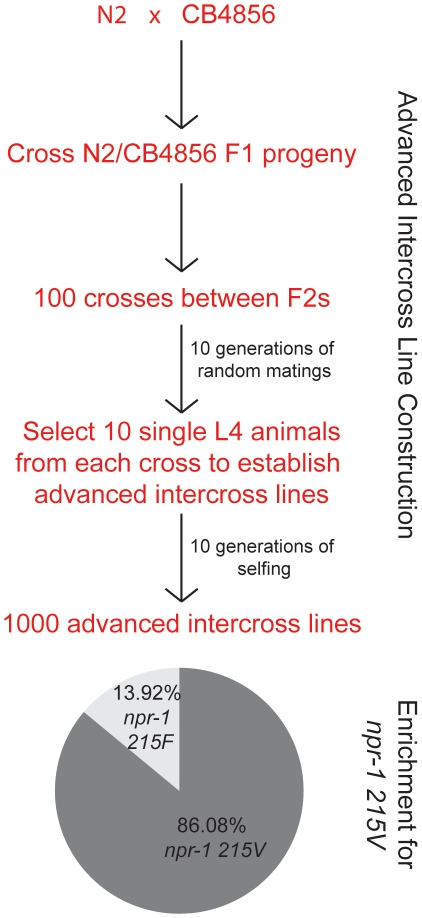
Laboratory cultivation selects for solitary feeding. The flowchart describes construction of advanced intercross lines, which yielded many more strains carrying the 215V allele of *npr-1* than the *npr-1 215F* variant.

The evolution of solitary feeding in domesticated N2 animals may represent a chance occurrence, or it could reflect strong selective pressure for this behavior associated with standard lab husbandry. To distinguish between these possibilities, we examined inheritance of aggregation behavior and of the two alleles of *npr-1* in 1000 advanced intercross lines made between N2 and the aggregating Hawaiian strain CB4856 (Figure 4). After 10 generations of random inter-line matings between 100 different crosses, each mating plate was used to pick 10 L4 hermaphrodites that were allowed to self-fertilize on individual plates, creating 1000 lines. Each line was then propagated for a further 9 generations by blindly picking single hermaphrodites to a fresh plate (Figure 4). At the end of the experiment we genotyped DNA from each line for *npr-1*. Out of 978 lines that remained at the end of the experiment, 841 had the N2 allele of *npr-1* (86.1%) whereas 136 animals had the CB4856 allele (13.9%) (Figure 4). By contrast, the distribution of N2 and CB4856 alleles on the same chromosome as *npr-1* but 20 map units away was 54% CB4856 and 46% N2, and on the autosomes the distribution of CB4856 and N2 DNA was approximately equal. Our data point towards strong selection for N2 DNA close to *npr-1* and relaxed selection further away from this locus. These data are consistent with standard lab husbandry of *C. elegans* strongly selecting for the *npr-1 215V* allele and solitary feeding behavior.

## Discussion

*C. elegans* is used widely as a model to probe questions in biology. Most labs use N2 (Bristol) as the reference *C. elegans* wild strain. This strain has been maintained in the lab for more than 50 years. Here we determine the genome sequence of LSJ1(Bristol), a strain derived from the same wild isolate as N2 but cultivated separately from it at least since 1964. By comparing the reference N2 genome sequence to the genome sequences of LSJ1 and of several N2 lab strains we identify genetic changes associated with domestication of Bristol and errors in the reference sequence.

We identify 2038 changes between LSJ1 and the N2 reference sequence that are likely to be sequencing errors, although a few could be variation between N2 isolates. These changes give an error rate of only 1 in 50,000 bases in the reference sequence. 206 of the errors we identify localize to exons. Of these, 152 are 1- or 2-bp insertions or deletions: 138 may introduce frameshifts that substantially change annotated gene structure or transform predicted protein-coding genes to predicted pseudogenes. The remaining 14 small indels map to pseudogenes currently predicted to produce no protein product. These include genes with homology to a topoisomerase, a histone-like protein, and a heat shock protein (Table 1), and correcting these errors may mean that these genes are predicted to be functional. The remaining 54 exonic errors include single nucleotide polymorphisms (SNPs) or 3bp indels that alter, introduce, or remove a single amino acid and are therefore less likely to drastically change gene predictions than frameshift-inducing indels. However, 5 such SNPs introduce terminator codons in 4 genes (ZK507.1, E02C12.10, *sto-5* and *gcy-11*, see Table S4), shortening the predicted protein length. These changes will appear in the *C. elegans* online database WormBase. As genome sequencing becomes increasingly affordable, many genomes are being re-sequenced. Our study emphasizes the importance of refining genome annotation as revised sequence information becomes available.

We identified 1208 SNPs and small indels, and 41 larger indels, between the N2 and LSJ1 strains. Of these, 72 are expected to cause changes at the protein level (53 SNPs and 19 indels; Table S5). We also detected 41 larger indels, 7 of which are expected to affect protein coding (Table S3). The sample size of coding changes between N2 and LSJ1 is small. Nevertheless, there seems to be a slight enrichment for cell cycle and metabolic/growth genes (Table S5). Genes that regulate growth rate and nutrition have previously been found to be under selection in domesticated animals [33] [34]. This may also hold true for domesticated laboratory strains where there is selection for the quickest-growing animals, and increased nutrient availability as compared to the wild.

N2 and LSJ1 exhibit phenotypic differences. N2 animals grow significantly faster than LSJ1 animals and lay their brood more quickly. In addition, LSJ1 animals lay eggs at earlier stages of development. Whether these phenotypes result from polymorphisms in the same genes or segregate independently is unclear. However, because the total number of changes between the strains is so small, it is likely that each phenotypic difference corresponds to a small number of genetic alterations. Genotyping of domestic dogs indicates that the wide-ranging phenotypic diversity between dogs is also generated by just a few genetic changes [3], and this may be a general feature of traits selected under domestication. This is true in the case of the *C. elegans* gene *npr-1*, where a change in a single gene leads to dramatic behavioral variation, and for the early egg laying phenotype that we describe, which seems to be controlled by just two genes on chromosome V, and possibly by one or more genes on chromosome II. Such simple genetic structure of phenotypic variation selected in the laboratory is in contrast to the genetic structure of natural phenotypic variation. Natural variation is usually mediated by small-effect changes in many genes that interact in complex ways [35], [36].

We previously showed that the *215F* allele of *npr-1* is ancestral in *C. elegans*, and that *npr-1 215V* arose recently [19]. The very similar genomes of N2 and LSJ1, and the fact that these two strains encode *npr-1 215V* and *npr-1 215F* respectively [20] [13] provide strong evidence that the solitary N2 strain arose from an ancestral social strain during *C. elegans* laboratory cultivation. A corollary of this is that the 12 solitary isolates collected between 1971 and 2003 are inadvertent re-isolates of N2 or N2-derived strains. Our advanced intercross breeding data suggest strong selection against *npr-1 215F* or for *npr-1 215V* under standard lab husbandry, but we have not identified which of the constellation of polymorphic behaviors associated with variation in *npr-1* is most important for selection. *npr-1 215V* animals fail to burrow into agar plates, and one possibility is that these worms are preferentially selected during strain transfers; another is that solitary worms are more easily selected than those in groups when transferring worms in solid culture.

The 1249 genetic differences between the N2 and LSJ1 strains are distributed evenly across all 6 chromosomes (Figure 1a). These polymorphisms provide a useful resource for the *C. elegans* community as markers for genetic mapping experiments. Most mapping experiments currently use the Hawaiian strain CB4856 [37]. However, mapping in the Hawaiian background can be problematic for some phenotypes due to phenotypic variation between the CB4856 and N2 strains. The limited number of polymorphisms between N2 and LSJ1 make this less likely to be a problem. The resolution from N2 x LSJ1 mapping experiments is limited due to the low number of polymorphisms between the two strains (about 1 polymorphism every 85 000 bp). However this may not be a problem if a rough mapping approach is combined with whole genome sequencing [22]. As sequencing costs fall, whole genome sequencing will become increasingly popular as a method to molecularly characterize *C. elegans* mutants. Our work facilitates this approach: the sequence differences we highlight between the N2 reference genome and LSJ1 provide a first pass filter to screen out false positives when seeking to identify induced mutations.

## Materials and Methods

### Strains

Strains were maintained at 22°C using standard methods unless otherwise indicated [38]. Strains used in this study are: AX1796 *glb-5 (Hawaiian)* V; *npr-1(g320)* X; CB2030 *unc-62(e644) dpy-11(e224)* V; CB2065 *dpy-11(e644) unc-76(e911)* V; CB1870 *rol-1(e91) unc-4(e26)* II.

### Unamplified genomic library preparation

Adapter sequences used were:

A_adapter_t AATGATACGGCGACCACCGAGATCTACACTCTTTCCCTACACGACGCTCTTCCGATC*

A_adapter_b GATCGGAAGAGCGGTTCAGCAGGAATGCCGAGACCGATCTCGTATGCCGTCTTCTGCTTG

T, * indicates phosphorothioate; both adapters were HPLC purified.

Briefly, 40 µmoles of adapters were phosphorylated at their 5′ end by 1 U/µl T4 polynucleotide kinase (New England Biolabs) for 30 minutes at 37°C. The kinase was denatured at 94°C for 2 minutes, and the adapters annealed by cooling to 20°C at a rate of 0.1°C every 2 seconds. The adapters were divided into single-use aliquots and stored at -20°C.

Genomic DNA was prepared using a DNEasy Blood and Tissue kit (Qiagen). Approximately 5 µg *C. elegans* genomic DNA (quantified by NanoDrop) was fragmented to an average size of 200 bp using Covaris Adaptive Focused Acoustics technology with the settings: 20% Duty Cycle; Intensity 5; 200 Cycles per burst over the course of 3 minutes. After end repair and A-tailing following the standard Illumina protocols, the ligation reactions were set up in a total volume of 50 µl containing 10 µl template DNA, 20∶1 molar access of adapters, 1x Illumina DNA ligation buffer and 5 µl Illumina DNA ligase (2,000 U/µl). The reactions were incubated for 15 minutes at 20°C. Ligated samples were run on a 2% agarose gel and DNA fragments of the desired size extracted using a Gel extraction kit (Qiagen).

### Library quantification and sequencing

Libraries were quantified by qPCR, using three dilutions of a standard library (a similar library of known concentration) as a control [39]. Libraries were sequenced on an Illumina GAII Analyzer following the manufacturer's standard protocols.

### Genome assembly

Fastq files generated from the Illumina pipeline were converted to standard fastq format and filtered to remove exactly duplicated reads. The unique reads were aligned to the *C. elegans* reference genome (release WS203) using the BWA alignment program (Burrows-Wheeler Algorithm, [40], http://bio-bwa.sourceforge.net/). SAM alignment files were further processed to create pileup files using the SAMtools package (Sequence Alignment/Map format tools, [40], http://samtools.sourceforge.net/). Files containing N2/LSJ1 variation were generated from the Pileup format alignment files. Single Nucleotide Polymorphisms (SNPs) with variation quality scores less than 50, and small insertions and deletions (indels) with scores less than 25, were discarded, as was all variation that was called ambiguously by the BWA program. Changes identified in the LSJ1 strain were compared to those in a total of seven N2-derived mutant lines that had been sequenced using the Illumina platform. Any that were present in 4 or more N2 lines were removed for separate analysis.

Larger insertions (up to 30bp) and deletions (up to 10kb) were detected using the Pindel program [41], http://www.ebi.ac.uk/kye/pindel/). All large deletions detected by Pindel were analysed, and insertions were filtered for quality scores above 35.

### Computational analysis of sequence changes

To analyze sequence polymorphisms we created a pipeline that highlighted exon changes, indicated genes affected, and provided details of coding changes. Sequencing data were compared to data from the wild strains CB4856 and ED3054 (generated from the same Illumina flow cells) to establish the ancestral status of each change. To analyze mutation effects we obtained the gene annotation for *C. elegans* reference genome WS203 from Ensembl (www.ensembl.org) and overlaid the N2-LSJ1 mutations. We separated mutations into those lying within and outside annotated protein-coding regions (we refer to the latter as non-coding) (Tables S1 and S2, Figure S1). For SNPs in protein coding genes, we determined whether they caused synonymous or non-synonymous codon substitutions, or resided in UTR regions (Tables S1 and S2, Figure S1).

### Behavioral and Life-History Assays

Unless otherwise indicated, results for phenotypic differences were assessed with the Lilliefors test for normality. Provided results were normal, significances were calculated using a Student's *t* Test and P values adjusted posthoc with a Bonferroni correction. Cases where data was not normal and other tests were necessary are noted in figure legends. Statistics were calculated in R version 2.9.2.

Brood size and developmental rate: Individual L4 worms were placed on fresh NGM plates seeded with OP50. Adults were transferred each day to fresh plates until animals stopped producing eggs. The number of eggs laid each day was recorded, and the total number of eggs summed to give brood size.

Lifespan: L4 worms of each strain were transferred to fresh plates until they stopped producing progeny. Worms were observed daily and the number of dead individuals recorded.

Embryonic developmental rate: Embryos were dissected from gravid hermaphrodites by placing worms in M9 and cutting with a scalpel. Dissected embryos were washed with M9, placed in M9 on a coverslip sealed with wax, and imaged every five minutes for 10 hours using an LSM710 confocal microscope at 22°C. Developmental rate was assessed for each embryo by recording the time it took for comma stage embryos to hatch.

Egg staging: 10 L4 worms were transferred to fresh NGM plates 20–26 hours before the assay. The resulting adults were transferred to fresh plates and allowed to lay eggs for approximately 1 hour. Plates were then examined under a Leica M165FC dissecting microscope at 24x magnification and the stage of each egg recorded using the categories: 2–7 cell, 8–16 cell, >16 cells.

Aggregation: Approximately 40 animals were transferred to NGM plates that had been seeded with circular OP50 lawns at least two days prior to the experiment. Worms were left for 1 hour, and the number of animals on and off the border of the food, as well as the number in aggregates (groups of 2 or more worms), was recorded.

Oxygen responses: Locomotion assays were carried out essentially as described previously [31] except that gas flow was controlled by a custom built manifold and flow regulator.

### Genetic Mapping

Recombinant inbred lines between N2 and LSJ1 were constructed by crossing N2 males with LSJ1 hermaphrodites, and vice versa. F1 males were crossed with F1 hermaphrodites, and 50 F2 hermaphrodites were selected from each lineage (N2 or LSJ1 males in the P0 generation) to give a total of 100 recombinant inbred lines. These were selfed for 7 generations to give homozygosity at most genomic locations, and the resulting lines were genotyped using SNP-snip markers and phenotyped for early egg laying. Correlation between LSJ1 genotype and early egg laying phenotype was assessed using a linear model and a one-way ANOVA. LSJ1 DNA on chromosomes II and V was introgressed into N2 by repeated backcrossing with balancer strains CB1870 (chromosome II), CB2030 and CB2065 (chromosome V) for 7 generations and testing the resulting heterozygotes and homozygotes for early egg laying. As a control, we also tested N2 backcrossed with the above balancers. To further map the chromosome V loci, we picked CB2030 and CB2065 recombinants, analysed their recombination break points using SNP genotyping, and recorded their phenotypes when balanced with N2, or with another recombinant.

## Supporting Information

Figure S1 Computational pipeline used to analyze sequencing data: on the left of the flowchart are steps used to study large insertion/deletions, and on the right those used to study SNPs.
(9.65 MB TIF)

Figure S2 Mutational bias in N2 and LSJ1 lineages: (a) shows the overall number, as well as the number with N2 or LSJ1 as the ancestral state, for each class of substitution mutation and (b) gives the transition/transversion ratio overall, as well as for changes with each individual ancestral state.
(8.29 MB TIF)

Figure S3 Mapping of the LSJ1 early egg laying phenotype. (a) shows correlation between LSJ1 genotype and phenotype based on a linear model (significance assessed with a one-way ANOVA). The LSJ1 early egg laying phenotype is dominant (b, n = 6 for N2 and LSJ1; n = 8 for hets). Results fitted to a binomial model and assessed with an ANOVA (2 degrees of freedom, F = 35.95, P = 1.80×10–5). Pairwise comparisons made with a Mann-Whitney test, and P values were adjusted using a Bonferroni correction. The phenotype can be weakly recapitulated by LSJ1 DNA on chromosome II, and more strongly so by LSJ1 DNA on chromosome V (c, n = 43 for N2; n = 41 for LSJ1; n = 49 for LSJ1 on Chr II; n = 111 for LSJ1 on Chr V; n = 18 for transheterozygotes). Asterisks indicate significances compared to N2 under identical conditions. ns = not significant; * indicates p<0.01; ** indicates p<0.001; and *** indicates p<0.0001.
(8.96 MB TIF)

Table S1 Putative errors in the N2 reference sequence. A SNP or small indel was annotated as an error if it was found in at least four of six N2-derived strains used for comparison.
(1.88 MB DOC)

Table S2 N2/LSJ1 changes accumulated during domestication: SNPs and small indels between N2 and LSJ1 that are retained after filtering for N2 reference sequence errors.
(1.29 MB DOC)

Table S3 Larger insertions and deletions found between N2 and LSJ1: size indicates number of bases inserted or deleted. Deletions include the starting coordinate, and insertions occur after this coordinate.
(0.03 MB DOC)

Table S4 Predicted reference errors that alter genome annotation: subset of N2 reference sequence errors that are predicted to affect protein coding predictions.
(0.31 MB DOC)

Table S5 N2/LSJ1 protein coding changes: SNPs and small indels between N2 and LSJ1 that are predicted to affect protein coding.
(0.15 MB DOC)
