# Supplementary figures and images for: Whole Genome Sequencing Highlights Genetic Changes Associated with Laboratory Domestication of C. elegans
Source: PLoS One. 2010 Nov 11;5(11):e13922. doi: 10.1371/journal.pone.0013922 (PMC2978686; doi:10.1371/journal.pone.0013922)

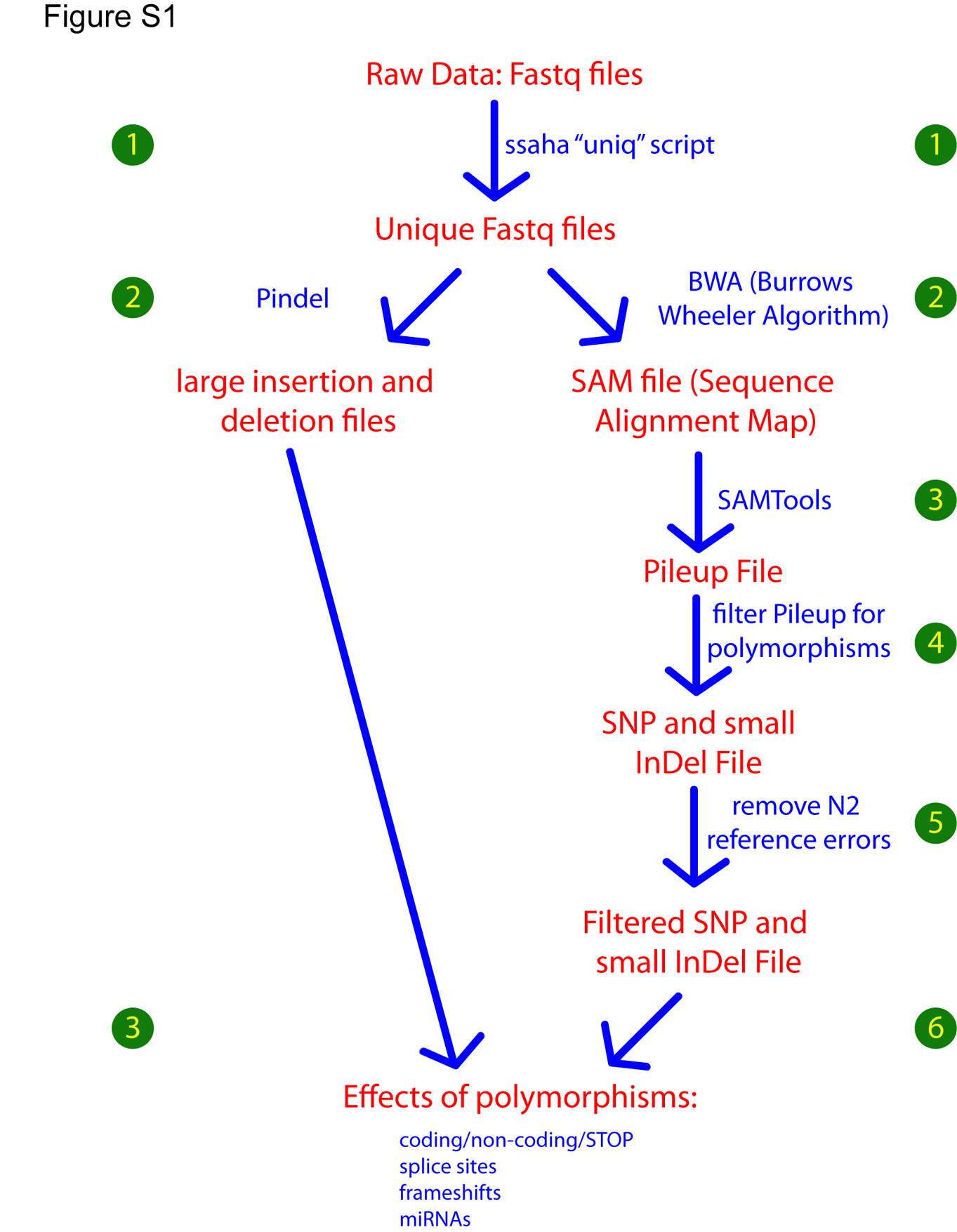

Supplement: Figure S1 — Computational pipeline used to analyze sequencing data: on the left of the flowchart are steps used to study large insertion/deletions, and on the right those used to study SNPs. (9.65 MB TIF) [file pone.0013922.s001.tif]

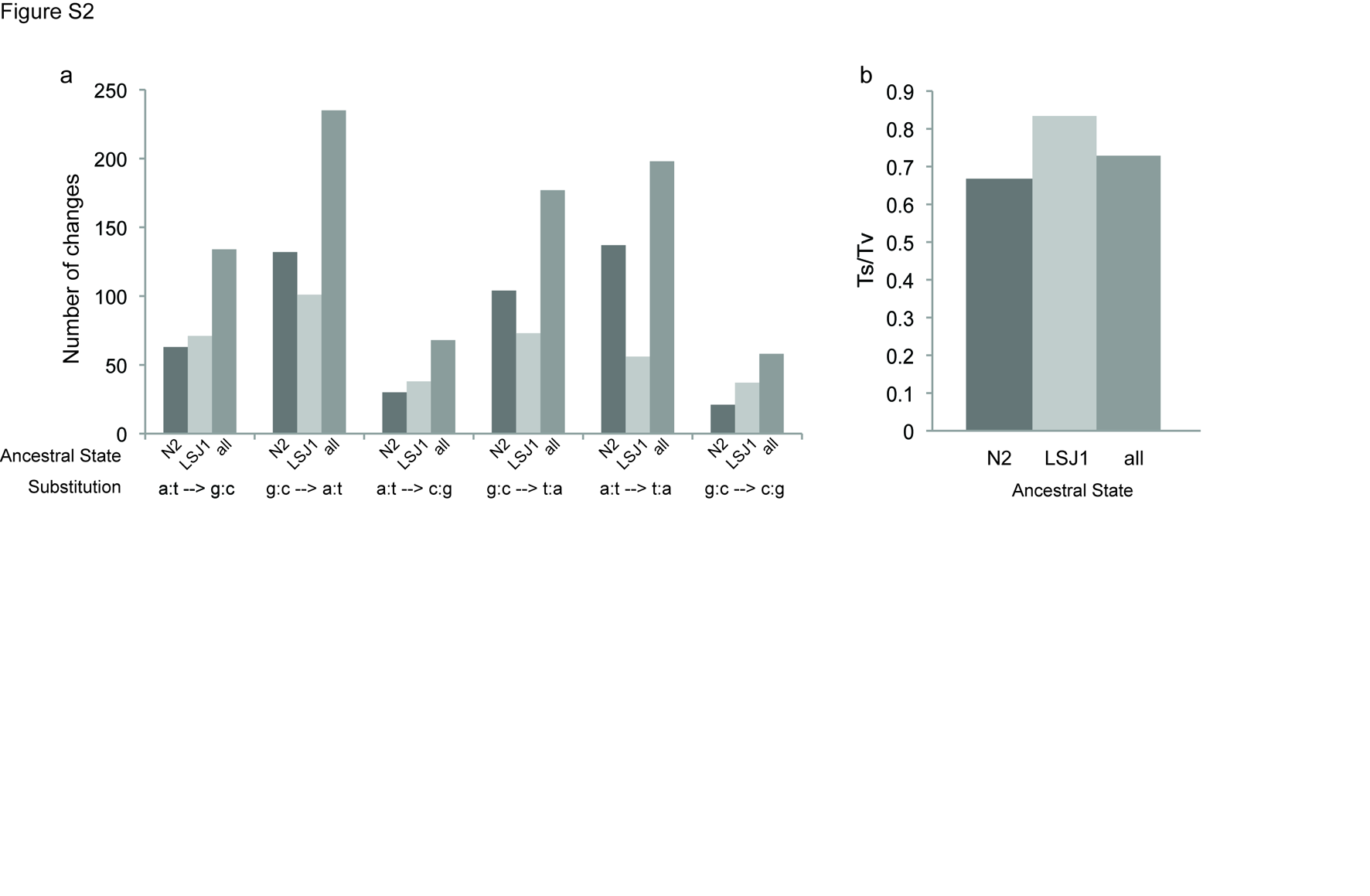

Supplement: Figure S2 — Mutational bias in N2 and LSJ1 lineages: (a) shows the overall number, as well as the number with N2 or LSJ1 as the ancestral state, for each class of substitution mutation and (b) gives the transition/transversion ratio overall, as well as for changes with each individual ancestral state. (8.29 MB TIF) [file pone.0013922.s002.tif]

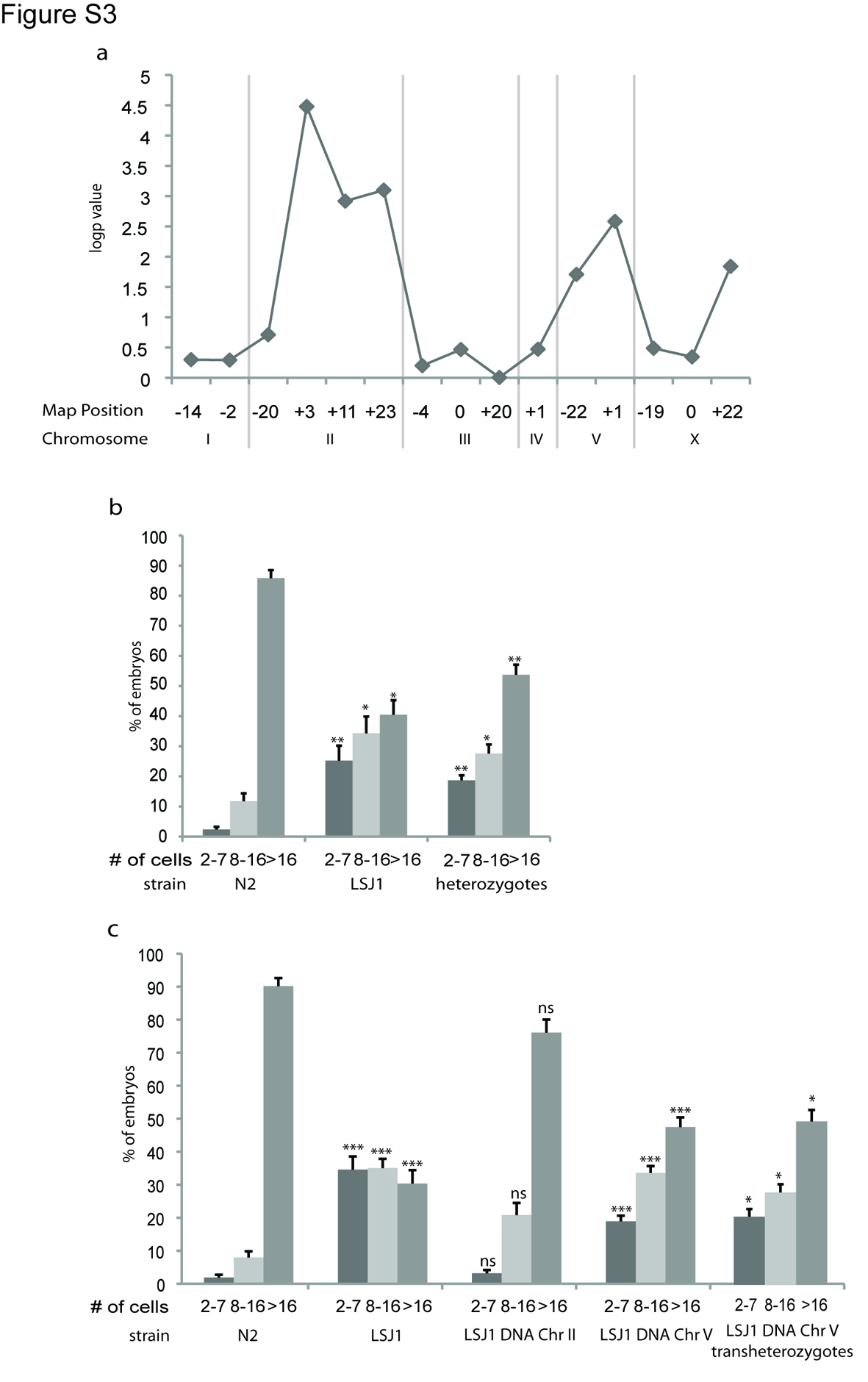

Supplement: Figure S3 — Mapping of the LSJ1 early egg laying phenotype. (a) shows correlation between LSJ1 genotype and phenotype based on a linear model (significance assessed with a one-way ANOVA). The LSJ1 early egg laying phenotype is dominant (b, n = 6 for N2 and LSJ1; n = 8 for hets). Results fitted to a binomial model and assessed with an ANOVA (2 degrees of freedom, F = 35.95, P = 1.80×10^–5^). Pairwise comparisons made with a Mann-Whitney test, and P values were adjusted using a Bonferroni correction. The phenotype can be weakly recapitulated by LSJ1 DNA on chromosome II, and more strongly so by LSJ1 DNA on chromosome V (c, n = 43 for N2; n = 41 for LSJ1; n = 49 for LSJ1 on Chr II; n = 111 for LSJ1 on Chr V; n = 18 for transheterozygotes). Asterisks indicate significances compared to N2 under identical conditions. ns = not significant; * indicates p<0.01; ** indicates p<0.001; and *** indicates p<0.0001. (8.96 MB TIF) [file pone.0013922.s003.tif]
