# Supplementary material for: Whole Genome Sequencing Highlights Genetic Changes Associated with Laboratory Domestication of C. elegans
Source: PLoS One. 2010 Nov 11;5(11):e13922. doi: 10.1371/journal.pone.0013922 (PMC2978686; doi:10.1371/journal.pone.0013922)
Supplement: Table S1 — Putative errors in the N2 reference sequence. A SNP or small indel was annotated as an error if it was found in at least four of six N2-derived strains used for comparison. (1.88 MB DOC) [file pone.0013922.s004.doc]

| Chromosome | Coordinate (WS203) | Reference Allele | Actual Allele | Type (Gene) |
| --- | --- | --- | --- | --- |
| I | 200948 | a | T | Non-coding |
| I | 200949 | c | T | Non-coding |
| I | 221369 | * | +G | exonic (Y48G1BL.6) |
| I | 232037 | * | -G | exonic (Y48G1BM.6) |
| I | 288367 | * | -T | Non-coding |
| I | 316117 | t | G | Non-coding |
| I | 337950 | * | -A | Non-coding |
| I | 341299 | * | -G | Non-coding |
| I | 361325 | c | A | Synonymous (R119.1) |
| I | 369897 | t | C | Non-coding |
| I | 714648 | g | C | Non-coding |
| I | 714649 | c | G | Non-coding |
| I | 742848 | * | +C | exonic (col-46) |
| I | 774905 | * | +T | Non-coding |
| I | 968802 | g | C | Non-coding |
| I | 1009208 | a | C | Non-coding |
| I | 1074824 | * | +C | exonic (*gsa-1*) |
| I | 1100872 | t | G | Non-coding |
| I | 1142103 | c | A | Nonsynonymous (Y48G8AL.10) |
| I | 1146687 | * | +G | Non-coding |
| I | 1225883 | * | +C | Non-coding |
| I | 1335729 | a | C | Non-coding |
| I | 1441965 | * | +A | Non-coding |
| I | 1580676 | * | -CCGAT | Non-coding |
| I | 1696288 | * | +A | Non-coding |
| I | 1748222 | * | +T | Non-coding |
| I | 1753323 | * | -T | Non-coding |
| I | 1864004 | t | C | Non-coding |
| I | 1954103 | * | +A | Non-coding |
| I | 1957150 | * | -G | Non-coding |
| I | 2033985 | * | +A | Non-coding |
| I | 2160529 | * | +A | Non-coding |
| I | 2211660 | * | -C | Non-coding |
| I | 2379643 | c | T | Nonsynonymous (Y39G10AR.17) |
| I | 2568367 | c | T | Non-coding |
| I | 2568368 | g | A | Non-coding |
| I | 2579329 | a | T | Non-coding |
| I | 2653302 | * | +C | Non-coding |
| I | 2710697 | t | C | exonic (Y71F9B.13) |
| I | 2777373 | c | G | Synonymous (Y71F9B.13) |
| I | 3002901 | * | -T | Non-coding |
| I | 3062018 | * | +A | Non-coding |
| I | 3146645 | c | T | Non-coding |
| I | 3259386 | * | -T | Non-coding |
| I | 3485154 | * | -C | Non-coding |
| I | 3690735 | * | +T | Non-coding |
| I | 3738201 | * | +G | Non-coding |
| I | 3885008 | a | G | Non-coding |
| I | 3885020 | a | G | Non-coding |
| I | 3929760 | * | +T | Non-coding |
| I | 3934999 | * | +G | Non-coding |
| I | 3935098 | t | C | Non-coding |
| I | 3935121 | a | C | Non-coding |
| I | 3935148 | g | C | Non-coding |
| I | 4007390 | * | +G | Non-coding |
| I | 4127917 | g | A | Non-coding |
| I | 4184637 | * | -A | exonic (ZK39.9) |
| I | 4220893 | * | +T | Non-coding |
| I | 4476753 | c | A | Non-coding |
| I | 4765803 | g | T | Non-coding |
| I | 4807829 | * | +A | Non-coding |
| I | 5003390 | * | +C | exonic (*dom-3*) |
| I | 5042624 | * | -GGT | exonic (C46H11.7) |
| I | 5056751 | * | +A | Non-coding |
| I | 5153922 | a | T | Non-coding |
| I | 5221052 | c | A | Non-coding |
| I | 5301101 | c | A | exonic (*let-526*) |
| I | 5357113 | * | -T | Non-coding |
| I | 5448372 | a | T | Non-coding |
| I | 5451259 | g | A | Non-coding |
| I | 5590737 | * | +C | Non-coding |
| I | 5910283 | * | +A | Non-coding |
| I | 6139031 | a | T | Non-coding |
| I | 6151524 | * | -CCG | Non-coding |
| I | 6163210 | t | A | Non-coding |
| I | 6163291 | * | -G | Non-coding |
| I | 6506262 | * | +T | Non-coding |
| I | 6801828 | * | +CT | exonic (E02D9.1) |
| I | 6820252 | t | C | Non-coding |
| I | 6949307 | a | G | Non-coding |
| I | 6949331 | t | A | Non-coding |
| I | 7140681 | g | T | Non-coding |
| I | 7140819 | t | C | Non-coding |
| I | 7140831 | a | T | Non-coding |
| I | 7149814 | * | +T | Non-coding |
| I | 7251392 | * | -A | Non-coding |
| I | 7301404 | c | G | Synonymous (*ttx-7*) |
| I | 7363706 | * | +G | Non-coding |
| I | 7421944 | * | +G | Non-coding |
| I | 7428554 | t | C | Non-coding |
| I | 7537821 | * | +C | exonic (*dcp-66*) |
| I | 7547117 | a | T | Non-coding |
| I | 7547127 | a | T | Non-coding |
| I | 7547365 | c | A | Non-coding |
| I | 7666813 | * | +G | Non-coding |
| I | 7753374 | * | +C | Non-coding |
| I | 7869008 | t | A | Non-coding |
| I | 7869009 | c | T | Non-coding |
| I | 7879119 | * | +T | Non-coding |
| I | 7879475 | c | T | Non-coding |
| I | 7879517 | g | A | Non-coding |
| I | 7879522 | a | T | Non-coding |
| I | 7950014 | * | +C | Non-coding |
| I | 8161379 | * | +G | Non-coding |
| I | 8213805 | t | A | Non-coding |
| I | 8280211 | * | +G | Non-coding |
| I | 8347872 | * | -C | Non-coding |
| I | 8359105 | * | +T | Non-coding |
| I | 8664613 | a | C | Non-coding |
| I | 8699345 | * | +G | Non-coding |
| I | 8818570 | * | +A | Non-coding |
| I | 9039720 | a | G | Non-coding |
| I | 9235193 | c | T | Non-coding |
| I | 9235318 | g | A | Non-coding |
| I | 9235427 | g | A | Non-coding |
| I | 9262895 | * | -G | Non-coding |
| I | 9276105 | * | +C | Non-coding |
| I | 9346153 | a | C | Non-coding |
| I | 9346156 | t | C | Non-coding |
| I | 9357394 | a | G | Non-coding |
| I | 9383156 | g | T | Non-coding |
| I | 9384408 | * | -C | Non-coding |
| I | 9457731 | g | A | Non-coding |
| I | 9468861 | * | +T | Non-coding |
| I | 9494581 | * | +AAG | Non-coding |
| I | 9632894 | * | +A | Non-coding |
| I | 9696378 | * | +T | Non-coding |
| I | 9699160 | t | C | Nonsynonymous (C04F12.8) |
| I | 9737906 | c | A | Non-coding |
| I | 9903066 | * | +G | Non-coding |
| I | 9940580 | a | T | Non-coding |
| I | 9949833 | * | -T | Non-coding |
| I | 9952382 | * | +G | Non-coding |
| I | 10030925 | * | +T | Non-coding |
| I | 10109254 | t | A | Non-coding |
| I | 10113278 | * | -G | exonic (R12E2.1) |
| I | 10188886 | t | C | Nonsynonymous (*dao-5*) |
| I | 10229800 | * | +T | Non-coding |
| I | 10243052 | * | -G | Non-coding |
| I | 10315724 | * | +A | Non-coding |
| I | 10436458 | * | -T | Non-coding |
| I | 10489711 | * | +C | Non-coding |
| I | 10495073 | * | +G | Non-coding |
| I | 10595968 | t | C | Non-coding |
| I | 10849083 | * | -CGAT | Non-coding |
| I | 10950940 | t | A | Non-coding |
| I | 10978887 | a | G | Synonymous (Y52B11A.3) |
| I | 11001775 | c | A | Non-coding |
| I | 11038967 | * | +T | Non-coding |
| I | 11145687 | * | +G | exonic (C18H9.6) |
| I | 11220200 | c | A | Non-coding |
| I | 11298166 | * | -C | Non-coding |
| I | 11386650 | * | +C | Non-coding |
| I | 11408100 | * | +T | Non-coding |
| I | 11424338 | * | +G | Non-coding |
| I | 11505538 | * | -T | Non-coding |
| I | 11522476 | * | +G | Non-coding |
| I | 11603465 | a | G | Non-coding |
| I | 11628456 | a | G | Non-coding |
| I | 11665162 | * | +T | Non-coding |
| I | 11764538 | * | +C | exonic (*vab-10*) |
| I | 11880752 | * | +GAGC | Non-coding |
| I | 11883806 | g | C | Non-coding |
| I | 11905377 | t | C | Non-coding |
| I | 11975955 | a | T | Non-coding |
| I | 11975961 | c | A | Non-coding |
| I | 11988102 | c | T | Non-coding |
| I | 12344912 | * | +T | Non-coding |
| I | 12388789 | * | +T | Non-coding |
| I | 12724433 | * | +A | Non-coding |
| I | 12862392 | t | A | Non-coding |
| I | 12880362 | * | +C | Non-coding |
| I | 12916972 | * | +C | Non-coding |
| I | 13015757 | t | C | Non-coding |
| I | 13015767 | t | C | Non-coding |
| I | 13015797 | t | C | Non-coding |
| I | 13080824 | * | +T | Non-coding |
| I | 13101698 | * | -A | exonic (*rgs-4*) |
| I | 13118108 | t | C | Non-coding |
| I | 13146671 | * | -T | Non-coding |
| I | 13168446 | c | G | Nonsynonymous (F22G12.5) |
| I | 13419807 | t | A | Non-coding |
| I | 13482732 | g | T | Non-coding |
| I | 13491121 | * | +T | exonic (Y48G10A.1) |
| I | 13529942 | * | -T | Non-coding |
| I | 13545461 | * | -T | Non-coding |
| I | 13617730 | * | +T | exonic (Y106G6D.3) |
| I | 13644248 | * | +A | Non-coding |
| I | 13736690 | * | -T | Non-coding |
| I | 13791934 | * | -T | Non-coding |
| I | 13860804 | c | T | Non-coding |
| I | 13860835 | g | C | Non-coding |
| I | 13985036 | a | G | Nonsynonymous (Y71A12B.17) |
| I | 14049137 | t | C | Non-coding |
| I | 14097922 | t | C | Non-coding |
| I | 14121546 | * | -T | Non-coding |
| I | 14169343 | * | +C | Non-coding |
| I | 14244891 | g | C | Non-coding |
| I | 14326084 | c | T | Non-coding |
| I | 14326170 | g | A | Non-coding |
| I | 14326171 | c | T | Non-coding |
| I | 14347419 | c | A | Non-coding |
| I | 14349944 | * | +C | Non-coding |
| I | 14362092 | g | T | Non-coding |
| I | 14363822 | a | T | Non-coding |
| I | 14363832 | t | C | Non-coding |
| I | 14376946 | * | -T | Non-coding |
| I | 14431046 | c | A | Non-coding |
| I | 14480479 | g | A | Non-coding |
| I | 14518026 | c | G | Non-coding |
| I | 14586339 | a | C | Non-coding |
| I | 14599329 | * | -A | Non-coding |
| I | 14624750 | * | +G | Non-coding |
| I | 14675915 | a | T | Non-coding |
| I | 14918627 | a | C | Non-coding |
| II | 22469 | a | T | Non-coding |
| II | 213638 | * | +G | exonic (F48A11.4) |
| II | 505419 | * | +GC | Non-coding |
| II | 523339 | * | +G | Non-coding |
| II | 595620 | * | -GCGGCCGC | Non-coding |
| II | 698492 | a | G | Non-coding |
| II | 751039 | * | +A | Non-coding |
| II | 846684 | * | +G | Non-coding |
| II | 935804 | a | G | Synonymous (*acs-15*) |
| II | 959061 | a | T | Non-coding |
| II | 1109664 | * | +A | Non-coding |
| II | 1214973 | * | +A | Non-coding |
| II | 1221572 | * | +A | Non-coding |
| II | 1333323 | c | G | Non-coding |
| II | 1341491 | * | +A | Non-coding |
| II | 1388541 | * | +A | Non-coding |
| II | 1392498 | t | C | Non-coding |
| II | 1451277 | a | G | Non-coding |
| II | 1577786 | * | +G | exonic (C17F4.3) |
| II | 1580403 | * | +GA | Non-coding |
| II | 1592018 | * | +T | Non-coding |
| II | 1602995 | * | +G | Non-coding |
| II | 1628716 | * | +C | Non-coding |
| II | 1637578 | c | G | Non-coding |
| II | 1637579 | g | C | Non-coding |
| II | 1804984 | t | A | Non-coding |
| II | 1848045 | * | +C | exonic (*fbxb-97*) |
| II | 1850708 | t | C | Non-coding |
| II | 1906686 | * | -GATC | Non-coding |
| II | 1926839 | * | +C | exonic (F52C6.2) |
| II | 1952284 | a | T | Non-coding |
| II | 2001658 | a | C | Non-coding |
| II | 2037270 | * | -A | Non-coding |
| II | 2190806 | t | C | Non-coding |
| II | 2472169 | c | T | Non-coding |
| II | 2564356 | * | +G | Non-coding |
| II | 2642036 | * | +A | Non-coding |
| II | 2678738 | * | +A | exonic (F22E5.17) |
| II | 2796451 | * | +G | exonic (F49C5.11) |
| II | 2799760 | a | T | Non-coding |
| II | 2873149 | * | +T | Non-coding |
| II | 2879045 | * | +G | exonic (Y110A2AL.12) |
| II | 2900852 | * | -AG | Non-coding |
| II | 2900872 | c | A | Non-coding |
| II | 2900873 | g | T | Non-coding |
| II | 2925946 | * | +C | exonic (ZK355.2) |
| II | 2937465 | * | +A | Non-coding |
| II | 2942796 | * | +C | Non-coding |
| II | 3168542 | * | +G | Non-coding |
| II | 3241680 | * | -A | exonic (VM106R.1) |
| II | 3278964 | t | A | Non-coding |
| II | 3321964 | * | +G | Non-coding |
| II | 3521235 | * | +C | exonic (*col-72*) |
| II | 3531439 | * | +G | Non-coding |
| II | 3532125 | c | G | Non-coding |
| II | 3532191 | g | C | Non-coding |
| II | 3532196 | t | C | Non-coding |
| II | 3650567 | c | A | Non-coding |
| II | 3796665 | * | +C | Non-coding |
| II | 3927545 | * | -T | Non-coding |
| II | 3946669 | * | -A | Non-coding |
| II | 4142478 | c | G | Non-coding |
| II | 4142481 | g | A | Non-coding |
| II | 4142501 | a | T | Non-coding |
| II | 4260994 | t | C | Non-coding |
| II | 4273307 | * | +C | Non-coding |
| II | 4296767 | * | +G | Non-coding |
| II | 4321910 | * | -A | Non-coding |
| II | 4332861 | * | -A | Non-coding |
| II | 4349464 | * | +A | Non-coding |
| II | 4373840 | * | +G | Non-coding |
| II | 4375084 | * | +C | Non-coding |
| II | 4381937 | * | +G | Non-coding |
| II | 4387825 | * | +CG | Non-coding |
| II | 4390787 | * | +C | Non-coding |
| II | 4398910 | * | +C | Non-coding |
| II | 4399212 | g | C | Non-coding |
| II | 4468036 | g | T | Non-coding |
| II | 4468046 | g | T | Non-coding |
| II | 4478953 | * | +C | Non-coding |
| II | 4484306 | * | +C | Non-coding |
| II | 4492562 | * | +A | Non-coding |
| II | 4499374 | g | C | Non-coding |
| II | 4510371 | * | -T | Non-coding |
| II | 4510486 | * | +C | Non-coding |
| II | 4578455 | * | -A | Non-coding |
| II | 4737514 | * | +C | Non-coding |
| II | 4754157 | * | -G | exonic (C27D9.1) |
| II | 4763294 | * | -A | exonic (*ehs-1*) |
| II | 4782899 | * | +G | Non-coding |
| II | 4831916 | * | +G | Non-coding |
| II | 4841268 | * | -C | Non-coding |
| II | 4861726 | * | +G | Non-coding |
| II | 4919736 | * | +T | Non-coding |
| II | 4921070 | * | +C | Non-coding |
| II | 4929759 | c | A | Non-coding |
| II | 4964576 | c | A | Non-coding |
| II | 5006737 | * | +G | exonic (F59A6.4) |
| II | 5021686 | * | +G | Non-coding |
| II | 5028911 | * | -C | Non-coding |
| II | 5078768 | * | +G | Non-coding |
| II | 5079263 | * | +C | exonic (*dsh-2*) |
| II | 5096459 | * | +G | Non-coding |
| II | 5125113 | * | -G | Non-coding |
| II | 5136067 | * | +C | Non-coding |
| II | 5151326 | * | +T | Non-coding |
| II | 5185654 | g | C | Synonymous (C27D6.4) |
| II | 5191923 | * | +G | Non-coding |
| II | 5203315 | * | +G | Non-coding |
| II | 5205716 | * | +C | Non-coding |
| II | 5219639 | * | +G | exonic (F41G3.10) |
| II | 5226379 | * | +C | Non-coding |
| II | 5231216 | t | G | Non-coding |
| II | 5231227 | t | G | Non-coding |
| II | 5231250 | a | C | Non-coding |
| II | 5248363 | * | +C | Non-coding |
| II | 5258815 | * | +T | Non-coding |
| II | 5268627 | * | -G | Non-coding |
| II | 5268766 | * | +T | Non-coding |
| II | 5268821 | * | +T | Non-coding |
| II | 5268863 | * | +T | Non-coding |
| II | 5271459 | * | +G | exonic (*drn-1*) |
| II | 5322089 | t | A | Non-coding |
| II | 5563746 | * | +G | Non-coding |
| II | 5594346 | * | +G | Non-coding |
| II | 5635095 | c | G | Synonymous (F59E12.8) |
| II | 5647429 | c | T | Nonsynonymous (ddl-1) |
| II | 5661840 | * | -C | Non-coding |
| II | 5667682 | * | +G | exonic (C25H3.7) |
| II | 5670253 | * | +C | exonic (C25H3.8) |
| II | 5679958 | * | +C | Non-coding |
| II | 5695889 | * | +G | Non-coding |
| II | 5707675 | * | +C | Non-coding |
| II | 5723522 | g | T | Non-coding |
| II | 5732897 | * | +T | Non-coding |
| II | 5775938 | * | -C | Non-coding |
| II | 5779230 | * | -C | Non-coding |
| II | 5814272 | * | +G | exonic (ZK1248.9) |
| II | 5820840 | * | -G | exonic (*ehs-1*) |
| II | 5846224 | * | -G | Non-coding |
| II | 5861709 | * | +A | Non-coding |
| II | 5863661 | * | -C | Non-coding |
| II | 5863711 | * | +A | Non-coding |
| II | 5863797 | * | +A | Non-coding |
| II | 5881905 | * | +G | Non-coding |
| II | 5931438 | * | +C | Non-coding |
| II | 5941664 | * | +G | Non-coding |
| II | 5960458 | c | T | Non-coding |
| II | 5966352 | * | +T | Non-coding |
| II | 5967031 | * | -C | Non-coding |
| II | 5974545 | * | +T | exonic (B0034.5) |
| II | 5978556 | * | +G | Non-coding |
| II | 5983704 | * | -C | Non-coding |
| II | 5990758 | * | +C | Non-coding |
| II | 5992169 | * | +T | Non-coding |
| II | 5998649 | * | +C | exonic (*ins-5*) |
| II | 6003329 | * | -C | Non-coding |
| II | 6039980 | * | +C | Non-coding |
| II | 6057199 | * | +C | Non-coding |
| II | 6099641 | * | +C | Non-coding |
| II | 6118994 | * | +A | Non-coding |
| II | 6186714 | * | +T | Non-coding |
| II | 6193649 | * | +G | exonic (*cpb-2*) |
| II | 6200178 | * | +G | exonic (*tag-319*) |
| II | 6205127 | * | +A | Non-coding |
| II | 6206073 | * | +C | Non-coding |
| II | 6208444 | * | +C | Non-coding |
| II | 6256077 | * | +G | Non-coding |
| II | 6317273 | * | +T | Non-coding |
| II | 6451387 | a | T | Non-coding |
| II | 6500691 | * | +G | exonic (R05G9.3) |
| II | 6522778 | * | +C | Non-coding |
| II | 6527423 | g | A | Synonymous (C56E6.4) |
| II | 6527424 | a | T | Synonymous (C56E6.4) |
| II | 6533053 | * | -T | Non-coding |
| II | 6535277 | * | -T | Non-coding |
| II | 6547248 | * | +G | Non-coding |
| II | 6559522 | * | +C | exonic (F18C5.10) |
| II | 6561612 | * | +C | Non-coding |
| II | 6616257 | * | +C | Non-coding |
| II | 6620906 | * | +G | Non-coding |
| II | 6640323 | * | +G | Non-coding |
| II | 6656983 | * | +C | Non-coding |
| II | 6657794 | * | +A | Non-coding |
| II | 6700726 | * | +G | exonic (*math-38*) |
| II | 6713193 | * | +G | Non-coding |
| II | 6713476 | * | +G | Non-coding |
| II | 6716318 | * | +G | Non-coding |
| II | 6718340 | * | +C | Non-coding |
| II | 6719832 | * | -C | Non-coding |
| II | 6720673 | * | +C | Non-coding |
| II | 6721747 | * | -A | Non-coding |
| II | 6724147 | * | +C | Non-coding |
| II | 6725102 | * | +G | Non-coding |
| II | 6727389 | * | +G | Non-coding |
| II | 6729750 | * | +T | Non-coding |
| II | 6730801 | * | -C | Non-coding |
| II | 6730839 | * | -C | Non-coding |
| II | 6736304 | c | G | Nonsynonymous (T14B4.2) |
| II | 6736306 | t | C | Nonsynonymous (T14B4.2) |
| II | 6737045 | * | -C | Non-coding |
| II | 6745223 | * | +C | Non-coding |
| II | 6749216 | * | -G | exonic (F26G1.2) |
| II | 6750712 | * | +G | exonic (F41G3.20) |
| II | 6755508 | * | -C | Non-coding |
| II | 6755908 | a | T | Non-coding |
| II | 6759797 | * | -A | Non-coding |
| II | 6759907 | * | +T | exonic (F41G3.2) |
| II | 6762603 | c | T | Non-coding |
| II | 6764953 | * | +C | Non-coding |
| II | 6766065 | c | T | Non-coding |
| II | 6766211 | c | T | Non-coding |
| II | 6808829 | a | G | Non-coding |
| II | 6818451 | * | +G | Non-coding |
| II | 6878030 | * | +T | Non-coding |
| II | 6878056 | c | T | Non-coding |
| II | 6896314 | t | A | Non-coding |
| II | 6908985 | c | T | Non-coding |
| II | 6930815 | * | +T | Non-coding |
| II | 6940137 | * | +T | Non-coding |
| II | 6942554 | * | -G | Non-coding |
| II | 6944908 | * | -G | Non-coding |
| II | 6950739 | * | -G | Non-coding |
| II | 6999734 | c | A | Non-coding |
| II | 7003246 | t | C | Non-coding |
| II | 7003347 | t | A | Nonsynonymous (*unc-104*) |
| II | 7028425 | * | +A | Non-coding |
| II | 7123163 | t | C | Non-coding |
| II | 7125567 | * | +G | Non-coding |
| II | 7141341 | * | -T | Non-coding |
| II | 7339647 | * | +C | Non-coding |
| II | 7343869 | * | +G | Non-coding |
| II | 7397588 | * | +G | Non-coding |
| II | 7398344 | * | -G | Non-coding |
| II | 7403159 | * | -C | Non-coding |
| II | 7422319 | * | +C | Non-coding |
| II | 7444114 | * | +C | Non-coding |
| II | 7458065 | * | +G | Non-coding |
| II | 7478839 | * | +C | Non-coding |
| II | 7479112 | * | +G | exonic (*ubc-6*) |
| II | 7486763 | * | +C | Non-coding |
| II | 7506632 | * | +G | Non-coding |
| II | 7511880 | * | +G | Non-coding |
| II | 7528855 | * | +C | Non-coding |
| II | 7547478 | * | +T | Non-coding |
| II | 7573659 | * | +G | Non-coding |
| II | 7584472 | * | +A | Non-coding |
| II | 7611925 | * | +A | Non-coding |
| II | 7617066 | * | -T | Non-coding |
| II | 7625206 | * | +G | Non-coding |
| II | 7634326 | * | +G | Non-coding |
| II | 7640260 | * | +G | Non-coding |
| II | 7640559 | * | +C | Non-coding |
| II | 7642076 | * | +CT | exonic (*pde-4*) |
| II | 7642132 | * | +CGCG | Non-coding |
| II | 7644340 | * | -C | Non-coding |
| II | 7673643 | * | +G | Non-coding |
| II | 7676550 | * | +GC | Non-coding |
| II | 7696463 | * | +A | Non-coding |
| II | 7698791 | * | +G | Non-coding |
| II | 7739392 | * | -C | exonic (*tag-308*) |
| II | 7743762 | * | -C | Non-coding |
| II | 7743865 | * | -T | Non-coding |
| II | 7754506 | * | -C | exonic (B0228.6) |
| II | 7799887 | * | -G | Non-coding |
| II | 7803626 | * | -A | Non-coding |
| II | 7809945 | * | +C | Non-coding |
| II | 7825758 | * | +C | Non-coding |
| II | 7866905 | * | +A | exonic (*ztf-17*) |
| II | 7871211 | * | -G | exonic (T01H3.3) |
| II | 7879633 | * | -T | Non-coding |
| II | 7883736 | * | +G | Non-coding |
| II | 7905708 | * | -A | Non-coding |
| II | 7913323 | c | A | Non-coding |
| II | 7960769 | c | G | Non-coding |
| II | 8033054 | * | +G | Non-coding |
| II | 8049767 | * | -AGT | Non-coding |
| II | 8187014 | * | -A | Non-coding |
| II | 8210521 | * | -G | Non-coding |
| II | 8227927 | g | C | Nonsynonymous (*dab-1*) |
| II | 8227928 | c | G | Nonsynonymous (*dab-1*) |
| II | 8360601 | * | +C | Non-coding |
| II | 8584093 | c | T | Nonsynonymous (F49C9.11) |
| II | 8622307 | * | -C | Non-coding |
| II | 8689936 | * | +T | Non-coding |
| II | 8715377 | c | T | Non-coding |
| II | 8742081 | * | +G | Non-coding |
| II | 8743061 | * | +G | Non-coding |
| II | 8748249 | * | +C | Non-coding |
| II | 8755591 | * | +G | Non-coding |
| II | 8759312 | * | +A | Non-coding |
| II | 8765136 | * | +G | exonic (*pah-1*) |
| II | 8798261 | * | +G | Non-coding |
| II | 8813855 | * | +T | Non-coding |
| II | 8819694 | * | +T | Non-coding |
| II | 8821799 | * | +T | Non-coding |
| II | 8824743 | * | +G | Non-coding |
| II | 8833583 | * | +C | Non-coding |
| II | 8867479 | * | +C | Non-coding |
| II | 8894561 | * | +GC | Non-coding |
| II | 8894955 | c | T | Non-coding |
| II | 8896591 | * | +C | Non-coding |
| II | 8903256 | * | +C | Non-coding |
| II | 8907371 | c | T | Non-coding |
| II | 8916730 | * | -G | Non-coding |
| II | 8916827 | * | +T | Non-coding |
| II | 9034132 | * | -T | Non-coding |
| II | 9090950 | * | +T | Non-coding |
| II | 9151874 | * | -T | Non-coding |
| II | 9181320 | * | -T | Non-coding |
| II | 9234922 | * | -C | exonic (T26C5.2) |
| II | 9299859 | * | -G | Non-coding |
| II | 9322171 | * | -T | Non-coding |
| II | 9418645 | * | +CG | Non-coding |
| II | 9419090 | * | +C | Non-coding |
| II | 9426638 | * | +A | Non-coding |
| II | 9427748 | * | +C | Non-coding |
| II | 9490844 | * | -G | Non-coding |
| II | 9493720 | * | -C | Non-coding |
| II | 9523710 | * | +G | Non-coding |
| II | 9527712 | * | +C | Non-coding |
| II | 9547898 | * | -T | Non-coding |
| II | 9576349 | * | +A | Non-coding |
| II | 9609995 | * | -G | Non-coding |
| II | 9669185 | * | +A | Non-coding |
| II | 9957412 | * | -A | Non-coding |
| II | 9964672 | * | +G | Non-coding |
| II | 9985750 | * | -T | Non-coding |
| II | 10206347 | * | +G | Non-coding |
| II | 10344175 | * | +T | Non-coding |
| II | 10427276 | * | -C | Non-coding |
| II | 10430279 | * | +G | Non-coding |
| II | 10432290 | * | -A | Non-coding |
| II | 10566496 | * | -G | Non-coding |
| II | 10599660 | * | -A | Non-coding |
| II | 10779219 | g | T | Non-coding |
| II | 10826672 | * | -A | Non-coding |
| II | 10826788 | * | -A | Non-coding |
| II | 10827270 | * | +T | Non-coding |
| II | 10829479 | * | +A | exonic (Y38E10A.8) |
| II | 10830904 | * | +T | Non-coding |
| II | 10918275 | * | -T | Non-coding |
| II | 10976598 | * | -A | Non-coding |
| II | 11031294 | * | -T | Non-coding |
| II | 11092046 | t | C | Non-coding |
| II | 11162421 | * | +C | Non-coding |
| II | 11167283 | * | +C | Non-coding |
| II | 11181038 | t | G | Non-coding |
| II | 11194995 | * | +C | Non-coding |
| II | 11196213 | * | +C | Non-coding |
| II | 11196929 | * | +G | Non-coding |
| II | 11200485 | * | +T | Non-coding |
| II | 11202838 | * | +G | Non-coding |
| II | 11207621 | * | +A | exonic (T06D8.1) |
| II | 11318249 | * | -A | Non-coding |
| II | 11384797 | * | +G | exonic (*shn-1*) |
| II | 11393903 | * | +CG | Non-coding |
| II | 11551005 | * | +A | Non-coding |
| II | 11554219 | * | +G | Non-coding |
| II | 11564862 | * | +GT | Non-coding |
| II | 11578010 | * | +GCGC | Non-coding |
| II | 11591620 | * | +G | Non-coding |
| II | 11611863 | * | -A | Non-coding |
| II | 11611945 | * | -A | Non-coding |
| II | 11701964 | * | -TT | Non-coding |
| II | 11702076 | * | -T | Non-coding |
| II | 11704839 | * | -T | Non-coding |
| II | 11717736 | * | +G | Non-coding |
| II | 11719602 | * | +C | Non-coding |
| II | 11719744 | * | +G | Non-coding |
| II | 11769563 | * | +T | Non-coding |
| II | 11794523 | a | C | Non-coding |
| II | 11890801 | * | +C | Non-coding |
| II | 11898730 | * | -A | Non-coding |
| II | 11938389 | * | +G | Non-coding |
| II | 11960766 | * | +G | Non-coding |
| II | 12096670 | * | +T | Non-coding |
| II | 12115353 | c | T | Non-coding |
| II | 12170815 | g | A | Non-coding |
| II | 12174493 | g | A | Non-coding |
| II | 12178809 | c | T | Non-coding |
| II | 12193173 | g | A | Non-coding |
| II | 12230087 | * | +G | Non-coding |
| II | 12266284 | * | -G | Non-coding |
| II | 12272898 | * | -G | Non-coding |
| II | 12297335 | * | +A | Non-coding |
| II | 12370055 | c | A | Non-coding |
| II | 12387897 | t | A | Non-coding |
| II | 12433906 | * | +G | Non-coding |
| II | 12556600 | * | +G | exonic (*dsh-1*) |
| II | 12562072 | * | +C | Non-coding |
| II | 12573640 | * | +G | Non-coding |
| II | 12588479 | * | +A | Non-coding |
| II | 12595553 | c | T | Non-coding |
| II | 12607617 | * | +A | exonic (*rgs-4*) |
| II | 12650481 | * | +G | Non-coding |
| II | 12656384 | * | -T | Non-coding |
| II | 12658929 | c | T | Non-coding |
| II | 12659508 | a | G | Non-coding |
| II | 12667009 | * | +C | Non-coding |
| II | 12668767 | * | +G | exonic (T06D8.1) |
| II | 12670938 | a | G | Non-coding |
| II | 12675697 | * | +A | Non-coding |
| II | 12677300 | * | +G | exonic (Y38E10A.23) |
| II | 12677412 | t | G | Nonsynonymous (Y38E10A.23) |
| II | 12683679 | a | T | Non-coding |
| II | 12684543 | g | A | Non-coding |
| II | 12684928 | g | A | Non-coding |
| II | 12688927 | * | +C | Non-coding |
| II | 12761838 | * | +A | Non-coding |
| II | 12764190 | g | C | Non-coding |
| II | 12764264 | * | -A | Non-coding |
| II | 12769724 | * | +C | Non-coding |
| II | 12784485 | t | C | Non-coding |
| II | 12784523 | * | +C | Non-coding |
| II | 12795898 | * | -G | Non-coding |
| II | 12798297 | * | +AC | Non-coding |
| II | 12800298 | * | -G | Non-coding |
| II | 12807213 | * | +T | Non-coding |
| II | 12808196 | * | +G | Non-coding |
| II | 12817524 | * | +C | Non-coding |
| II | 12824506 | * | -C | Non-coding |
| II | 12824537 | * | -C | Non-coding |
| II | 12829149 | * | +G | Non-coding |
| II | 12829859 | c | A | Non-coding |
| II | 12949259 | * | +G | Non-coding |
| II | 13023500 | * | +C | Non-coding |
| II | 13031555 | * | +T | Non-coding |
| II | 13034269 | * | +A | Non-coding |
| II | 13034379 | * | -A | Non-coding |
| II | 13062610 | t | A | Non-coding |
| II | 13063712 | * | -A | Non-coding |
| II | 13090005 | t | A | Nonsynonymous (*gcn-2*) |
| II | 13092153 | g | C | Non-coding |
| II | 13092162 | g | T | Non-coding |
| II | 13092217 | g | A | Non-coding |
| II | 13239531 | g | C | Synonymous (F15D4.4) |
| II | 13350116 | c | A | Non-coding |
| II | 13378340 | g | A | Non-coding |
| II | 13389059 | * | +A | Non-coding |
| II | 13434751 | g | T | Non-coding |
| II | 13434786 | g | T | Non-coding |
| II | 13440583 | g | T | Nonsynonymous (*tbc-15*) |
| II | 13452555 | a | G | Non-coding |
| II | 13485389 | t | C | Synonymous (E01G4.6) |
| II | 13504673 | * | +CGTCTG | Non-coding |
| II | 13528270 | a | G | Non-coding |
| II | 13578367 | a | G | Non-coding |
| II | 13579405 | t | A | Non-coding |
| II | 13627556 | * | +T | Non-coding |
| II | 13697889 | * | +C | Non-coding |
| II | 13715888 | * | +T | Non-coding |
| II | 13718787 | * | +T | Non-coding |
| II | 13735571 | * | -T | Non-coding |
| II | 13801366 | c | A | Non-coding |
| II | 13835091 | * | +GCC | exonic (*ins-37*) |
| II | 13883906 | * | +T | Non-coding |
| II | 13905901 | * | +G | Non-coding |
| II | 13946653 | g | A | Non-coding |
| II | 13946656 | t | A | Non-coding |
| II | 14021243 | g | T | Non-coding |
| II | 14044539 | * | +G | Non-coding |
| II | 14125531 | a | T | Non-coding |
| II | 14171676 | c | T | Non-coding |
| II | 14211019 | c | G | Non-coding |
| II | 14260657 | a | C | Non-coding |
| II | 14261823 | g | C | Non-coding |
| II | 14267620 | * | +G | Non-coding |
| II | 14269185 | * | +A | Non-coding |
| II | 14276713 | t | C | Synonymous (Y54G11A.3) |
| II | 14279456 | c | A | Non-coding |
| II | 14311576 | t | C | Non-coding |
| II | 14317222 | * | +C | Non-coding |
| II | 14406898 | t | C | Synonymous (F26H11.2) |
| II | 14464253 | * | -T | Non-coding |
| II | 14614142 | * | +A | Non-coding |
| II | 14738880 | * | +C | Non-coding |
| II | 14796259 | g | T | Nonsynonymous (*eif-3.B*) |
| II | 14872831 | c | A | Non-coding |
| II | 14981157 | g | A | Non-coding |
| II | 15043625 | c | G | Non-coding |
| II | 15073258 | t | A | Non-coding |
| II | 15188802 | * | +CAA | Non-coding |
| III | 38559 | * | +T | Non-coding |
| III | 118298 | t | G | Synonymous (C29F9.5) |
| III | 212540 | * | +A | Non-coding |
| III | 230726 | * | +GC | Non-coding |
| III | 480230 | * | +C | Non-coding |
| III | 513526 | * | +CG | Non-coding |
| III | 543652 | * | +C | Non-coding |
| III | 628763 | a | C | Non-coding |
| III | 661016 | * | +G | exonic (C09F5.1) |
| III | 665295 | * | +G | Non-coding |
| III | 665394 | * | +G | Non-coding |
| III | 666838 | * | +C | Non-coding |
| III | 672113 | * | +G | Non-coding |
| III | 691185 | c | T | Nonsynonymous (W02B3.7) |
| III | 705098 | * | +C | Non-coding |
| III | 712124 | * | +CG | Non-coding |
| III | 752175 | g | T | Non-coding |
| III | 758867 | * | -C | Non-coding |
| III | 758901 | * | -C | Non-coding |
| III | 791552 | * | +C | Non-coding |
| III | 792814 | * | +C | Non-coding |
| III | 803905 | * | -G | Non-coding |
| III | 805755 | * | +A | Non-coding |
| III | 811725 | * | +C | Non-coding |
| III | 821843 | * | +G | Non-coding |
| III | 835904 | * | +C | exonic (*dos-3*) |
| III | 840788 | * | +T | Non-coding |
| III | 844171 | * | +GC | Non-coding |
| III | 847237 | * | +GGC | exonic (K02F3.2) |
| III | 871577 | * | +G | Non-coding |
| III | 1028768 | * | -C | Non-coding |
| III | 1122191 | * | +A | Non-coding |
| III | 1153120 | * | -T | Non-coding |
| III | 1177841 | * | -C | Non-coding |
| III | 1233079 | * | +G | Non-coding |
| III | 1318411 | c | G | Non-coding |
| III | 1323866 | g | A | Non-coding |
| III | 1600168 | * | +T | Non-coding |
| III | 1629052 | c | T | Non-coding |
| III | 1636696 | g | A | Non-coding |
| III | 1636742 | c | A | Non-coding |
| III | 1636775 | g | A | Non-coding |
| III | 1636782 | g | T | Non-coding |
| III | 1863224 | g | T | Non-coding |
| III | 1961517 | * | +G | Non-coding |
| III | 2082758 | * | +G | Non-coding |
| III | 2253911 | * | +A | Non-coding |
| III | 2327178 | * | -C | Non-coding |
| III | 2365479 | t | C | Non-coding |
| III | 2365480 | a | G | Non-coding |
| III | 2405323 | c | T | Non-coding |
| III | 2541506 | * | -C | Non-coding |
| III | 2565617 | g | A | Non-coding |
| III | 2645098 | * | -G | Non-coding |
| III | 2653086 | * | -G | Non-coding |
| III | 2728181 | a | T | Non-coding |
| III | 2809673 | a | G | Non-coding |
| III | 2822658 | g | C | Non-coding |
| III | 2980599 | a | T | Non-coding |
| III | 3005144 | * | -A | Non-coding |
| III | 3161750 | * | +G | Non-coding |
| III | 3162633 | a | G | Non-coding |
| III | 3162638 | a | G | Non-coding |
| III | 3254400 | g | C | Non-coding |
| III | 3255111 | c | A | Non-coding |
| III | 3255125 | c | T | Non-coding |
| III | 3255131 | g | T | Non-coding |
| III | 3271710 | a | G | Non-coding |
| III | 3271738 | t | A | Non-coding |
| III | 3281309 | * | +C | Non-coding |
| III | 3304492 | * | +T | Non-coding |
| III | 3339894 | * | -C | Non-coding |
| III | 3346935 | * | +G | Non-coding |
| III | 3347667 | * | +G | Non-coding |
| III | 3347714 | * | +C | Non-coding |
| III | 3348162 | * | +T | Non-coding |
| III | 3355706 | * | -T | Non-coding |
| III | 3366747 | * | +G | exonic (*prk-2*) |
| III | 3373714 | g | A | Non-coding |
| III | 3377952 | * | +G | Non-coding |
| III | 3378283 | * | +G | Non-coding |
| III | 3390531 | * | +G | Non-coding |
| III | 3426070 | * | -A | Non-coding |
| III | 3429127 | a | C | Non-coding |
| III | 3430900 | * | +C | Non-coding |
| III | 3436724 | * | +G | Non-coding |
| III | 3442521 | * | -C | Non-coding |
| III | 3463329 | c | G | Non-coding |
| III | 3466298 | * | +A | Non-coding |
| III | 3466490 | * | +A | Non-coding |
| III | 3467137 | t | C | exonic (*pph-6*) |
| III | 3467466 | c | T | exonic (*pph-6*) |
| III | 3468663 | a | C | Non-coding |
| III | 3474632 | * | +C | Non-coding |
| III | 3511694 | * | -A | Non-coding |
| III | 3540490 | * | -C | Non-coding |
| III | 3594902 | * | +C | Non-coding |
| III | 3599953 | * | +C | Non-coding |
| III | 3611202 | c | A | Non-coding |
| III | 3613757 | * | +T | Non-coding |
| III | 3613833 | g | T | Non-coding |
| III | 3615997 | c | T | Non-coding |
| III | 3616756 | c | T | Non-coding |
| III | 3625546 | * | +A | Non-coding |
| III | 3625872 | t | A | Non-coding |
| III | 3640946 | * | +G | Non-coding |
| III | 3641475 | g | T | Non-coding |
| III | 3641888 | g | A | Non-coding |
| III | 3645769 | c | A | Non-coding |
| III | 3645821 | t | G | Non-coding |
| III | 3664405 | * | +G | exonic (C46F11.4) |
| III | 3715842 | * | -T | Non-coding |
| III | 3716562 | * | +C | Non-coding |
| III | 3786830 | * | +TC | Non-coding |
| III | 3805250 | * | -C | Non-coding |
| III | 3830219 | * | +G | Non-coding |
| III | 3837182 | * | -T | Non-coding |
| III | 3867246 | * | +A | Non-coding |
| III | 3885443 | * | +G | Non-coding |
| III | 3990963 | * | -C | Non-coding |
| III | 3992712 | * | +CC | Non-coding |
| III | 3995391 | * | -T | Non-coding |
| III | 3995592 | * | +G | Non-coding |
| III | 4001629 | * | +C | Non-coding |
| III | 4014794 | * | +T | Non-coding |
| III | 4019053 | * | +C | Non-coding |
| III | 4019157 | * | +G | Non-coding |
| III | 4032835 | * | +C | Non-coding |
| III | 4048562 | * | +G | Non-coding |
| III | 4059720 | * | -T | exonic (*his-70*) |
| III | 4091847 | * | +C | Non-coding |
| III | 4209222 | a | C | Non-coding |
| III | 4209244 | g | C | Non-coding |
| III | 4210818 | * | +G | Non-coding |
| III | 4340348 | * | +T | exonic (B0285.1) |
| III | 4347357 | * | +A | Non-coding |
| III | 4428989 | * | +C | Non-coding |
| III | 4439724 | * | +C | Non-coding |
| III | 4440871 | t | A | Non-coding |
| III | 4445014 | * | +G | Non-coding |
| III | 4447067 | * | +G | Non-coding |
| III | 4447640 | * | +C | Non-coding |
| III | 4502804 | * | +G | Non-coding |
| III | 4530452 | * | -A | Non-coding |
| III | 4604999 | * | -T | Non-coding |
| III | 4612782 | * | +C | Non-coding |
| III | 4628542 | * | +G | exonic (clec-151) |
| III | 4640495 | * | +G | exonic (clec-154) |
| III | 4684391 | * | -G | Non-coding |
| III | 4687672 | * | +C | Non-coding |
| III | 4689555 | * | +C | Non-coding |
| III | 4708948 | * | +C | Non-coding |
| III | 4808803 | * | +C | Non-coding |
| III | 4938807 | * | +T | Non-coding |
| III | 4947902 | * | +C | Non-coding |
| III | 4962071 | * | -T | Non-coding |
| III | 4969629 | * | -G | Non-coding |
| III | 4973155 | * | -G | Non-coding |
| III | 4983226 | * | -A | Non-coding |
| III | 4986886 | * | +GC | exonic (C27F2.10) |
| III | 5017347 | * | +C | Non-coding |
| III | 5030204 | * | -G | Non-coding |
| III | 5032430 | * | +G | Non-coding |
| III | 5048528 | * | +T | Non-coding |
| III | 5053721 | * | +G | Non-coding |
| III | 5066884 | * | +G | Non-coding |
| III | 5084872 | * | +G | Non-coding |
| III | 5105424 | * | +C | Non-coding |
| III | 5105938 | * | +G | exonic (F54D8.6) |
| III | 5333259 | * | +C | exonic (F52C9.1) |
| III | 5416422 | * | +T | Non-coding |
| III | 5443179 | * | +G | Non-coding |
| III | 5467690 | * | -A | exonic (F48E8.2) |
| III | 5468260 | * | -A | Non-coding |
| III | 5471937 | * | +C | Non-coding |
| III | 5512820 | * | +G | Non-coding |
| III | 5531117 | * | +G | Non-coding |
| III | 5577371 | * | +G | Non-coding |
| III | 5595877 | * | +G | exonic (*ced-6*) |
| III | 5644382 | * | +C | Non-coding |
| III | 5647468 | * | +T | Non-coding |
| III | 5647754 | * | +C | Non-coding |
| III | 5665836 | * | +G | Non-coding |
| III | 5695322 | * | +C | Non-coding |
| III | 5699102 | * | -G | Non-coding |
| III | 5722043 | * | +C | Non-coding |
| III | 5761574 | * | +G | Non-coding |
| III | 5762033 | * | +C | Non-coding |
| III | 5765138 | * | -C | Non-coding |
| III | 5766657 | * | -C | Non-coding |
| III | 5814708 | * | -G | Non-coding |
| III | 5872626 | * | +T | Non-coding |
| III | 5889982 | * | +A | Non-coding |
| III | 5937104 | * | +C | Non-coding |
| III | 5938647 | * | +C | Non-coding |
| III | 5950893 | * | +G | Non-coding |
| III | 5954668 | * | +T | Non-coding |
| III | 5958295 | * | +C | Non-coding |
| III | 5969452 | * | -G | Non-coding |
| III | 5976634 | * | +G | Non-coding |
| III | 5987892 | * | -T | Non-coding |
| III | 6033357 | * | -A | exonic (ZK328.7) |
| III | 6033561 | * | +C | Non-coding |
| III | 6046998 | * | +G | Non-coding |
| III | 6101998 | * | +C | Non-coding |
| III | 6193200 | * | +C | Non-coding |
| III | 6209705 | * | +C | Non-coding |
| III | 6280050 | * | -A | Non-coding |
| III | 6300173 | * | -G | Non-coding |
| III | 6304028 | * | +C | Non-coding |
| III | 6305027 | * | +T | Non-coding |
| III | 6322253 | * | -C | exonic (C56G2.5) |
| III | 6324039 | * | -C | Non-coding |
| III | 6331619 | * | +C | Non-coding |
| III | 6354328 | * | +T | Non-coding |
| III | 6399860 | * | +A | Non-coding |
| III | 6428922 | * | -G | Non-coding |
| III | 6479569 | * | +C | Non-coding |
| III | 6503236 | * | +C | Non-coding |
| III | 6685074 | * | +T | Non-coding |
| III | 6701836 | * | +CC | Non-coding |
| III | 6710989 | * | +C | Non-coding |
| III | 6711783 | * | +C | Non-coding |
| III | 6735539 | * | -A | Non-coding |
| III | 6735690 | * | -T | Non-coding |
| III | 6757603 | * | +C | Non-coding |
| III | 6798013 | * | +CG | Non-coding |
| III | 6813206 | * | +T | Non-coding |
| III | 6852002 | * | -A | Non-coding |
| III | 6888302 | * | +G | Non-coding |
| III | 6925971 | * | +T | Non-coding |
| III | 6950492 | * | +T | Non-coding |
| III | 6968407 | * | -A | Non-coding |
| III | 6985561 | * | +A | Non-coding |
| III | 6987799 | * | -G | Non-coding |
| III | 6990359 | * | +G | Non-coding |
| III | 7013025 | * | -G | Non-coding |
| III | 7021097 | * | +G | Non-coding |
| III | 7028558 | * | -G | Non-coding |
| III | 7029618 | * | +A | Non-coding |
| III | 7030412 | * | -G | Non-coding |
| III | 7032061 | * | +C | Non-coding |
| III | 7037653 | * | +C | Non-coding |
| III | 7047284 | * | +C | Non-coding |
| III | 7075892 | * | +C | exonic (ZK418.6) |
| III | 7079764 | * | +C | Non-coding |
| III | 7144162 | * | +A | Non-coding |
| III | 7236658 | * | -C | Non-coding |
| III | 7253849 | * | +C | Non-coding |
| III | 7272183 | * | +G | Non-coding |
| III | 7353081 | * | +T | Non-coding |
| III | 7357294 | * | +C | exonic (F08F8.10) |
| III | 7388172 | * | +T | Non-coding |
| III | 7389288 | * | +T | Non-coding |
| III | 7453283 | * | -TT | Non-coding |
| III | 7494923 | * | +C | Non-coding |
| III | 7528176 | * | -C | Non-coding |
| III | 7539938 | * | +T | Non-coding |
| III | 7558821 | * | +G | Non-coding |
| III | 7587272 | * | -G | Non-coding |
| III | 7587396 | * | -G | Non-coding |
| III | 7587557 | * | -G | Non-coding |
| III | 7587843 | * | -C | Non-coding |
| III | 7588539 | * | -C | Non-coding |
| III | 7588664 | * | -A | Non-coding |
| III | 7608528 | * | +C | exonic (T04A6.3) |
| III | 7643501 | * | -T | Non-coding |
| III | 7643564 | * | +T | Non-coding |
| III | 7646017 | * | +C | Non-coding |
| III | 7711005 | * | -G | Non-coding |
| III | 7712934 | * | +G | Non-coding |
| III | 7755526 | * | +GC | Non-coding |
| III | 7822735 | * | +G | Non-coding |
| III | 7850551 | * | +C | Non-coding |
| III | 7864174 | * | -C | Non-coding |
| III | 7874571 | * | -C | Non-coding |
| III | 7882070 | * | -G | Non-coding |
| III | 8029613 | * | +A | Non-coding |
| III | 8042706 | * | +G | exonic (K12H4.6) |
| III | 8053798 | * | -C | Non-coding |
| III | 8068403 | * | +G | exonic (*ceh-26*) |
| III | 8070698 | * | +G | Non-coding |
| III | 8130858 | * | +C | exonic (C14B9.10) |
| III | 8147228 | * | -G | Non-coding |
| III | 8181536 | * | +C | Non-coding |
| III | 8185616 | * | +A | Non-coding |
| III | 8191059 | * | +G | Non-coding |
| III | 8194455 | * | +G | Non-coding |
| III | 8201331 | * | +C | Non-coding |
| III | 8309463 | * | +A | Non-coding |
| III | 8335263 | * | +G | Non-coding |
| III | 8423181 | * | +A | Non-coding |
| III | 8423448 | * | +C | Non-coding |
| III | 8438265 | * | +C | exonic (*pqn-96*) |
| III | 8454126 | * | +C | Non-coding |
| III | 8456621 | * | +G | Non-coding |
| III | 8461760 | * | +G | Non-coding |
| III | 8463885 | * | +C | Non-coding |
| III | 8466855 | * | +CT | Non-coding |
| III | 8467144 | * | +G | Non-coding |
| III | 8472515 | * | +C | Non-coding |
| III | 8479958 | * | -A | Non-coding |
| III | 8493382 | * | +CG | Non-coding |
| III | 8511282 | * | +C | Non-coding |
| III | 8512792 | * | +C | Non-coding |
| III | 8520706 | * | +GC | Non-coding |
| III | 8548465 | * | +C | Non-coding |
| III | 8552915 | * | +G | exonic (*gsto-2*) |
| III | 8563845 | * | +A | Non-coding |
| III | 8563896 | * | -A | Non-coding |
| III | 8570399 | * | +C | exonic (CE7X_3.1) |
| III | 8574371 | * | -C | Non-coding |
| III | 8575613 | * | -C | Non-coding |
| III | 8583355 | * | +T | Non-coding |
| III | 8650752 | * | +G | exonic (*flp-23*) |
| III | 8684262 | * | -A | Non-coding |
| III | 8693440 | * | +A | Non-coding |
| III | 8693544 | * | -T | Non-coding |
| III | 8693687 | * | -A | Non-coding |
| III | 8693744 | * | -G | Non-coding |
| III | 8705716 | * | +T | Non-coding |
| III | 8706589 | * | +AC | exonic (B0303.11) |
| III | 8710710 | * | +G | Non-coding |
| III | 8711496 | * | +T | Non-coding |
| III | 8711856 | * | -C | Non-coding |
| III | 8712578 | * | +T | Non-coding |
| III | 8713028 | * | -A | Non-coding |
| III | 8741582 | * | -G | Non-coding |
| III | 8755806 | * | +G | Non-coding |
| III | 8884091 | * | -T | Non-coding |
| III | 8888181 | * | +G | Non-coding |
| III | 8890776 | * | -G | Non-coding |
| III | 8905207 | * | -C | Non-coding |
| III | 8910472 | * | +A | Non-coding |
| III | 8915888 | * | -T | Non-coding |
| III | 8921083 | * | +T | Non-coding |
| III | 8929471 | * | -A | Non-coding |
| III | 8976440 | * | -T | exonic (pde-2) |
| III | 9021444 | * | +C | Non-coding |
| III | 9022411 | * | +T | Non-coding |
| III | 9026032 | * | -C | Non-coding |
| III | 9037626 | * | -AA | Non-coding |
| III | 9059036 | * | -T | Non-coding |
| III | 9066057 | c | G | Non-coding |
| III | 9101167 | g | T | Nonsynonymous (ZK507.1) |
| III | 9111890 | * | -AAAT | Non-coding |
| III | 9150075 | * | +A | Non-coding |
| III | 9199646 | * | +A | Non-coding |
| III | 9221006 | * | +T | Non-coding |
| III | 9260713 | * | -G | Non-coding |
| III | 9266377 | * | +C | Non-coding |
| III | 9281090 | * | +C | Non-coding |
| III | 9281974 | * | +G | Non-coding |
| III | 9282312 | * | +C | Non-coding |
| III | 9291252 | * | -G | Non-coding |
| III | 9295738 | * | +T | Non-coding |
| III | 9343557 | * | +A | Non-coding |
| III | 9348779 | * | +G | Non-coding |
| III | 9350788 | * | +C | Non-coding |
| III | 9357228 | * | +C | Non-coding |
| III | 9361622 | * | -A | Non-coding |
| III | 9365339 | * | -G | Non-coding |
| III | 9411375 | * | +C | Non-coding |
| III | 9447123 | * | -A | Non-coding |
| III | 9468478 | * | +T | Non-coding |
| III | 9547169 | * | +T | Non-coding |
| III | 9571113 | * | -C | Non-coding |
| III | 9583694 | * | -C | Non-coding |
| III | 9590113 | * | -G | Non-coding |
| III | 9590351 | * | +G | Non-coding |
| III | 9637546 | * | -A | Non-coding |
| III | 9639397 | * | +G | Non-coding |
| III | 9642177 | * | +C | Non-coding |
| III | 9647637 | * | -A | Non-coding |
| III | 9728788 | * | +G | Non-coding |
| III | 9765556 | * | +C | Non-coding |
| III | 9783614 | * | +C | Non-coding |
| III | 9785903 | * | -T | exonic (R10E11.5) |
| III | 9793256 | * | +C | Non-coding |
| III | 9795572 | * | +G | Non-coding |
| III | 9853803 | * | +G | Non-coding |
| III | 9858891 | * | +G | Non-coding |
| III | 9860004 | * | -G | Non-coding |
| III | 9870318 | * | +C | Non-coding |
| III | 9879384 | * | +G | Non-coding |
| III | 9907572 | * | +G | Non-coding |
| III | 9908289 | * | -G | Non-coding |
| III | 9910257 | * | +A | Non-coding |
| III | 9920825 | * | -C | Non-coding |
| III | 9922532 | * | -G | Non-coding |
| III | 9962557 | * | +C | Non-coding |
| III | 9971897 | * | -TT | Non-coding |
| III | 10004047 | * | +G | Non-coding |
| III | 10019612 | * | -C | Non-coding |
| III | 10027250 | * | -G | Non-coding |
| III | 10030883 | * | +G | exonic (M04D8.4) |
| III | 10031246 | * | -TG | Non-coding |
| III | 10032181 | * | +T | Non-coding |
| III | 10033776 | * | +T | Non-coding |
| III | 10034772 | * | +T | Non-coding |
| III | 10061336 | * | +C | Non-coding |
| III | 10100792 | * | +GC | Non-coding |
| III | 10100827 | * | +G | Non-coding |
| III | 10100858 | * | +CC | Non-coding |
| III | 10101065 | * | +CGGCGG | Non-coding |
| III | 10131666 | * | -C | Non-coding |
| III | 10147956 | * | +G | Non-coding |
| III | 10213729 | * | +GA | Non-coding |
| III | 10282657 | * | +G | Non-coding |
| III | 10289003 | * | +C | Non-coding |
| III | 10289292 | * | +G | Non-coding |
| III | 10291743 | * | -AC | Non-coding |
| III | 10294797 | * | +G | Non-coding |
| III | 10320209 | * | -G | Non-coding |
| III | 10359134 | * | +CG | Non-coding |
| III | 10368847 | * | +GAC | Non-coding |
| III | 10371021 | * | -A | Non-coding |
| III | 10377649 | * | +C | Non-coding |
| III | 10377684 | * | +T | Non-coding |
| III | 10378482 | * | +CG | Non-coding |
| III | 10381436 | * | +T | Non-coding |
| III | 10382034 | * | +C | Non-coding |
| III | 10385217 | * | -C | Non-coding |
| III | 10388877 | * | -A | Non-coding |
| III | 10397840 | * | +G | Non-coding |
| III | 10402278 | * | -T | Non-coding |
| III | 10407463 | * | +G | exonic (*tag-235*) |
| III | 10407508 | * | +C | exonic (*tag-235*) |
| III | 10428176 | * | +G | Non-coding |
| III | 10460402 | * | +G | Non-coding |
| III | 10483638 | * | -C | exonic (D2045.8) |
| III | 10499629 | * | +C | Non-coding |
| III | 10503104 | * | +A | Non-coding |
| III | 10540094 | * | +G | Non-coding |
| III | 11318803 | * | +AA | Non-coding |
| III | 11326567 | * | +T | exonic (T28D6.6) |
| IV | 149978 | a | G | Non-coding |
| IV | 495583 | t | G | Non-coding |
| IV | 743294 | a | G | Non-coding |
| IV | 757658 | t | A | Nonsynonymous (*srt-23*) |
| IV | 807998 | t | C | Non-coding |
| IV | 976182 | t | C | Non-coding |
| IV | 976984 | a | G | Non-coding |
| IV | 991371 | g | C | Non-coding |
| IV | 1197597 | g | T | Non-coding |
| IV | 1201163 | * | +GC | Non-coding |
| IV | 1336744 | * | +A | Non-coding |
| IV | 1351820 | * | +G | Non-coding |
| IV | 1386027 | a | G | Non-coding |
| IV | 1438459 | a | T | Synonymous (*clp-7*) |
| IV | 1477954 | * | +A | Non-coding |
| IV | 1480524 | * | +C | Non-coding |
| IV | 1515189 | * | +G | exonic (K03H6.2) |
| IV | 1695528 | g | C | Non-coding |
| IV | 1770279 | * | -A | Non-coding |
| IV | 1837526 | * | +A | Non-coding |
| IV | 1864649 | * | -T | Non-coding |
| IV | 1972674 | * | -G | Non-coding |
| IV | 2130505 | a | T | Non-coding |
| IV | 2226862 | t | C | Non-coding |
| IV | 2357212 | g | T | Non-coding |
| IV | 2359440 | a | G | Non-coding |
| IV | 2363295 | * | +TTT | Non-coding |
| IV | 2365375 | * | +T | Non-coding |
| IV | 2373373 | t | C | Non-coding |
| IV | 2541640 | * | +A | Non-coding |
| IV | 2614793 | * | +A | Non-coding |
| IV | 2672326 | * | -CGG | Non-coding |
| IV | 2734649 | * | -CT | Non-coding |
| IV | 2999123 | a | G | Synonymous (Y54G2A.27) |
| IV | 3036892 | * | -GAATTC | Non-coding |
| IV | 3057323 | * | +A | Non-coding |
| IV | 3098667 | * | +T | Non-coding |
| IV | 3124403 | * | -A | Non-coding |
| IV | 3143653 | * | +A | Non-coding |
| IV | 3162657 | * | +T | Non-coding |
| IV | 3382092 | * | -T | exonic (B0546.3) |
| IV | 3524137 | * | +G | Non-coding |
| IV | 3525903 | t | A | Non-coding |
| IV | 3570819 | * | +G | Non-coding |
| IV | 3636463 | * | +CA | Non-coding |
| IV | 3791266 | * | +A | Non-coding |
| IV | 3824608 | * | +A | Non-coding |
| IV | 3831276 | * | +C | Non-coding |
| IV | 3838405 | * | +C | Non-coding |
| IV | 3864658 | * | +C | Non-coding |
| IV | 3951382 | * | -G | Non-coding |
| IV | 4040393 | * | +G | Non-coding |
| IV | 4166340 | a | T | Non-coding |
| IV | 4583605 | * | +G | Non-coding |
| IV | 4593004 | * | +G | Non-coding |
| IV | 4617778 | c | T | Non-coding |
| IV | 4676115 | * | +G | Non-coding |
| IV | 4695312 | g | T | Non-coding |
| IV | 4801202 | * | +C | Non-coding |
| IV | 4821642 | * | +G | Non-coding |
| IV | 4931163 | a | C | Nonsynonymous (Y9C9A.8) |
| IV | 4972666 | c | T | Nonsynonymous (*srz-28*) |
| IV | 5111681 | * | +A | Non-coding |
| IV | 5208043 | * | +T | exonic (Y59E9AL.4) |
| IV | 5308471 | a | C | Non-coding |
| IV | 5405344 | * | +T | exonic (ZK616.1) |
| IV | 5409611 | * | +G | exonic (ZK616.8) |
| IV | 5426794 | g | T | Non-coding |
| IV | 5481593 | * | +A | Non-coding |
| IV | 5516506 | * | +C | Non-coding |
| IV | 5529112 | * | -A | Non-coding |
| IV | 5582581 | * | +C | Non-coding |
| IV | 5587984 | * | +A | Non-coding |
| IV | 5588033 | * | +T | Non-coding |
| IV | 5609145 | * | +A | Non-coding |
| IV | 5706864 | * | +T | Non-coding |
| IV | 5794582 | * | -G | Non-coding |
| IV | 5809277 | * | +T | Non-coding |
| IV | 5884811 | * | +T | Non-coding |
| IV | 6101893 | * | -A | Non-coding |
| IV | 6646382 | g | C | Non-coding |
| IV | 7056612 | a | G | Non-coding |
| IV | 7079566 | * | +GC | Non-coding |
| IV | 7111783 | * | +G | Non-coding |
| IV | 7226680 | * | -C | Non-coding |
| IV | 7382031 | * | +T | Non-coding |
| IV | 7600485 | a | T | Nonsynonymous (*tag-80*) |
| IV | 7727542 | * | +G | exonic (C50F7.5) |
| IV | 7897217 | * | +C | Non-coding |
| IV | 8007299 | * | +G | Non-coding |
| IV | 8015831 | g | C | Non-coding |
| IV | 8015865 | a | C | Non-coding |
| IV | 8061990 | * | +C | Non-coding |
| IV | 8087150 | * | -GATC | Non-coding |
| IV | 8271544 | * | -T | exonic (K07H8.5) |
| IV | 8468403 | c | T | Non-coding |
| IV | 8521446 | a | T | Non-coding |
| IV | 8602902 | c | A | Non-coding |
| IV | 8602934 | c | A | Non-coding |
| IV | 8632715 | * | +A | exonic (*dgk-4*) |
| IV | 8946713 | * | -C | Non-coding |
| IV | 8949001 | * | +A | Non-coding |
| IV | 8952091 | * | +C | Non-coding |
| IV | 8966627 | * | +A | Non-coding |
| IV | 9038439 | g | A | Non-coding |
| IV | 9048788 | c | G | Non-coding |
| IV | 9048807 | c | G | Non-coding |
| IV | 9048812 | t | A | Non-coding |
| IV | 9114839 | * | -A | Non-coding |
| IV | 9155016 | * | +T | Non-coding |
| IV | 9187483 | * | +G | Non-coding |
| IV | 9251676 | * | +T | Non-coding |
| IV | 9269038 | t | C | Non-coding |
| IV | 9370614 | * | +G | Non-coding |
| IV | 9524145 | * | +G | Non-coding |
| IV | 9538088 | * | +A | Non-coding |
| IV | 9715772 | * | +A | Non-coding |
| IV | 9717381 | * | +T | Non-coding |
| IV | 9745669 | g | A | Nonsynonymous (C28D4.5) |
| IV | 9755286 | * | +T | Non-coding |
| IV | 9764970 | * | -G | Non-coding |
| IV | 9840851 | * | +C | exonic (*kin-24*) |
| IV | 9868740 | * | +A | Non-coding |
| IV | 9938179 | g | A | Non-coding |
| IV | 10112494 | * | +C | Non-coding |
| IV | 10329613 | * | -G | Non-coding |
| IV | 10409486 | * | -C | Non-coding |
| IV | 10414829 | * | +G | exonic (F13B12.6) |
| IV | 10425275 | * | -G | Non-coding |
| IV | 10425371 | * | +G | Non-coding |
| IV | 10793215 | g | A | Nonsynonymous (*opt-1*) |
| IV | 10848627 | * | +C | Non-coding |
| IV | 10878679 | g | A | Nonsynonymous (*sdz-13*) |
| IV | 10894240 | * | +C | exonic (F13E9.8) |
| IV | 11009137 | g | A | Non-coding |
| IV | 11050307 | * | -A | exonic (F36H1.3) |
| IV | 11186541 | * | +G | Non-coding |
| IV | 11323985 | * | +G | exonic (*his-47*) |
| IV | 11387891 | * | -A | Non-coding |
| IV | 11493605 | * | +C | Non-coding |
| IV | 11551950 | * | +G | Non-coding |
| IV | 12021851 | * | -A | Non-coding |
| IV | 12091257 | * | +T | Non-coding |
| IV | 12334255 | c | A | Non-coding |
| IV | 12335310 | * | +G | Non-coding |
| IV | 12336927 | * | -A | Non-coding |
| IV | 12341067 | * | +A | Non-coding |
| IV | 12341151 | * | +T | Non-coding |
| IV | 12393299 | * | -G | Non-coding |
| IV | 12416345 | * | -C | Non-coding |
| IV | 12433584 | * | -T | Non-coding |
| IV | 12451706 | * | -TC | Non-coding |
| IV | 12515334 | * | -T | Non-coding |
| IV | 12538990 | * | +A | Non-coding |
| IV | 12571922 | * | +G | Non-coding |
| IV | 12597642 | * | -T | Non-coding |
| IV | 12771599 | * | +C | Non-coding |
| IV | 12883795 | * | +T | Non-coding |
| IV | 13188308 | * | +G | Non-coding |
| IV | 13203415 | * | -A | Non-coding |
| IV | 13348726 | * | +C | Non-coding |
| IV | 13449999 | * | +A | Non-coding |
| IV | 13460708 | * | -G | Non-coding |
| IV | 13490388 | * | +C | Non-coding |
| IV | 13493269 | * | +A | Non-coding |
| IV | 13506420 | * | -A | Non-coding |
| IV | 13695678 | g | C | Non-coding |
| IV | 13764377 | * | +A | Non-coding |
| IV | 13764413 | * | -T | Non-coding |
| IV | 13783663 | * | +C | Non-coding |
| IV | 13790999 | * | -C | exonic (Y45F10D.7) |
| IV | 13793488 | * | -A | Non-coding |
| IV | 14268413 | * | +T | Non-coding |
| IV | 14283221 | * | +A | Non-coding |
| IV | 14298716 | * | -A | Non-coding |
| IV | 14313677 | * | +G | Non-coding |
| IV | 14547466 | * | +C | Non-coding |
| IV | 14640951 | * | +T | Non-coding |
| IV | 14662594 | * | +A | Non-coding |
| IV | 14670517 | * | +G | Non-coding |
| IV | 14707359 | * | +T | Non-coding |
| IV | 14816562 | * | -A | Non-coding |
| IV | 15060615 | a | T | Non-coding |
| IV | 15120234 | * | +CTTCTTCTT | Non-coding |
| IV | 15611206 | * | +C | Non-coding |
| IV | 15798017 | * | +G | Non-coding |
| IV | 15965556 | * | +G | exonic (Y105C5B.18) |
| IV | 16085506 | * | -T | Non-coding |
| IV | 16394027 | * | +AT | Non-coding |
| IV | 16469153 | * | +C | Non-coding |
| IV | 16619776 | * | +A | Non-coding |
| IV | 16747773 | * | -A | Non-coding |
| IV | 16899329 | * | +T | Non-coding |
| IV | 16935084 | * | +C | Non-coding |
| IV | 16961476 | * | +C | Non-coding |
| IV | 17102553 | * | +G | Non-coding |
| IV | 17228761 | * | +C | exonic (Y116A8B.5) |
| IV | 17479087 | * | -T | Non-coding |
| V | 705 | c | T | Non-coding |
| V | 721 | c | G | Non-coding |
| V | 46068 | g | C | Non-coding |
| V | 46069 | t | G | Non-coding |
| V | 46079 | c | A | Non-coding |
| V | 46135 | c | G | Non-coding |
| V | 166555 | * | -T | Non-coding |
| V | 198833 | a | T | Non-coding |
| V | 306925 | g | A | Non-coding |
| V | 351338 | * | +T | Non-coding |
| V | 361876 | a | G | Non-coding |
| V | 839128 | * | +T | Non-coding |
| V | 907030 | c | T | Non-coding |
| V | 931549 | * | -GATC | Non-coding |
| V | 1185763 | * | -G | Non-coding |
| V | 1200282 | * | +GC | Non-coding |
| V | 1211229 | * | +A | Non-coding |
| V | 1273400 | c | G | Non-coding |
| V | 1273408 | c | G | Non-coding |
| V | 1275708 | * | +G | Non-coding |
| V | 1289272 | g | A | Non-coding |
| V | 1506801 | * | +T | Non-coding |
| V | 1777075 | * | +T | Non-coding |
| V | 2019954 | * | +GAA | Non-coding |
| V | 2255011 | g | A | Non-coding |
| V | 2578146 | g | C | Non-coding |
| V | 2586589 | * | +T | Non-coding |
| V | 2606201 | * | +A | Non-coding |
| V | 3017813 | a | G | Non-coding |
| V | 3276304 | * | +C | Non-coding |
| V | 3556386 | * | +C | Non-coding |
| V | 3587028 | * | +AGGATCCTTC | Non-coding |
| V | 3627696 | * | +A | Non-coding |
| V | 3648001 | t | A | Non-coding |
| V | 3750651 | * | +T | exonic (Y39H10A.6) |
| V | 3811972 | * | +G | Non-coding |
| V | 4030988 | * | -GG | Non-coding |
| V | 4139270 | * | -C | Non-coding |
| V | 4164869 | a | C | Nonsynonymous (Y45G5AM.3) |
| V | 4296909 | * | +A | Non-coding |
| V | 4321383 | * | +A | Non-coding |
| V | 4329455 | a | C | Non-coding |
| V | 4343578 | a | C | Non-coding |
| V | 4351932 | * | +G | Non-coding |
| V | 4391657 | * | +A | Non-coding |
| V | 4447836 | c | G | Non-coding |
| V | 4597289 | a | G | Non-coding |
| V | 4704362 | * | +A | Non-coding |
| V | 4770332 | * | +CC | Non-coding |
| V | 4988947 | * | +A | Non-coding |
| V | 5070085 | * | +A | Non-coding |
| V | 5124308 | t | C | Non-coding |
| V | 5124714 | t | A | Non-coding |
| V | 5162299 | * | +G | Non-coding |
| V | 5689586 | * | -A | Non-coding |
| V | 5756335 | * | +A | Non-coding |
| V | 5845363 | * | +C | Non-coding |
| V | 6036730 | * | +GC | Non-coding |
| V | 6040933 | * | +C | Non-coding |
| V | 6048318 | c | A | Non-coding |
| V | 6052074 | t | C | Non-coding |
| V | 6177079 | c | A | Non-coding |
| V | 6177083 | t | G | Non-coding |
| V | 6302463 | a | G | Non-coding |
| V | 6578907 | * | -A | Non-coding |
| V | 6781537 | * | +T | Non-coding |
| V | 6865466 | * | +G | Non-coding |
| V | 6873249 | * | +C | Non-coding |
| V | 6889636 | c | G | Synonymous (K11C4.3) |
| V | 6889637 | g | C | Nonsynonymous (*unc-70*) |
| V | 6896896 | * | +T | Non-coding |
| V | 6912839 | * | -A | Non-coding |
| V | 6956711 | t | C | Nonsynonymous (*mec-1*) |
| V | 6956743 | c | G | Nonsynonymous (*mec-1*) |
| V | 6956744 | g | C | Nonsynonymous (*mec-1*) |
| V | 6972719 | * | +T | Non-coding |
| V | 7108979 | * | +C | Non-coding |
| V | 7205625 | * | -G | exonic (C13F10.4) |
| V | 7245105 | c | G | Non-coding |
| V | 7386791 | * | +A | Non-coding |
| V | 7546600 | g | A | Nonsynonymous (*ftn-1*) |
| V | 7725857 | * | -GATC | Non-coding |
| V | 7817732 | * | +A | Non-coding |
| V | 7860214 | c | A | Non-coding |
| V | 7860248 | a | T | Non-coding |
| V | 7891928 | * | +A | Non-coding |
| V | 8056170 | g | T | Non-coding |
| V | 8233594 | * | +C | Non-coding |
| V | 8408274 | * | +T | Non-coding |
| V | 8425798 | * | +G | Non-coding |
| V | 8533786 | * | +C | Non-coding |
| V | 8619234 | * | +GCTA | Non-coding |
| V | 8751075 | * | -G | Non-coding |
| V | 8757751 | * | +T | Non-coding |
| V | 8792708 | * | +A | exonic (*tag-117*) |
| V | 8924261 | t | A | Non-coding |
| V | 8969821 | * | +A | Non-coding |
| V | 9007887 | * | -T | Non-coding |
| V | 9063305 | * | -GATCG | Non-coding |
| V | 9113930 | * | +G | Non-coding |
| V | 9155855 | * | +A | Non-coding |
| V | 9162494 | t | G | Nonsynonymous (*col-145*) |
| V | 9290037 | * | +C | Non-coding |
| V | 9294259 | * | -T | Non-coding |
| V | 9309037 | * | -T | Non-coding |
| V | 9339747 | * | +C | Non-coding |
| V | 9376379 | g | T | Nonsynonymous (E02C12.10) |
| V | 9537615 | a | C | Non-coding |
| V | 9603798 | a | G | Non-coding |
| V | 9662867 | t | C | Non-coding |
| V | 9663159 | t | G | Non-coding |
| V | 9669523 | * | +C | Non-coding |
| V | 9698443 | * | +C | Non-coding |
| V | 9707449 | t | C | Non-coding |
| V | 9746724 | * | +G | Non-coding |
| V | 9750477 | * | +A | Non-coding |
| V | 9750974 | * | -C | Non-coding |
| V | 9927293 | t | A | Non-coding |
| V | 9928614 | c | T | Nonsynonymous (T07C12.12) |
| V | 10067797 | * | +C | Non-coding |
| V | 10164059 | * | +G | Non-coding |
| V | 10230413 | * | +G | Non-coding |
| V | 10235806 | * | -A | Non-coding |
| V | 10245639 | * | +T | Non-coding |
| V | 10336187 | * | -A | Non-coding |
| V | 10336307 | * | -T | Non-coding |
| V | 10514263 | * | +G | Non-coding |
| V | 10548751 | * | -G | exonic (*srd-34*) |
| V | 10596517 | * | -C | Non-coding |
| V | 10598994 | * | -C | Non-coding |
| V | 10696345 | * | -A | Non-coding |
| V | 10862975 | * | -T | Non-coding |
| V | 11012491 | * | +G | Non-coding |
| V | 11025874 | * | -T | Non-coding |
| V | 11026612 | * | -A | Non-coding |
| V | 11026652 | * | -T | Non-coding |
| V | 11495158 | * | -T | exonic (T11F9.12) |
| V | 11505117 | * | -A | Non-coding |
| V | 11782174 | * | +G | Non-coding |
| V | 12166926 | * | +C | Non-coding |
| V | 12590428 | * | -A | Non-coding |
| V | 12647437 | * | -A | Non-coding |
| V | 12810474 | * | +T | exonic (ZC443.2) |
| V | 12867713 | * | -A | Non-coding |
| V | 12985358 | * | -A | Non-coding |
| V | 13150205 | * | -G | exonic (B0365.7) |
| V | 13162704 | * | +A | Non-coding |
| V | 13183406 | * | -A | Non-coding |
| V | 13278626 | * | -A | Non-coding |
| V | 13307389 | t | G | Non-coding |
| V | 13418590 | * | +A | Non-coding |
| V | 13506486 | * | -C | Non-coding |
| V | 13506613 | * | +T | exonic (R11G10.4) |
| V | 13524542 | * | +C | Non-coding |
| V | 13539849 | * | -G | Non-coding |
| V | 13665872 | * | +A | exonic (F58G11.2) |
| V | 13706741 | * | +G | Non-coding |
| V | 13720951 | * | -A | Non-coding |
| V | 13774969 | * | +C | Non-coding |
| V | 13958673 | * | +T | Non-coding |
| V | 14250252 | * | +T | Non-coding |
| V | 14524334 | * | +G | Non-coding |
| V | 14524374 | * | +C | Non-coding |
| V | 14930541 | * | +C | Non-coding |
| V | 15440054 | * | +C | Non-coding |
| V | 15527876 | * | +G | Non-coding |
| V | 15657140 | * | -T | Non-coding |
| V | 15688514 | * | +T | Non-coding |
| V | 15740301 | * | +A | Non-coding |
| V | 15756605 | * | -C | Non-coding |
| V | 15935382 | * | +A | Non-coding |
| V | 16093679 | * | +A | Non-coding |
| V | 16420522 | g | A | Non-coding |
| V | 16460133 | * | +A | Non-coding |
| V | 16584111 | * | +C | Non-coding |
| V | 16690157 | * | +T | Non-coding |
| V | 16874735 | * | +G | Non-coding |
| V | 16905154 | * | +T | Non-coding |
| V | 17189488 | * | +A | Non-coding |
| V | 17469459 | * | +A | Non-coding |
| V | 17961403 | * | +A | Non-coding |
| V | 17975949 | * | +A | Non-coding |
| V | 17984730 | * | +T | Non-coding |
| V | 17989159 | * | +C | Non-coding |
| V | 17999782 | * | +C | Non-coding |
| V | 18314060 | * | +C | Non-coding |
| V | 18465992 | g | A | Non-coding |
| V | 18469308 | * | -A | Non-coding |
| V | 18533903 | * | -A | Non-coding |
| V | 18557360 | * | +C | Non-coding |
| V | 18785909 | * | +C | Non-coding |
| V | 19109904 | * | -G | Non-coding |
| V | 19485465 | * | +G | Non-coding |
| V | 19639163 | * | +TTTT | Non-coding |
| V | 19680154 | * | -T | Non-coding |
| X | 5526 | * | -TGAGC | Non-coding |
| X | 5703 | * | +T | Non-coding |
| X | 161175 | g | T | Non-coding |
| X | 172635 | * | +A | Non-coding |
| X | 187274 | c | G | Non-coding |
| X | 289841 | g | C | Non-coding |
| X | 299633 | t | A | Non-coding |
| X | 320952 | * | +G | Non-coding |
| X | 341454 | g | C | Non-coding |
| X | 346995 | g | T | Non-coding |
| X | 348115 | c | A | Non-coding |
| X | 351367 | * | +G | Non-coding |
| X | 365023 | * | -T | Non-coding |
| X | 379724 | * | +C | Non-coding |
| X | 381258 | * | +TC | Non-coding |
| X | 382890 | c | G | Non-coding |
| X | 424545 | t | C | Non-coding |
| X | 424576 | t | C | Non-coding |
| X | 424587 | t | C | Non-coding |
| X | 424629 | t | C | Non-coding |
| X | 424765 | g | T | Non-coding |
| X | 425163 | * | -T | Non-coding |
| X | 425343 | * | -G | Non-coding |
| X | 425402 | * | +T | Non-coding |
| X | 425463 | * | +G | Non-coding |
| X | 425547 | c | G | Non-coding |
| X | 443404 | c | G | Non-coding |
| X | 486102 | a | G | Non-coding |
| X | 488781 | a | C | Non-coding |
| X | 488806 | g | T | Non-coding |
| X | 491245 | * | +C | Non-coding |
| X | 491868 | c | A | Non-coding |
| X | 538147 | * | -C | Non-coding |
| X | 544531 | * | +T | Non-coding |
| X | 576111 | t | A | Non-coding |
| X | 576115 | t | C | Non-coding |
| X | 582138 | * | +G | Non-coding |
| X | 632238 | * | +C | Non-coding |
| X | 817619 | a | T | Non-coding |
| X | 872120 | * | +G | Non-coding |
| X | 928307 | * | +A | Non-coding |
| X | 1017350 | t | A | Synonymous (F55A4.2) |
| X | 1047732 | c | T | Non-coding |
| X | 1058694 | * | +T | Non-coding |
| X | 1073732 | g | T | Non-coding |
| X | 1133524 | t | A | Non-coding |
| X | 1133556 | t | A | Non-coding |
| X | 1175648 | t | C | Non-coding |
| X | 1192516 | * | +C | Non-coding |
| X | 1198526 | * | +G | Non-coding |
| X | 1201506 | * | -A | Non-coding |
| X | 1202975 | * | +G | exonic (T13G4.6) |
| X | 1226077 | g | C | Non-coding |
| X | 1239148 | c | T | Non-coding |
| X | 1308534 | c | A | Non-coding |
| X | 1333863 | a | C | Non-coding |
| X | 1397899 | * | +CG | Non-coding |
| X | 1472305 | g | T | Non-coding |
| X | 1509924 | * | -CCTTG | Non-coding |
| X | 1519786 | c | A | Non-coding |
| X | 1519787 | c | A | Non-coding |
| X | 1604340 | g | T | Non-coding |
| X | 1638560 | a | G | Non-coding |
| X | 1644407 | g | A | Non-coding |
| X | 1644408 | a | G | Non-coding |
| X | 1645681 | * | +T | Non-coding |
| X | 1717742 | c | A | Non-coding |
| X | 1802915 | * | +G | Non-coding |
| X | 2002620 | * | -GAATTC | Non-coding |
| X | 2078712 | * | +T | Non-coding |
| X | 2165878 | * | +G | exonic (F48B9.3) |
| X | 2212358 | * | -C | Non-coding |
| X | 2219010 | * | +G | Non-coding |
| X | 2370396 | * | -A | Non-coding |
| X | 2391699 | * | +T | Non-coding |
| X | 2427198 | * | +G | Non-coding |
| X | 2428242 | * | -T | Non-coding |
| X | 2435478 | * | +C | Non-coding |
| X | 2522231 | * | -C | Non-coding |
| X | 2524915 | t | G | Non-coding |
| X | 2537528 | * | +A | Non-coding |
| X | 2598870 | * | +G | Non-coding |
| X | 2691726 | * | -C | exonic (T14G11.1) |
| X | 2767456 | * | +CGCG | Non-coding |
| X | 2794548 | * | +G | Non-coding |
| X | 2826263 | * | +T | Non-coding |
| X | 2893761 | * | +A | Non-coding |
| X | 2894432 | a | T | Non-coding |
| X | 2899575 | * | +C | Non-coding |
| X | 3027118 | * | -A | Non-coding |
| X | 3027787 | c | G | Non-coding |
| X | 3085538 | * | +T | Non-coding |
| X | 3207500 | * | -C | Non-coding |
| X | 3214375 | * | +T | Non-coding |
| X | 3218522 | * | +G | Non-coding |
| X | 3218895 | * | +A | Non-coding |
| X | 3220678 | * | -G | Non-coding |
| X | 3237694 | a | G | Nonsynonymous (F40F4.6) |
| X | 3271331 | * | +A | Non-coding |
| X | 3293129 | * | +C | Non-coding |
| X | 3293917 | * | -C | Non-coding |
| X | 3314881 | * | +T | Non-coding |
| X | 3323161 | * | +A | Non-coding |
| X | 3367002 | c | G | Non-coding |
| X | 3367003 | g | C | Non-coding |
| X | 3376415 | * | +G | Non-coding |
| X | 3408399 | t | C | Synonymous (*vit-5*) |
| X | 3412023 | * | -G | Non-coding |
| X | 3413727 | * | +T | Non-coding |
| X | 3413856 | * | +G | exonic (*dhs-27*) |
| X | 3520397 | c | G | Non-coding |
| X | 3653458 | t | C | Non-coding |
| X | 3721241 | * | +C | Non-coding |
| X | 3742367 | * | +G | Non-coding |
| X | 3751244 | * | +C | Non-coding |
| X | 3802908 | * | +C | Non-coding |
| X | 3811358 | * | +C | Non-coding |
| X | 3811996 | t | G | Non-coding |
| X | 3812025 | * | +A | Non-coding |
| X | 3867515 | * | +TG | Non-coding |
| X | 3875643 | * | -A | Non-coding |
| X | 3900886 | * | +C | Non-coding |
| X | 3903048 | t | C | Non-coding |
| X | 3924055 | a | G | Non-coding |
| X | 3974052 | * | +C | Non-coding |
| X | 4017951 | * | +C | Non-coding |
| X | 4025107 | a | G | Synonymous (R02E12.8) |
| X | 4025157 | * | +T | exonic (*acr-10*) |
| X | 4026006 | a | C | Non-coding |
| X | 4086774 | * | +C | Non-coding |
| X | 4241735 | a | C | Non-coding |
| X | 4241827 | t | C | Non-coding |
| X | 4241844 | g | A | Non-coding |
| X | 4242094 | g | A | Non-coding |
| X | 4260432 | * | +G | Non-coding |
| X | 4346150 | * | +G | Non-coding |
| X | 4490688 | * | +G | Non-coding |
| X | 4503826 | * | +T | Non-coding |
| X | 4504875 | * | +C | Non-coding |
| X | 4512910 | * | +G | Non-coding |
| X | 4513922 | c | T | Non-coding |
| X | 4548320 | * | -T | Non-coding |
| X | 4550017 | * | -A | Non-coding |
| X | 4556555 | * | +CT | Non-coding |
| X | 4565291 | * | +C | exonic (*amt-4*) |
| X | 4577119 | * | +G | Non-coding |
| X | 4577179 | a | T | Non-coding |
| X | 4578097 | t | C | Non-coding |
| X | 4603453 | * | -G | exonic (*spat-3*) |
| X | 4662792 | * | -A | exonic (F16H11.2) |
| X | 4662891 | g | A | Nonsynonymous (*nurf-1*) |
| X | 4673998 | * | +T | Non-coding |
| X | 4686586 | * | +G | Non-coding |
| X | 4702538 | * | +G | Non-coding |
| X | 4721075 | * | +G | Non-coding |
| X | 4907912 | * | +A | Non-coding |
| X | 4938556 | * | -GATC | Non-coding |
| X | 4971580 | c | T | Non-coding |
| X | 4971581 | a | C | Non-coding |
| X | 5123322 | t | G | Non-coding |
| X | 5131529 | * | +G | Non-coding |
| X | 5134151 | c | G | Non-coding |
| X | 5134152 | g | C | Non-coding |
| X | 5140286 | * | +GA | Non-coding |
| X | 5140412 | * | +CT | Non-coding |
| X | 5170171 | * | +A | Non-coding |
| X | 5195548 | * | +C | Non-coding |
| X | 5462745 | * | +G | exonic (*ddr-1*) |
| X | 5506742 | * | +C | Non-coding |
| X | 5508054 | * | +C | Non-coding |
| X | 5509588 | * | +G | Non-coding |
| X | 5526942 | * | +C | exonic (*lgx-1*) |
| X | 5590454 | * | +G | exonic (*mec-2*) |
| X | 5599497 | * | +G | Non-coding |
| X | 5819246 | * | +C | Non-coding |
| X | 5857436 | t | G | Non-coding |
| X | 5883992 | c | T | Non-coding |
| X | 6064932 | * | +G | Non-coding |
| X | 6075482 | * | +G | exonic (*ggr-2*) |
| X | 6107174 | t | A | Non-coding |
| X | 6222644 | * | +T | Non-coding |
| X | 6224847 | * | -C | Non-coding |
| X | 6227740 | * | +G | Non-coding |
| X | 6240041 | * | +CG | Non-coding |
| X | 6283375 | * | +G | exonic (T07H6.4) |
| X | 6287365 | t | A | Non-coding |
| X | 6287768 | * | +A | Non-coding |
| X | 6287805 | * | +A | Non-coding |
| X | 6374837 | * | +C | exonic (C03B1.1) |
| X | 6412638 | a | T | Non-coding |
| X | 6606512 | * | +C | Non-coding |
| X | 6606548 | * | +G | Non-coding |
| X | 6661215 | * | +G | Non-coding |
| X | 6687555 | * | +C | Non-coding |
| X | 6792031 | g | C | Nonsynonymous (*asp-3*) |
| X | 6833277 | * | +C | Non-coding |
| X | 6843321 | a | T | Non-coding |
| X | 6917594 | * | +G | Non-coding |
| X | 6949712 | * | +C | Non-coding |
| X | 6988063 | * | +G | Non-coding |
| X | 6990874 | * | +T | Non-coding |
| X | 6992869 | * | -T | Non-coding |
| X | 6999220 | * | +A | Non-coding |
| X | 7037471 | * | +G | Non-coding |
| X | 7068543 | * | +G | Non-coding |
| X | 7077942 | * | +TGTC | Non-coding |
| X | 7086344 | * | -A | Non-coding |
| X | 7117961 | * | +A | Non-coding |
| X | 7139443 | * | -C | exonic (*ist-1*) |
| X | 7145338 | * | +C | exonic (*lam-2*) |
| X | 7224737 | a | T | Non-coding |
| X | 7235009 | * | +G | Non-coding |
| X | 7243164 | * | +G | Non-coding |
| X | 7247377 | c | A | Non-coding |
| X | 7288460 | * | +A | Non-coding |
| X | 7306989 | g | T | Non-coding |
| X | 7310131 | * | +C | Non-coding |
| X | 7411506 | * | -G | Non-coding |
| X | 7436793 | * | +T | exonic (C01C10.2) |
| X | 7464543 | * | +G | Non-coding |
| X | 7471835 | * | +A | Non-coding |
| X | 7507749 | * | +G | Non-coding |
| X | 7527774 | c | T | Non-coding |
| X | 7543426 | * | +A | Non-coding |
| X | 7559950 | * | +C | Non-coding |
| X | 7578504 | * | +G | Non-coding |
| X | 7585675 | * | +T | Non-coding |
| X | 7588883 | t | C | Nonsynonymous (*adt-2*) |
| X | 7638194 | * | +C | Non-coding |
| X | 7644183 | * | +G | Non-coding |
| X | 7649424 | * | +G | Non-coding |
| X | 7671832 | * | +C | Non-coding |
| X | 7689676 | * | +C | Non-coding |
| X | 7719005 | * | +C | Non-coding |
| X | 7729211 | c | T | Synonymous (*vit-1*) |
| X | 7764853 | t | A | Non-coding |
| X | 7771370 | g | A | Non-coding |
| X | 7823439 | * | +T | Non-coding |
| X | 7841127 | * | -G | Non-coding |
| X | 7842241 | * | +G | Non-coding |
| X | 7858594 | * | -G | Non-coding |
| X | 7866244 | * | -A | Non-coding |
| X | 7868653 | * | -A | Non-coding |
| X | 7872981 | c | A | Non-coding |
| X | 7893276 | * | +A | Non-coding |
| X | 7950529 | * | -T | Non-coding |
| X | 7984028 | * | -C | Non-coding |
| X | 7986170 | * | +G | exonic (F45E1.4) |
| X | 8013957 | * | +T | Non-coding |
| X | 8026787 | * | +T | exonic (C34D10.2) |
| X | 8029563 | c | T | Non-coding |
| X | 8033807 | * | +C | exonic (C18A11.6) |
| X | 8033820 | g | C | Synonymous (C18A11.6) |
| X | 8053010 | * | +C | Non-coding |
| X | 8076589 | * | -A | Non-coding |
| X | 8163645 | * | +G | Non-coding |
| X | 8191527 | * | +T | Non-coding |
| X | 8207446 | * | +T | Non-coding |
| X | 8274481 | * | +C | Non-coding |
| X | 8325357 | * | -G | exonic (R09F10.8) |
| X | 8374022 | * | +A | Non-coding |
| X | 8408766 | * | +C | Non-coding |
| X | 8408804 | * | +C | Non-coding |
| X | 8502319 | * | +T | Non-coding |
| X | 8516616 | * | +T | Non-coding |
| X | 8550625 | * | -C | Non-coding |
| X | 8596016 | * | +G | exonic (F18E9.7) |
| X | 8596061 | * | -G | exonic (F18E9.7) |
| X | 8601955 | * | +C | exonic (F18E9.1) |
| X | 8624950 | * | +G | Non-coding |
| X | 8629824 | a | C | Non-coding |
| X | 8630460 | * | -C | Non-coding |
| X | 8639230 | * | +CG | Non-coding |
| X | 8639857 | * | -G | exonic (*stg-2*) |
| X | 8641647 | * | +T | Non-coding |
| X | 8757397 | c | A | Non-coding |
| X | 8779906 | * | +C | Non-coding |
| X | 8869841 | * | +T | Non-coding |
| X | 8900504 | * | +A | Non-coding |
| X | 8928415 | * | +A | Non-coding |
| X | 8941343 | * | -GATC | Non-coding |
| X | 8963611 | * | +CG | Non-coding |
| X | 8984740 | * | +G | Non-coding |
| X | 9078277 | * | +G | Non-coding |
| X | 9137581 | * | +C | Non-coding |
| X | 9171369 | g | T | Non-coding |
| X | 9198469 | * | +G | Non-coding |
| X | 9198534 | * | +G | Non-coding |
| X | 9205410 | * | -C | Non-coding |
| X | 9207819 | * | +A | Non-coding |
| X | 9212879 | * | +G | Non-coding |
| X | 9235230 | * | +C | exonic (*lgc-4*) |
| X | 9301361 | * | +AAT | Non-coding |
| X | 9328171 | * | +A | Non-coding |
| X | 9328748 | * | -C | Non-coding |
| X | 9462102 | t | C | Non-coding |
| X | 9489786 | * | -C | Non-coding |
| X | 9498317 | * | -G | Non-coding |
| X | 9536669 | t | A | Non-coding |
| X | 9668896 | * | +G | Non-coding |
| X | 9703760 | t | A | Non-coding |
| X | 9703853 | t | C | Non-coding |
| X | 9748115 | * | -A | Non-coding |
| X | 9774476 | a | G | Non-coding |
| X | 9790629 | * | +CG | Non-coding |
| X | 9861507 | g | T | Nonsynonymous (R07B1.3) |
| X | 9882632 | a | C | Non-coding |
| X | 9999376 | * | +G | Non-coding |
| X | 9999591 | a | G | Non-coding |
| X | 10071939 | * | +C | Non-coding |
| X | 10130976 | t | G | Non-coding |
| X | 10179285 | * | +G | Non-coding |
| X | 10267286 | * | -C | Non-coding |
| X | 10290545 | * | +T | Non-coding |
| X | 10298114 | * | -T | Non-coding |
| X | 10421428 | g | A | Non-coding |
| X | 10439131 | c | T | Non-coding |
| X | 10463663 | * | +G | Non-coding |
| X | 10473878 | g | A | Non-coding |
| X | 10494128 | * | +A | Non-coding |
| X | 10591190 | g | T | Non-coding |
| X | 10594748 | * | +C | Non-coding |
| X | 10657028 | * | -A | Non-coding |
| X | 10661155 | * | +C | Non-coding |
| X | 10681314 | * | +C | Non-coding |
| X | 10707132 | * | -A | Non-coding |
| X | 10730281 | * | +G | Non-coding |
| X | 10841708 | * | -G | Non-coding |
| X | 10845087 | t | C | Non-coding |
| X | 10936020 | * | +G | Non-coding |
| X | 11158111 | t | A | Non-coding |
| X | 11166945 | * | -C | Non-coding |
| X | 11269067 | * | -G | Non-coding |
| X | 11314597 | * | +T | Non-coding |
| X | 11423704 | * | +C | exonic (*hum-4*) |
| X | 11428229 | * | +G | Non-coding |
| X | 11492871 | * | +T | Non-coding |
| X | 11564035 | * | +A | Non-coding |
| X | 11600446 | c | A | Non-coding |
| X | 11624629 | t | C | Nonsynonymous (Y79H9A.1) |
| X | 11654821 | * | -G | Non-coding |
| X | 11689186 | * | +G | Non-coding |
| X | 11690021 | * | +C | Non-coding |
| X | 11701453 | * | -G | Non-coding |
| X | 11791596 | * | +C | Non-coding |
| X | 11795876 | * | +G | Non-coding |
| X | 11831508 | c | A | Non-coding |
| X | 11831549 | t | C | Non-coding |
| X | 11896274 | * | +T | Non-coding |
| X | 12023120 | c | A | Non-coding |
| X | 12173865 | * | -C | Non-coding |
| X | 12277037 | * | -T | Non-coding |
| X | 12277112 | a | G | Non-coding |
| X | 12277116 | a | T | Non-coding |
| X | 12383571 | * | -C | Non-coding |
| X | 12416663 | * | -C | Non-coding |
| X | 12440274 | * | +C | Non-coding |
| X | 12492651 | * | +G | Non-coding |
| X | 12560995 | t | C | Non-coding |
| X | 12600323 | * | -C | Non-coding |
| X | 12642762 | * | +G | Non-coding |
| X | 12642997 | * | +C | Non-coding |
| X | 12653778 | * | +T | Non-coding |
| X | 12667067 | * | +T | Non-coding |
| X | 12701752 | * | -G | exonic (*nhr-214*) |
| X | 12710725 | * | -A | Non-coding |
| X | 12725119 | * | -T | Non-coding |
| X | 12786146 | g | C | Nonsynonymous (*alh-13*) |
| X | 12795967 | c | A | Non-coding |
| X | 12842429 | * | -A | Non-coding |
| X | 12842553 | * | -G | Non-coding |
| X | 12992783 | * | -A | Non-coding |
| X | 13006485 | * | +C | Non-coding |
| X | 13034301 | t | A | Non-coding |
| X | 13037332 | * | +T | Non-coding |
| X | 13041025 | * | +C | Non-coding |
| X | 13089008 | * | +T | Non-coding |
| X | 13119683 | * | +A | Non-coding |
| X | 13167540 | * | -C | Non-coding |
| X | 13354615 | * | -TG | Non-coding |
| X | 13380533 | * | -A | Non-coding |
| X | 13384402 | t | A | Non-coding |
| X | 13394674 | a | T | Non-coding |
| X | 13396050 | * | -T | Non-coding |
| X | 13404237 | * | +A | exonic (F02C12.1) |
| X | 13480349 | t | A | Synonymous (*srd-50*) |
| X | 13481139 | * | +G | Non-coding |
| X | 13484237 | * | +T | Non-coding |
| X | 13489526 | * | -TT | Non-coding |
| X | 13527873 | * | -G | Non-coding |
| X | 13649503 | g | C | Non-coding |
| X | 13652270 | * | -C | Non-coding |
| X | 13652600 | * | -G | Non-coding |
| X | 13656722 | * | +T | Non-coding |
| X | 13708212 | * | -T | Non-coding |
| X | 13731949 | * | +C | Non-coding |
| X | 13737752 | g | T | Non-coding |
| X | 13738978 | * | -G | Non-coding |
| X | 13864619 | * | -C | Non-coding |
| X | 13936891 | * | +C | Non-coding |
| X | 14031541 | * | +A | Non-coding |
| X | 14118935 | a | G | Non-coding |
| X | 14229404 | a | T | Non-coding |
| X | 14266896 | * | +A | Non-coding |
| X | 14269366 | * | +C | Non-coding |
| X | 14374966 | * | -A | Non-coding |
| X | 14375221 | * | +G | Non-coding |
| X | 14381923 | g | A | Non-coding |
| X | 14384687 | a | C | Non-coding |
| X | 14427613 | * | -T | Non-coding |
| X | 14455151 | * | +A | Non-coding |
| X | 14460891 | * | +G | Non-coding |
| X | 14461126 | * | +T | Non-coding |
| X | 14510694 | g | A | Non-coding |
| X | 14510701 | t | G | Non-coding |
| X | 14547380 | * | -A | Non-coding |
| X | 14573706 | * | -A | Non-coding |
| X | 14577774 | t | A | Non-coding |
| X | 14595714 | * | -A | exonic (C44H4.8) |
| X | 14613805 | c | A | Synonymous (F54E4.1) |
| X | 14614978 | * | +C | exonic (*rbc-1*) |
| X | 14634336 | * | +G | exonic (F54E4.3) |
| X | 14637664 | a | C | Non-coding |
| X | 14693202 | * | +G | Non-coding |
| X | 14732867 | * | -A | Non-coding |
| X | 14766614 | * | -G | exonic (Y16B4A.2) |
| X | 14766655 | * | -G | Non-coding |
| X | 14767044 | c | G | Synonymous (Y16B4A.2) |
| X | 14771512 | * | +A | Non-coding |
| X | 14776055 | a | T | Non-coding |
| X | 14814308 | * | -AT | Non-coding |
| X | 14913406 | g | T | Non-coding |
| X | 14917447 | * | +G | Non-coding |
| X | 14921904 | g | A | Non-coding |
| X | 14921912 | a | G | Non-coding |
| X | 14945493 | t | C | Non-coding |
| X | 14962448 | a | T | Non-coding |
| X | 14978678 | * | -A | Non-coding |
| X | 15192240 | * | -G | Non-coding |
| X | 15207904 | * | +T | Non-coding |
| X | 15226797 | g | T | Nonsynonymous (H03A11.2) |
| X | 15447753 | c | T | Non-coding |
| X | 15461257 | * | -A | Non-coding |
| X | 15472090 | a | T | Non-coding |
| X | 15520197 | a | G | Non-coding |
| X | 15707543 | * | +G | Non-coding |
| X | 15722216 | a | G | Non-coding |
| X | 15816871 | * | +T | Non-coding |
| X | 15836084 | a | G | Non-coding |
| X | 15873523 | * | -C | Non-coding |
| X | 15904379 | c | A | Non-coding |
| X | 15904380 | a | C | Non-coding |
| X | 15904850 | * | +C | Non-coding |
| X | 15945619 | a | T | Non-coding |
| X | 15945743 | g | T | Non-coding |
| X | 15945744 | g | T | Non-coding |
| X | 15945755 | a | T | Non-coding |
| X | 15953621 | * | +G | Non-coding |
| X | 16010510 | * | -A | Non-coding |
| X | 16010916 | g | T | Non-coding |
| X | 16010919 | c | A | Non-coding |
| X | 16025702 | * | +A | Non-coding |
| X | 16029172 | * | +T | Non-coding |
| X | 16083029 | * | +A | Non-coding |
| X | 16087814 | * | -A | Non-coding |
| X | 16102626 | t | G | Non-coding |
| X | 16120767 | * | -G | Non-coding |
| X | 16180317 | * | +C | Non-coding |
| X | 16221133 | * | +G | Non-coding |
| X | 16224213 | * | +G | Non-coding |
| X | 16250557 | * | +C | Non-coding |
| X | 16255156 | g | T | Non-coding |
| X | 16255668 | t | G | Non-coding |
| X | 16255669 | t | C | Non-coding |
| X | 16255728 | t | C | Non-coding |
| X | 16255860 | t | A | Non-coding |
| X | 16256025 | * | -T | Non-coding |
| X | 16292255 | * | -G | Non-coding |
| X | 16302440 | t | A | Non-coding |
| X | 16446898 | * | -C | Non-coding |
| X | 16460133 | c | A | Non-coding |
| X | 16460180 | c | A | Non-coding |
| X | 16461404 | * | +C | Non-coding |
| X | 16661001 | g | T | Non-coding |
| X | 16688960 | * | +T | Non-coding |
| X | 16774059 | * | -T | Non-coding |
| X | 16787261 | a | T | Non-coding |
| X | 16817032 | t | C | Nonsynonymous (*sto-5*) |
| X | 16959637 | * | +T | Non-coding |
| X | 16981415 | t | C | Non-coding |
| X | 16985780 | * | +T | Non-coding |
| X | 17025320 | a | T | Non-coding |
| X | 17047302 | c | T | Nonsynonymous (*gcy-11*) |
| X | 17047309 | t | C | Nonsynonymous (*gcy-11*) |
| X | 17047323 | g | T | Nonsynonymous (*gcy-11*) |
| X | 17054554 | * | +C | Non-coding |
| X | 17086379 | * | -C | Non-coding |
| X | 17097607 | t | A | Non-coding |
| X | 17105478 | g | C | Non-coding |
| X | 17105846 | * | +T | Non-coding |
| X | 17168879 | a | G | Non-coding |
| X | 17228978 | * | +G | exonic (T25G12.6) |
| X | 17298448 | * | -A | Non-coding |
| X | 17378481 | * | -A | Non-coding |
| X | 17443243 | * | +C | Non-coding |
| X | 17486061 | * | +T | Non-coding |
| X | 17492029 | * | +A | Non-coding |
| X | 17591176 | * | +C | Non-coding |
| X | 17621416 | a | T | Non-coding |
| X | 17714409 | * | +C | exonic (6R55.2) |
| X | 17715334 | g | A | Non-coding |
