# Supplementary material for: Whole Genome Sequencing Highlights Genetic Changes Associated with Laboratory Domestication of C. elegans
Source: PLoS One. 2010 Nov 11;5(11):e13922. doi: 10.1371/journal.pone.0013922 (PMC2978686; doi:10.1371/journal.pone.0013922)
Supplement: Table S2 — N2/LSJ1 changes accumulated during domestication: SNPs and small indels between N2 and LSJ1 that are retained after filtering for N2 reference sequence errors. (1.29 MB DOC) [file pone.0013922.s005.doc]

| **Chromosome** | **Coordinate** | **N2 allele** | **LSJ1 allele** | **Ancestral State** | **Type (Gene)** |
| --- | --- | --- | --- | --- | --- |
| I | 33252 | g | A | N2 | Non-coding |
| I | 229064 | c | T | LSJ1 | Non-coding |
| I | 229535 | g | T | N2 | Nonsynonymous (Y48G1BM.6) |
| I | 238077 | t | G | N2 | Nonsynonymous (Y48G1BM.6) |
| I | 274679 | * | +CCCC | N2 | Non-coding |
| I | 318214 | * | +G | LSJ1 | Non-coding |
| I | 545660 | * | +C | N2 | Non-coding |
| I | 670445 | * | -C | N2 | Non-coding |
| I | 839307 | c | A | LSJ1 | Non-coding |
| I | 856611 | g | T | N2 | Non-coding |
| I | 918054 | t | C | N2 | Non-coding |
| I | 918055 | t | G | LSJ1 | Non-coding |
| I | 939941 | t | A | N2 | Non-coding |
| I | 973332 | * | -GG | N2 | Non-coding |
| I | 985200 | * | -G | N2 | Non-coding |
| I | 1027642 | t | A | LSJ1 | Non-coding |
| I | 1070573 | g | A | N2 | Non-coding |
| I | 1077809 | a | T | N2 | Non-coding |
| I | 1205754 | * | +AATCTACACAGTAG | N2 | Non-coding |
| I | 1376719 | * | -G | N2 | Non-coding |
| I | 1382017 | t | A | unknown | Non-coding |
| I | 1388449 | * | -A | LSJ1 | Non-coding |
| I | 1395885 | * | +C | N2 | Non-coding |
| I | 1473630 | * | +AAA | N2 | Non-coding |
| I | 1481615 | * | +CT | N2 | Non-coding |
| I | 1507378 | a | T | N2 | Non-coding |
| I | 1612538 | * | +A | unknown | Non-coding |
| I | 1708012 | t | A | LSJ1 | Non-coding |
| I | 1749020 | c | T | N2 | Non-coding |
| I | 1766667 | c | A | LSJ1 | Non-coding |
| I | 1768944 | * | -A | LSJ1 | Non-coding |
| I | 1865218 | g | A | N2 | Non-coding |
| I | 2002844 | t | C | LSJ1 | Non-coding |
| I | 2312091 | * | -T | N2 | Non-coding |
| I | 2403789 | a | T | N2 | Non-coding |
| I | 2822542 | * | +A | LSJ1 | Non-coding |
| I | 2980358 | * | +C | N2 | Non-coding |
| I | 2991806 | a | G | LSJ1 | Non-coding |
| I | 2991822 | g | T | LSJ1 | Non-coding |
| I | 2991829 | t | C | LSJ1 | Non-coding |
| I | 3105323 | * | +G | N2 | Non-coding |
| I | 3115352 | g | T | N2 | Non-coding |
| I | 3174597 | c | A | N2 | Non-coding |
| I | 3184795 | c | T | N2 | Non-coding |
| I | 3400628 | c | A | LSJ1 | Non-coding |
| I | 3400638 | c | A | N2 | Non-coding |
| I | 3459102 | c | A | LSJ1 | Non-coding |
| I | 3487596 | * | -A | LSJ1 | Non-coding |
| I | 3515116 | a | G | LSJ1 | Non-coding |
| I | 3515187 | c | G | LSJ1 | Non-coding |
| I | 3515205 | a | G | N2 | Non-coding |
| I | 3541187 | t | A | N2 | Non-coding |
| I | 3575785 | c | T | LSJ1 | Non-coding |
| I | 3895275 | g | T | N2 | 3' UTR (C50F2.1) |
| I | 3929828 | t | A | N2 | Non-coding |
| I | 3990728 | c | A | N2 | Non-coding |
| I | 4260706 | * | +A |  | Non-coding |
| I | 4543875 | t | G | N2 | Non-coding |
| I | 4719687 | * | -GG | N2 | Non-coding |
| I | 4785682 | c | G | N2 | Non-coding |
| I | 4895808 | * | -T | N2 | Non-coding |
| I | 5351199 | t | C | LSJ1 | Nonsynonymous (F55A12.8) |
| I | 5495788 | * | +C | N2 | Non-coding |
| I | 5939043 | t | A | N2 | Non-coding |
| I | 6066249 | * | +GATCTCCAATTACAATCAAAA |  | Non-coding |
| I | 6121037 | g | T | N2 | Non-coding |
| I | 6345100 | c | T | N2 | Non-coding |
| I | 6604645 | * | +G | N2 | Non-coding |
| I | 7284291 | * | -CTGTAT | N2 | Non-coding |
| I | 7411341 | c | A | N2 | Non-coding |
| I | 7666737 | * | +G | N2 | Non-coding |
| I | 7780078 | a | C | N2 | Non-coding |
| I | 7780082 | g | T | N2 | Non-coding |
| I | 7879503 | * | +A | N2 | Non-coding |
| I | 8881330 | * | +A | N2 | exonic (F55H12.3) |
| I | 9053688 | c | A | N2 | Non-coding |
| I | 9053689 | t | A | N2 | Non-coding |
| I | 9534656 | * | +AA | N2 | Non-coding |
| I | 9569799 | a | T | N2 | Non-coding |
| I | 9831395 | a | T | N2 | Non-coding |
| I | 10057937 | t | C | N2 | Non-coding |
| I | 10225824 | c | A | N2 | Non-coding |
| I | 10239251 | * | +G | LSJ1 | Non-coding |
| I | 10267627 | * | +T | N2 | exonic (ZC247.1) |
| I | 10354198 | * | -T | N2 | Non-coding |
| I | 10360279 | t | A | N2 | Non-coding |
| I | 10368179 | g | T | N2 | Non-coding |
| I | 10579332 | * | +C | LSJ1 | Non-coding |
| I | 10636544 | * | +GGG | N2 | Non-coding |
| I | 10749095 | g | A | N2 | Non-coding |
| I | 10749104 | g | A | N2 | Non-coding |
| I | 11055187 | c | A | N2 | Non-coding |
| I | 11130843 | t | C | LSJ1 | Non-coding |
| I | 11511646 | c | A | N2 | Non-coding |
| I | 11511648 | c | T | N2 | Non-coding |
| I | 11511956 | c | A | LSJ1 | Non-coding |
| I | 11511968 | c | A | LSJ1 | Non-coding |
| I | 11576999 | c | T | N2 | Non-coding |
| I | 11600408 | a | T | N2 | Non-coding |
| I | 11868441 | * | +A | N2 | Non-coding |
| I | 11949186 | * | +A | N2 | Non-coding |
| I | 12406484 | a | G | N2 | Synonymous (E03H4.3) |
| I | 12856660 | * | +T | LSJ1 | Non-coding |
| I | 12971111 | c | A | N2 | Non-coding |
| I | 13089055 | c | T | N2 | Nonsynonymous (Y26D4A.9) |
| I | 13089056 | a | G | N2 | Synonymous (Y26D4A.9) |
| I | 13284381 | g | C | LSJ1 | Non-coding |
| I | 13297005 | c | T | N2 | 3' UTR (*top-1*) |
| I | 13440385 | t | A | unknown | Non-coding |
| I | 13484855 | * | -TTC | N2 | Non-coding |
| I | 13531689 | a | T | N2 | Non-coding |
| I | 13545631 | g | A | LSJ1 | Non-coding |
| I | 13618450 | * | +T | N2 | Non-coding |
| I | 13753024 | a | T | N2 | Non-coding |
| I | 13946479 | g | A | LSJ1 | Non-coding |
| I | 14123562 | g | A | N2 | Non-coding |
| I | 14124786 | * | +AA | LSJ1 | Non-coding |
| I | 14138251 | * | +CC | N2 | Non-coding |
| I | 14448365 | * | +A | N2 | Non-coding |
| I | 14524588 | * | +A | N2 | Non-coding |
| I | 14634485 | c | A | N2 | 3' UTR (*exoc-8*) |
| I | 14796971 | c | T | N2 | Synonymous (K11B4.1) |
| I | 14876643 | * | +T | LSJ1 | Non-coding |
| I | 14945523 | * | -C | N2 | Non-coding |
| I | 15057045 | g | T | N2 | Non-coding |
| II | 5570 | * | +G | N2 | Non-coding |
| II | 73323 | c | A | N2 | Nonsynonymous (C23H3.9) |
| II | 184004 | t | A | N2 | Non-coding |
| II | 207834 | g | T | N2 | Nonsynonymous (*chs-2*) |
| II | 377735 | * | -C | N2 | Non-coding |
| II | 493037 | a | T | N2 | Non-coding |
| II | 696778 | a | C | N2 | Non-coding |
| II | 696864 | g | A | N2 | Non-coding |
| II | 698391 | t | C | N2 | Non-coding |
| II | 704809 | a | T | N2 | Non-coding |
| II | 732210 | * | -C | N2 | Non-coding |
| II | 1084658 | * | -G | N2 | Non-coding |
| II | 1093728 | t | A | N2 | Non-coding |
| II | 1213695 | * | +G | N2 | Non-coding |
| II | 1248241 | a | T | N2 | Non-coding |
| II | 1295580 | * | +C | LSJ1 | Non-coding |
| II | 1298438 | a | C | N2 | Non-coding |
| II | 1455171 | a | T | N2 | Non-coding |
| II | 1605261 | a | C | N2 | Non-coding |
| II | 1605262 | a | C | N2 | Non-coding |
| II | 1771115 | * | -GG | LSJ1 | Non-coding |
| II | 2139855 | * | +GGG | N2 | Non-coding |
| II | 2221680 | g | A | N2 | Non-coding |
| II | 2225250 | g | T | LSJ1 | Non-coding |
| II | 2225256 | a | T | N2 | Non-coding |
| II | 2225268 | g | T | N2 | Non-coding |
| II | 2225271 | a | C | N2 | Non-coding |
| II | 2298880 | g | A | N2 | Non-coding |
| II | 2298887 | g | A | N2 | Non-coding |
| II | 2396167 | g | A | N2 | Non-coding |
| II | 2442548 | * | -T | LSJ1 | Non-coding |
| II | 2620557 | * | -A | LSJ1 | Non-coding |
| II | 2639354 | c | A | LSJ1 | Non-coding |
| II | 2861213 | * | -G | N2 | Non-coding |
| II | 2881221 | t | G | N2 | Non-coding |
| II | 2898769 | t | A | N2 | Non-coding |
| II | 2898985 | g | T | N2 | Non-coding |
| II | 2898988 | t | A | N2 | Non-coding |
| II | 2935801 | g | A | N2 | Non-coding |
| II | 2935808 | g | A | N2 | Non-coding |
| II | 2936480 | g | A | N2 | Non-coding |
| II | 2936627 | g | A | N2 | Non-coding |
| II | 2973842 | * | +C | N2 | Non-coding |
| II | 2974233 | t | G | N2 | Non-coding |
| II | 2974267 | * | +C | N2 | Non-coding |
| II | 2997907 | * | +T | LSJ1 | Non-coding |
| II | 3219418 | * | +T | N2 | Non-coding |
| II | 3375963 | * | -GGGGG | N2 | Non-coding |
| II | 3429000 | g | T | N2 | Non-coding |
| II | 3465795 | c | G | N2 | Non-coding |
| II | 3539915 | g | A | N2 | Non-coding |
| II | 3539934 | g | T | LSJ1 | Non-coding |
| II | 3539935 | a | T | N2 | Non-coding |
| II | 3926242 | g | T | N2 | Non-coding |
| II | 3926243 | a | T | N2 | Non-coding |
| II | 4284912 | * | +A | N2 | Non-coding |
| II | 4368150 | * | +C | LSJ1 | exonic (*lat-2*) |
| II | 4396221 | * | -C | N2 | Non-coding |
| II | 4400120 | t | A | N2 | Non-coding |
| II | 4642239 | * | -C | LSJ1 | Non-coding |
| II | 4642293 | * | -A | N2 | Non-coding |
| II | 4821506 | a | G | LSJ1 | Nonsynonymous (*lact-5*) |
| II | 4873888 | g | T | N2 | Non-coding |
| II | 4873894 | g | T | N2 | Non-coding |
| II | 5146755 | a | G | N2 | Non-coding |
| II | 5268591 | * | -G | N2 | Non-coding |
| II | 5337132 | c | A | N2 | Non-coding |
| II | 5340537 | a | T | N2 | 3' UTR (*vhp-1*) |
| II | 5340547 | a | T | N2 | 3' UTR (*vhp-1*) |
| II | 5601337 | a | C | LSJ1 | Nonsynonymous (C17G10.6) |
| II | 5858526 | c | A | N2 | Non-coding |
| II | 6084903 | * | +G | LSJ1 | exonic (*ptc-2*) |
| II | 6136222 | t | C | N2 | Nonsynonymous (*abts-3*) |
| II | 6163739 | t | C | N2 | Non-coding |
| II | 6163749 | g | C | N2 | Non-coding |
| II | 6297145 | a | T | N2 | Non-coding |
| II | 6297148 | a | T | N2 | Non-coding |
| II | 6334728 | * | +T | LSJ1 | Non-coding |
| II | 6730765 | * | -C | LSJ1 | Non-coding |
| II | 6805684 | * | -A | LSJ1 | Non-coding |
| II | 6908081 | * | +T | LSJ1 | Non-coding |
| II | 7083616 | a | G | N2 | Synonymous (T02G5.4) |
| II | 7123986 | * | +G | N2 | exonic (F21H12.4) |
| II | 7339361 | t | C | LSJ1 | Non-coding |
| II | 7371718 | c | G | N2 | Non-coding |
| II | 7642036 | * | +G | LSJ1 | exonic (*pde-4*) |
| II | 7698878 | * | +G | LSJ1 | exonic (B0495.6) |
| II | 7883556 | * | +A | N2 | Non-coding |
| II | 7949738 | c | A | N2 | Non-coding |
| II | 8145659 | * | +G | N2 | Non-coding |
| II | 8746874 | * | -C | N2 | exonic (K08F8.1) |
| II | 9497203 | * | +TCTC | N2 | Non-coding |
| II | 9563143 | * | +A | LSJ1 | Non-coding |
| II | 9684873 | g | A | N2 | Non-coding |
| II | 9789703 | t | C | LSJ1 | Non-coding |
| II | 9863937 | c | T | LSJ1 | Nonsynonymous (C08H9.4) |
| II | 9863960 | a | G | LSJ1 | Nonsynonymous (C08H9.4) |
| II | 9989552 | g | A | LSJ1 | Non-coding |
| II | 10135441 | * | +C | N2 | Non-coding |
| II | 10567422 | g | A | N2 | Non-coding |
| II | 10750971 | a | G | N2 | Non-coding |
| II | 10779220 | g | A | LSJ1 | Non-coding |
| II | 10827352 | c | A | LSJ1 | Non-coding |
| II | 10946735 | g | T | N2 | Non-coding |
| II | 11046101 | c | A | N2 | 3' UTR (F33A8.4) |
| II | 11138252 | g | A | N2 | Non-coding |
| II | 11190204 | c | A | N2 | Non-coding |
| II | 11402552 | * | -G | N2 | Non-coding |
| II | 11555135 | a | T | N2 | Non-coding |
| II | 11555137 | c | T | N2 | Non-coding |
| II | 11555162 | c | T | LSJ1 | Non-coding |
| II | 11555167 | c | T | LSJ1 | Non-coding |
| II | 12076573 | t | A | N2 | Non-coding |
| II | 12232153 | a | G | N2 | Non-coding |
| II | 12268789 | c | A | N2 | Non-coding |
| II | 12271956 | g | T | N2 | Non-coding |
| II | 12420841 | a | G | N2 | Non-coding |
| II | 12545593 | * | +G | N2 | Non-coding |
| II | 12649965 | c | T | N2 | Non-coding |
| II | 12650503 | t | C | N2 | Non-coding |
| II | 12650505 | t | C | N2 | Non-coding |
| II | 12730042 | c | T | N2 | Nonsynonymous (Y46G5A.4) |
| II | 12769767 | * | +CG | N2 | Non-coding |
| II | 12791685 | t | A | N2 | Non-coding |
| II | 13221214 | * | +G | LSJ1 | Non-coding |
| II | 13225391 | * | +CC | N2 | Non-coding |
| II | 13310913 | t | A | N2 | Non-coding |
| II | 13407777 | g | A | N2 | Non-coding |
| II | 13415878 | * | +C | N2 | Non-coding |
| II | 13533774 | * | -T | LSJ1 | Non-coding |
| II | 13721835 | g | A | N2 | Non-coding |
| II | 13844733 | g | T | N2 | Non-coding |
| II | 13935724 | c | A | LSJ1 | Non-coding |
| II | 13986666 | a | T | N2 | Non-coding |
| II | 14275383 | * | +G | N2 | Non-coding |
| II | 14312057 | * | -G | N2 | Non-coding |
| II | 14338274 | c | T | N2 | Non-coding |
| II | 14338283 | t | G | N2 | Non-coding |
| II | 14346720 | t | A | LSJ1 | Non-coding |
| II | 14346732 | c | A | LSJ1 | Non-coding |
| II | 14392278 | t | A | N2 | Non-coding |
| II | 14407665 | * | +CGA | N2 | exonic (F26H11.2) |
| II | 15051354 | g | T | LSJ1 | Non-coding |
| II | 15093087 | a | T | N2 | Non-coding |
| II | 15140302 | * | +T | N2 | Non-coding |
| II | 15189033 | t | G | N2 | Non-coding |
| II | 15189034 | c | T | N2 | Non-coding |
| II | 15243778 | * | +T | N2 | Non-coding |
| III | 90805 | t | A | N2 | Non-coding |
| III | 281738 | * | -T | N2 | Non-coding |
| III | 303837 | a | T | LSJ1 | Non-coding |
| III | 313340 | g | A | N2 | Non-coding |
| III | 336186 | a | C | N2 | Nonsynonymous (Y50D7A.8) |
| III | 347566 | c | T | N2 | Non-coding |
| III | 363562 | * | +C | N2 | Non-coding |
| III | 406624 | * | +T | N2 | Non-coding |
| III | 445783 | * | -ACTACAA | N2 | Non-coding |
| III | 445803 | t | G | N2 | Non-coding |
| III | 483443 | g | A | N2 | Non-coding |
| III | 498684 | * | -G | N2 | Non-coding |
| III | 502009 | c | T | N2 | Nonsynonymous (*unc-45*) |
| III | 578505 | t | C | N2 | Non-coding |
| III | 657302 | * | +G | N2 | Non-coding |
| III | 876193 | * | -G | N2 | Non-coding |
| III | 1210663 | g | A | N2 | Non-coding |
| III | 1300104 | g | T | N2 | Non-coding |
| III | 1300105 | a | C | N2 | Non-coding |
| III | 1300147 | * | +C | N2 | Non-coding |
| III | 1530006 | * | +A | N2 | Non-coding |
| III | 1785791 | * | +AAAAA | LSJ1 | Non-coding |
| III | 1883377 | t | C | N2 | Non-coding |
| III | 1933068 | a | T | N2 | Non-coding |
| III | 2079855 | * | +C | N2 | Non-coding |
| III | 2104232 | a | G | N2 | Non-coding |
| III | 2187048 | c | T | LSJ1 | Non-coding |
| III | 2261453 | t | A | LSJ1 | Non-coding |
| III | 2282496 | t | G | LSJ1 | Non-coding |
| III | 2282503 | a | T | LSJ1 | Non-coding |
| III | 2297023 | a | T | N2 | Non-coding |
| III | 2349316 | g | A | N2 | Non-coding |
| III | 2394676 | c | T | N2 | Non-coding |
| III | 2471351 | * | +G | LSJ1 | Non-coding |
| III | 2498702 | c | A | N2 | Non-coding |
| III | 2692650 | * | -T | N2 | Non-coding |
| III | 2718308 | t | A | N2 | 3' UTR (Y71H2AM.11) |
| III | 2874465 | a | T | N2 | Non-coding |
| III | 2874467 | c | T | N2 | Non-coding |
| III | 3244293 | c | G | N2 | Non-coding |
| III | 3375231 | c | T | LSJ1 | Non-coding |
| III | 3385111 | * | +G | N2 | Non-coding |
| III | 3385299 | * | +G | N2 | Non-coding |
| III | 3385876 | * | +G | N2 | Non-coding |
| III | 3416291 | c | T | N2 | Non-coding |
| III | 3480534 | * | +CC | N2 | Non-coding |
| III | 3635715 | * | +C | N2 | Non-coding |
| III | 3681015 | t | G | N2 | Non-coding |
| III | 3933546 | t | A | N2 | Non-coding |
| III | 3953890 | * | -A | LSJ1 | Non-coding |
| III | 4209221 | c | A | LSJ1 | Non-coding |
| III | 4209243 | c | A | LSJ1 | Non-coding |
| III | 4476799 | t | A | N2 | 3' UTR (*rps-1*) |
| III | 4484614 | * | +G | LSJ1 | Non-coding |
| III | 4684466 | c | T | LSJ1 | Pseudogene (T04A8.3) |
| III | 4833738 | a | G | N2 | Non-coding |
| III | 4835340 | t | C | N2 | Non-coding |
| III | 4835342 | t | G | N2 | Non-coding |
| III | 5468976 | c | T | N2 | Non-coding |
| III | 5796420 | g | T | N2 | Non-coding |
| III | 6037552 | * | +C | LSJ1 | Non-coding |
| III | 6172575 | a | T | N2 | Synonymous (F42A10.5) |
| III | 6283154 | a | T | N2 | Non-coding |
| III | 6301652 | * | +C | N2 | Non-coding |
| III | 6371226 | a | G | LSJ1 | Nonsynonymous (*thoc-2*) |
| III | 6608758 | g | T | N2 | Synonymous (F20H11.4) |
| III | 6619218 | c | T | LSJ1 | Nonsynonymous (Y40D12A.1) |
| III | 6626308 | * | -G | LSJ1 | Non-coding |
| III | 6688070 | * | -AT | N2 | Non-coding |
| III | 6813217 | c | T | N2 | Non-coding |
| III | 6896598 | c | A | LSJ1 | Non-coding |
| III | 6923581 | g | A | LSJ1 | Non-coding |
| III | 6990263 | t | C | LSJ1 | Non-coding |
| III | 7013481 | g | A | LSJ1 | Non-coding |
| III | 7028625 | a | T | LSJ1 | Non-coding |
| III | 7028660 | c | T | LSJ1 | Nonsynonymous (*cyk-1*) |
| III | 7033327 | t | G | LSJ1 | Non-coding |
| III | 7063386 | * | +C | N2 | Non-coding |
| III | 7118007 | g | A | LSJ1 | Non-coding |
| III | 7163701 | a | C | LSJ1 | Nonsynonymous (K04G7.1) |
| III | 7189700 | c | T | LSJ1 | Non-coding |
| III | 7214106 | a | C | LSJ1 | Nonsynonymous (R151.2) |
| III | 7235775 | * | +GC | N2 | Non-coding |
| III | 7238031 | * | +C | N2 | Non-coding |
| III | 7325087 | g | T | LSJ1 | Nonsynonymous (F56C9.11) |
| III | 7334121 | t | C | LSJ1 | Non-coding |
| III | 7334155 | g | C | LSJ1 | Non-coding |
| III | 7415329 | * | +A | N2 | Non-coding |
| III | 7423223 | * | +G | N2 | Non-coding |
| III | 7446597 | c | A | N2 | Non-coding |
| III | 7446612 | g | A | N2 | Non-coding |
| III | 7446619 | c | A | N2 | Non-coding |
| III | 7448194 | t | C | N2 | Non-coding |
| III | 7588320 | a | G | LSJ1 | Non-coding |
| III | 7595084 | * | -CA | N2 | Non-coding |
| III | 7643356 | t | A | LSJ1 | Non-coding |
| III | 7713783 | * | +G | N2 | Non-coding |
| III | 7799405 | c | T | LSJ1 | Non-coding |
| III | 7924183 | c | T | LSJ1 | Non-coding |
| III | 7958665 | c | T | LSJ1 | Non-coding |
| III | 8064606 | c | T | LSJ1 | Non-coding |
| III | 8124696 | g | A | LSJ1 | Non-coding |
| III | 8130398 | g | A | LSJ1 | Non-coding |
| III | 8154519 | t | A | LSJ1 | Non-coding |
| III | 8424573 | t | C | LSJ1 | Non-coding |
| III | 8433741 | * | -C | N2 | Non-coding |
| III | 8441333 | * | +G | N2 | Non-coding |
| III | 8503009 | a | T | LSJ1 | Non-coding |
| III | 8563933 | g | A | LSJ1 | Non-coding |
| III | 8621239 | c | T | LSJ1 | Non-coding |
| III | 8684181 | t | A | LSJ1 | Non-coding |
| III | 8684210 | c | T | LSJ1 | Non-coding |
| III | 8693609 | g | C | LSJ1 | Nonsynonymous (B0303.7) |
| III | 8693613 | g | A | LSJ1 | Synonymous (B0303.7) |
| III | 8693775 | a | T | LSJ1 | Non-coding |
| III | 8701739 | c | T | LSJ1 | Synonymous (*vps-33.1*) |
| III | 8704002 | t | A | LSJ1 | Non-coding |
| III | 8710966 | t | A | LSJ1 | Non-coding |
| III | 8711828 | c | T | LSJ1 | Non-coding |
| III | 8712015 | g | A | LSJ1 | Non-coding |
| III | 8733584 | a | T | LSJ1 | Non-coding |
| III | 8733613 | a | T | LSJ1 | Non-coding |
| III | 8736390 | g | A | LSJ1 | Non-coding |
| III | 8740763 | c | T | LSJ1 | Non-coding |
| III | 8742382 | g | A | N2 | 3' UTR (ZK370.6) |
| III | 8744443 | c | T | LSJ1 | Non-coding |
| III | 8752724 | c | T | LSJ1 | Non-coding |
| III | 8758067 | t | C | LSJ1 | Non-coding |
| III | 8810352 | * | -C | LSJ1 | Non-coding |
| III | 9013700 | * | +G | LSJ1 | Non-coding |
| III | 9025487 | a | T | LSJ1 | Non-coding |
| III | 9066058 | g | C | LSJ1 | Non-coding |
| III | 9101168 | c | T | LSJ1 | Nonsynonymous (ZK507.1) |
| III | 9101192 | t | A | N2 | Non-coding |
| III | 9101201 | g | A | N2 | Non-coding |
| III | 9102158 | * | -C | N2 | Non-coding |
| III | 9229032 | t | A | N2 | Non-coding |
| III | 9259899 | a | G | N2 | Nonsynonymous (M01A8.1) |
| III | 9263155 | g | A | LSJ1 | Non-coding |
| III | 9264265 | g | A | LSJ1 | Non-coding |
| III | 9282274 | * | +C | LSJ1 | Non-coding |
| III | 9298566 | c | T | LSJ1 | Non-coding |
| III | 9341371 | t | C | LSJ1 | Nonsynonymous (*emb-9*) |
| III | 9350630 | c | T | LSJ1 | Nonsynonymous (K04H4.2) |
| III | 9350655 | c | T | LSJ1 | Nonsynonymous (K04H4.2) |
| III | 9350662 | c | T | LSJ1 | Synonymous (K04H4.2) |
| III | 9352563 | c | T | LSJ1 | Synonymous (K04H4.2) |
| III | 9355600 | a | C | LSJ1 | Non-coding |
| III | 9355601 | c | G | LSJ1 | Non-coding |
| III | 9409058 | t | G | LSJ1 | Non-coding |
| III | 9409294 | c | G | LSJ1 | Non-coding |
| III | 9417284 | g | T | LSJ1 | Non-coding |
| III | 9448447 | c | G | LSJ1 | Non-coding |
| III | 9449799 | c | T | LSJ1 | Non-coding |
| III | 9450595 | c | A | LSJ1 | Non-coding |
| III | 9451507 | g | C | LSJ1 | miRNA target (F54C8.7 3'UTR) |
| III | 9452441 | g | T | LSJ1 | Non-coding |
| III | 9479168 | g | A | LSJ1 | Non-coding |
| III | 9571258 | * | -A | N2 | Non-coding |
| III | 9583727 | a | T | LSJ1 | Non-coding |
| III | 9643536 | c | G | LSJ1 | Non-coding |
| III | 9643537 | g | C | LSJ1 | Non-coding |
| III | 9647730 | * | -G | LSJ1 | Non-coding |
| III | 9675042 | a | T | LSJ1 | Non-coding |
| III | 9754432 | c | A | LSJ1 | Nonsynonymous (T05G5.8) |
| III | 9785416 | * | -A | N2 | exonic (R10E11.6) |
| III | 9796156 | t | C | LSJ1 | Non-coding |
| III | 9913768 | c | A | LSJ1 | Non-coding |
| III | 9918744 | c | G | LSJ1 | Synonymous (F40F12.3) |
| III | 9920858 | g | T | LSJ1 | Non-coding |
| III | 9940473 | c | A | LSJ1 | Non-coding |
| III | 9940559 | t | A | N2 | Non-coding |
| III | 9940568 | a | G | LSJ1 | Non-coding |
| III | 9942891 | t | A | LSJ1 | Non-coding |
| III | 9956573 | a | C | LSJ1 | Non-coding |
| III | 10019934 | g | T | LSJ1 | Non-coding |
| III | 10035097 | g | T | LSJ1 | Non-coding |
| III | 10035119 | g | T | LSJ1 | Non-coding |
| III | 10035130 | g | T | LSJ1 | Non-coding |
| III | 10035141 | c | T | LSJ1 | Non-coding |
| III | 10035172 | c | T | LSJ1 | Non-coding |
| III | 10035186 | a | T | LSJ1 | Non-coding |
| III | 10035311 | g | T | LSJ1 | Non-coding |
| III | 10035386 | g | A | LSJ1 | Non-coding |
| III | 10035427 | c | A | LSJ1 | Non-coding |
| III | 10036025 | g | A | LSJ1 | Non-coding |
| III | 10123054 | g | T | LSJ1 | Non-coding |
| III | 10123369 | c | G | LSJ1 | Non-coding |
| III | 10123412 | * | -T | N2 | Non-coding |
| III | 10123514 | c | T | N2 | Non-coding |
| III | 10148033 | c | G | LSJ1 | Non-coding |
| III | 10226007 | g | A | LSJ1 | Non-coding |
| III | 10277509 | c | T | LSJ1 | Non-coding |
| III | 10287325 | c | T | LSJ1 | Non-coding |
| III | 10289346 | g | C | LSJ1 | Non-coding |
| III | 10291758 | t | A | LSJ1 | Non-coding |
| III | 10354932 | a | T | LSJ1 | 3' UTR (T07C4.3) |
| III | 10361525 | g | A | LSJ1 | Non-coding |
| III | 10361913 | t | G | LSJ1 | Non-coding |
| III | 10368135 | g | A | LSJ1 | Non-coding |
| III | 10368775 | c | G | LSJ1 | Synonymous (*tbc-8*) |
| III | 10373198 | * | +G | N2 | Non-coding |
| III | 10382540 | t | A | N2 | Non-coding |
| III | 10382542 | t | A | N2 | Non-coding |
| III | 10399086 | c | A | LSJ1 | Non-coding |
| III | 10454271 | g | A | LSJ1 | Non-coding |
| III | 10482441 | g | A | LSJ1 | Non-coding |
| III | 10498009 | g | A | N2 | Non-coding |
| III | 10500604 | g | A | LSJ1 | Synonymous (*rab-33*) |
| III | 10500800 | c | T | LSJ1 | Non-coding |
| III | 10500856 | c | T | LSJ1 | Non-coding |
| III | 10501172 | c | T | LSJ1 | Non-coding |
| III | 10502430 | g | A | LSJ1 | Non-coding |
| III | 10502728 | c | T | LSJ1 | Non-coding |
| III | 10503430 | g | A | LSJ1 | Non-coding |
| III | 10511198 | * | +T |  | Non-coding |
| III | 10512575 | c | T | LSJ1 | 3' UTR (F43D9.3) |
| III | 10512827 | c | T | LSJ1 | 3' UTR (F43D9.3) |
| III | 10513934 | c | T | LSJ1 | Non-coding |
| III | 10514117 | g | A | LSJ1 | Non-coding |
| III | 10514382 | * | +C |  | Non-coding |
| III | 10515354 | g | C | LSJ1 | Non-coding |
| III | 10520300 | a | C | LSJ1 | Non-coding |
| III | 10528189 | g | C | LSJ1 | Non-coding |
| III | 10557467 | a | T | LSJ1 | Non-coding |
| III | 10778277 | * | +AA | N2 | Non-coding |
| III | 10890795 | g | A | N2 | Non-coding |
| III | 11016887 | t | C | LSJ1 | Non-coding |
| III | 11057656 | * | +AA | LSJ1 | Non-coding |
| III | 11088425 | g | T | N2 | Non-coding |
| III | 11114147 | a | G | LSJ1 | Non-coding |
| III | 11114148 | a | T | LSJ1 | Non-coding |
| III | 11114166 | t | A | LSJ1 | Non-coding |
| III | 11127080 | t | A | N2 | Non-coding |
| III | 11161855 | c | A | N2 | Non-coding |
| III | 11342769 | * | +AAAAATTCA | N2 | Non-coding |
| III | 11472335 | t | A | LSJ1 | Non-coding |
| III | 11480575 | g | A | LSJ1 | Non-coding |
| III | 11486303 | g | C | LSJ1 | Non-coding |
| III | 11503256 | t | G | N2 | Nonsynonymous (Y66D12A.21) |
| III | 11503425 | t | C | N2 | Nonsynonymous (Y66D12A.21) |
| III | 11503446 | t | C | N2 | Nonsynonymous (Y66D12A.21) |
| III | 11516789 | c | T | LSJ1 | Non-coding |
| III | 11518753 | g | A | N2 | Non-coding |
| III | 11518765 | c | A | N2 | Non-coding |
| III | 11562915 | c | T | N2 | Non-coding |
| III | 11562922 | t | G | N2 | Non-coding |
| III | 11572308 | c | A | N2 | Non-coding |
| III | 11576715 | c | G | LSJ1 | Nonsynonymous (Y66D12A.8) |
| III | 11580018 | c | A | LSJ1 | Non-coding |
| III | 11591932 | * | +T | LSJ1 | Non-coding |
| III | 11682310 | g | T | LSJ1 | Non-coding |
| III | 11787364 | c | A | N2 | Non-coding |
| III | 11809760 | t | C | LSJ1 | Non-coding |
| III | 11861727 | a | T | LSJ1 | Non-coding |
| III | 11900751 | g | T | LSJ1 | Non-coding |
| III | 11931828 | t | A | N2 | Non-coding |
| III | 11931842 | a | T | N2 | Non-coding |
| III | 11931876 | g | A | N2 | Non-coding |
| III | 11932089 | t | A | N2 | Non-coding |
| III | 11932103 | a | T | N2 | Non-coding |
| III | 11964563 | g | A | N2 | Non-coding |
| III | 11990788 | * | +T | LSJ1 | Non-coding |
| III | 11993630 | t | A | N2 | Non-coding |
| III | 12008911 | c | G | LSJ1 | Non-coding |
| III | 12021758 | t | A | N2 | Non-coding |
| III | 12059648 | * | +A | LSJ1 | Non-coding |
| III | 12060767 | t | A | LSJ1 | Non-coding |
| III | 12068247 | g | T | N2 | Non-coding |
| III | 12073560 | c | T | N2 | Non-coding |
| III | 12103249 | * | +T |  | Non-coding |
| III | 12131896 | a | T | LSJ1 | Non-coding |
| III | 12131902 | t | A | N2 | Non-coding |
| III | 12160169 | g | T | LSJ1 | Non-coding |
| III | 12239829 | g | A | LSJ1 | Non-coding |
| III | 12376474 | a | G | LSJ1 | Non-coding |
| III | 12449255 | t | A | N2 | Nonsynonymous (*ani-1*) |
| III | 12465763 | g | C | N2 | Non-coding |
| III | 12511724 | g | T | LSJ1 | Non-coding |
| III | 12511902 | a | G | N2 | Non-coding |
| III | 12566565 | * | +AGAAGA | LSJ1 | Non-coding |
| III | 12573266 | g | C | LSJ1 | Non-coding |
| III | 12583128 | a | G | LSJ1 | Non-coding |
| III | 12589044 | t | C | LSJ1 | Non-coding |
| III | 12590593 | c | A | N2 | Non-coding |
| III | 12590609 | g | A | N2 | Non-coding |
| III | 12596783 | t | A | LSJ1 | Non-coding |
| III | 12600755 | c | A | N2 | Non-coding |
| III | 12603551 | g | T | LSJ1 | Non-coding |
| III | 12627210 | g | A | N2 | Non-coding |
| III | 12636081 | t | C | LSJ1 | Non-coding |
| III | 12679353 | * | -TTGG | N2 | Non-coding |
| III | 12683451 | a | C | LSJ1 | Non-coding |
| III | 12705124 | * | +A | LSJ1 | Non-coding |
| III | 12725514 | a | T | N2 | Non-coding |
| III | 12736553 | g | T | N2 | Non-coding |
| III | 12953213 | t | G | LSJ1 | Nonsynonymous (*ttm-1*) |
| III | 13095599 | t | A | unknown | Non-coding |
| III | 13155783 | g | A | LSJ1 | Non-coding |
| III | 13190608 | c | A | N2 | Non-coding |
| III | 13202985 | g | T | LSJ1 | Non-coding |
| III | 13264548 | c | A | LSJ1 | Non-coding |
| III | 13264550 | t | A | LSJ1 | Non-coding |
| III | 13265026 | a | T | LSJ1 | Non-coding |
| III | 13281781 | a | G | LSJ1 | Non-coding |
| III | 13434623 | a | T | N2 | Non-coding |
| III | 13564929 | a | G | LSJ1 | Non-coding |
| III | 13572594 | g | T | N2 | Non-coding |
| III | 13573792 | g | T | N2 | Non-coding |
| III | 13584506 | * | +C | N2 | Non-coding |
| III | 13586011 | * | +G | N2 | Non-coding |
| III | 13598099 | * | -C | N2 | Non-coding |
| III | 13599209 | c | T | N2 | Non-coding |
| III | 13741511 | t | C | LSJ1 | Non-coding |
| III | 13750755 | t | C | N2 | Non-coding |
| IV | 659197 | * | -C | LSJ1 | Non-coding |
| IV | 807401 | a | T | N2 | Non-coding |
| IV | 965102 | g | A | N2 | Non-coding |
| IV | 965103 | t | A | N2 | Non-coding |
| IV | 965114 | g | A | N2 | Non-coding |
| IV | 1142512 | a | T | N2 | Non-coding |
| IV | 1186836 | a | C | N2 | Non-coding |
| IV | 1290721 | t | G | LSJ1 | Non-coding |
| IV | 1474158 | * | +C | N2 | Non-coding |
| IV | 1474748 | * | -GG | N2 | Non-coding |
| IV | 1483377 | t | A | N2 | Non-coding |
| IV | 1487461 | t | C | N2 | Nonsynonymous (Y77E11A.16) |
| IV | 1583914 | t | A | N2 | Non-coding |
| IV | 1643818 | a | T | N2 | Non-coding |
| IV | 1643827 | g | T | LSJ1 | Non-coding |
| IV | 1648098 | g | A | N2 | Non-coding |
| IV | 1648104 | a | T | LSJ1 | Non-coding |
| IV | 1665596 | g | T | LSJ1 | Non-coding |
| IV | 1725515 | c | T | N2 | Non-coding |
| IV | 1750547 | c | A | LSJ1 | Non-coding |
| IV | 1824709 | g | A | N2 | Non-coding |
| IV | 1831614 | a | G | N2 | Non-coding |
| IV | 1989058 | c | A | N2 | Non-coding |
| IV | 2024668 | * | +A | LSJ1 | Non-coding |
| IV | 2025956 | g | A | N2 | Non-coding |
| IV | 2087215 | g | A | N2 | Nonsynonymous (R05C11.3) |
| IV | 2112292 | g | T | LSJ1 | Non-coding |
| IV | 2197543 | * | +A | LSJ1 | Non-coding |
| IV | 2292450 | g | T | N2 | Non-coding |
| IV | 2354169 | * | -T | LSJ1 | Non-coding |
| IV | 2364316 | t | C | N2 | Non-coding |
| IV | 2374749 | * | -C | N2 | Non-coding |
| IV | 2412497 | t | A | N2 | Non-coding |
| IV | 2543741 | * | +G | N2 | Non-coding |
| IV | 2609346 | * | -C | N2 | Non-coding |
| IV | 2625708 | * | -C | N2 | Non-coding |
| IV | 2702423 | t | A | N2 | Non-coding |
| IV | 2702636 | a | G | LSJ1 | Non-coding |
| IV | 2702725 | a | G | N2 | Non-coding |
| IV | 2961079 | * | -C | N2 | Non-coding |
| IV | 3136168 | a | T | N2 | Non-coding |
| IV | 3170029 | a | T | N2 | Nonsynonymous (Y67D8B.1) |
| IV | 3354251 | g | T | N2 | Non-coding |
| IV | 3363333 | g | C | N2 | Non-coding |
| IV | 3376518 | * | -T | N2 | Non-coding |
| IV | 3678794 | * | +C | N2 | Non-coding |
| IV | 3679598 | a | G | N2 | Non-coding |
| IV | 3730706 | g | T | LSJ1 | Non-coding |
| IV | 3842732 | g | A | N2 | Non-coding |
| IV | 3909635 | g | T | N2 | 3' UTR (F56D6.13) |
| IV | 4070824 | * | -C | LSJ1 | Non-coding |
| IV | 4243100 | * | +C | N2 | Non-coding |
| IV | 4303376 | * | -AGTTTTTGA | N2 | Non-coding |
| IV | 4383598 | t | A | N2 | Non-coding |
| IV | 4571781 | a | C | N2 | Non-coding |
| IV | 4612258 | a | T | N2 | Nonsynonymous (F19C7.8) |
| IV | 5011131 | * | +C | N2 | Non-coding |
| IV | 5466144 | a | C | N2 | Non-coding |
| IV | 5598879 | g | T | N2 | Non-coding |
| IV | 6011414 | * | -G | LSJ1 | Non-coding |
| IV | 6434600 | g | T | N2 | Non-coding |
| IV | 6707210 | a | G | N2 | Non-coding |
| IV | 6731479 | * | -T | LSJ1 | Non-coding |
| IV | 6731547 | a | C | LSJ1 | Non-coding |
| IV | 6895403 | c | A | N2 | Non-coding |
| IV | 6922137 | * | +G | N2 | Non-coding |
| IV | 7128301 | * | -GG | LSJ1 | Non-coding |
| IV | 7309840 | a | T | N2 | miRNA target (*grl-4* 3' UTR) |
| IV | 7681505 | g | A | N2 | Non-coding |
| IV | 7681510 | t | A | LSJ1 | Non-coding |
| IV | 7976528 | a | T | N2 | Non-coding |
| IV | 8061838 | c | T | LSJ1 | Non-coding |
| IV | 8374859 | * | +T | LSJ1 | Non-coding |
| IV | 8467243 | t | A | LSJ1 | Non-coding |
| IV | 8468528 | * | -T | LSJ1 | Non-coding |
| IV | 8574211 | * | -T | N2 | Non-coding |
| IV | 8574970 | * | -G | N2 | Non-coding |
| IV | 8575047 | * | -AATAGC | N2 | Non-coding |
| IV | 8578159 | * | +T | N2 | Non-coding |
| IV | 8579713 | t | A | N2 | Non-coding |
| IV | 8581182 | c | T | N2 | Non-coding |
| IV | 8581722 | c | T | N2 | Non-coding |
| IV | 8894794 | a | C | N2 | Non-coding |
| IV | 9059672 | a | G | LSJ1 | Non-coding |
| IV | 9126163 | g | T | N2 | Non-coding |
| IV | 9247339 | t | A | N2 | Non-coding |
| IV | 9400507 | g | T | N2 | Nonsynonymous (F56D5.3) |
| IV | 9433572 | t | C | N2 | Non-coding |
| IV | 10018291 | g | A | N2 | Non-coding |
| IV | 10105711 | * | +AATTTGCCG | N2 | Non-coding |
| IV | 10809631 | g | T | N2 | Non-coding |
| IV | 10945085 | g | C | LSJ1 | Non-coding |
| IV | 10997632 | * | +C | N2 | Non-coding |
| IV | 11075887 | a | C | LSJ1 | Non-coding |
| IV | 11390927 | a | T | N2 | Non-coding |
| IV | 11491330 | c | A | N2 | Non-coding |
| IV | 11522361 | g | A | LSJ1 | Non-coding |
| IV | 11615486 | t | G | LSJ1 | Non-coding |
| IV | 11616772 | t | C | LSJ1 | Non-coding |
| IV | 11616795 | a | C | LSJ1 | Non-coding |
| IV | 12048852 | a | G | LSJ1 | Non-coding |
| IV | 12048854 | a | G | LSJ1 | Non-coding |
| IV | 12098692 | a | T | N2 | Non-coding |
| IV | 12138205 | t | C | LSJ1 | Non-coding |
| IV | 12156146 | t | C | LSJ1 | Non-coding |
| IV | 12323965 | a | C | LSJ1 | Non-coding |
| IV | 12334254 | c | A | LSJ1 | Non-coding |
| IV | 12391460 | * | +T | LSJ1 | Non-coding |
| IV | 12430402 | a | T | LSJ1 | Non-coding |
| IV | 12514477 | t | G | LSJ1 | Non-coding |
| IV | 12544079 | * | +C | LSJ1 | Non-coding |
| IV | 12578261 | c | A | N2 | Nonsynonymous (K08E7.5) |
| IV | 12617975 | * | -A | N2 | Non-coding |
| IV | 12781072 | a | C | LSJ1 | Non-coding |
| IV | 13092702 | g | C | LSJ1 | Non-coding |
| IV | 13285319 | a | T | N2 | Non-coding |
| IV | 13464340 | t | C | LSJ1 | Non-coding |
| IV | 13470854 | * | +A | unknown | Non-coding |
| IV | 13659624 | t | A | LSJ1 | Non-coding |
| IV | 13695677 | c | G | LSJ1 | Non-coding |
| IV | 13823088 | * | +CT | N2 | Non-coding |
| IV | 13832613 | c | A | LSJ1 | Non-coding |
| IV | 13838595 | g | A | unknown | Non-coding |
| IV | 13847607 | a | T | LSJ1 | Non-coding |
| IV | 13899168 | * | -A | unknown | Non-coding |
| IV | 13924407 | c | T | LSJ1 | Non-coding |
| IV | 14004906 | * | -A | LSJ1 | Non-coding |
| IV | 14058349 | c | A | N2 | Non-coding |
| IV | 14339869 | * | +CC | N2 | Non-coding |
| IV | 14429767 | * | +TT | N2 | Non-coding |
| IV | 14469921 | g | T | N2 | Non-coding |
| IV | 14525565 | * | -G | N2 | Non-coding |
| IV | 14534781 | g | C | N2 | Splice Acceptor (T27E7.4) |
| IV | 14707345 | a | T | LSJ1 | Non-coding |
| IV | 14729499 | * | +G | N2 | Non-coding |
| IV | 14877168 | g | T | N2 | Non-coding |
| IV | 14952144 | a | G | N2 | Non-coding |
| IV | 15060614 | a | T | LSJ1 | Non-coding |
| IV | 15078031 | g | A | N2 | Non-coding |
| IV | 15080122 | t | A | N2 | Non-coding |
| IV | 15188007 | a | C | LSJ1 | Non-coding |
| IV | 15188357 | * | +C | N2 | Non-coding |
| IV | 15188946 | t | A | N2 | Non-coding |
| IV | 15306723 | t | C | LSJ1 | Synonymous (Y73F8A.11) |
| IV | 15438158 | t | C | LSJ1 | Non-coding |
| IV | 15476841 | * | +AGA | LSJ1 | Non-coding |
| IV | 15501403 | * | +C | N2 | Non-coding |
| IV | 15512270 | c | T | N2 | Non-coding |
| IV | 15556816 | g | C | LSJ1 | Non-coding |
| IV | 15575202 | * | +A | N2 | exonic (Y105C5A1170) |
| IV | 15710762 | * | +C | LSJ1 | Non-coding |
| IV | 15783550 | * | +G | LSJ1 | Non-coding |
| IV | 15862850 | c | A | LSJ1 | Non-coding |
| IV | 15892490 | g | A | N2 | Synonymous (*srv-14*) |
| IV | 15896173 | t | C | N2 | Non-coding |
| IV | 15921196 | g | T | N2 | Non-coding |
| IV | 16152075 | * | +T | LSJ1 | Non-coding |
| IV | 16330795 | a | T | LSJ1 | Splice Acceptor (*srz-74*) |
| IV | 16360584 | * | +T | N2 | Non-coding |
| IV | 16426593 | * | +AA | N2 | Non-coding |
| IV | 16464101 | t | G | LSJ1 | Non-coding |
| IV | 16491479 | c | A | N2 | Non-coding |
| IV | 16553048 | * | -G | N2 | Non-coding |
| IV | 16556852 | * | +C | N2 | Non-coding |
| IV | 16571390 | * | +C | N2 | Non-coding |
| IV | 16580220 | a | T | N2 | Non-coding |
| IV | 16580738 | a | T | N2 | Non-coding |
| IV | 16580740 | a | T | N2 | Non-coding |
| IV | 16607837 | c | A | LSJ1 | Non-coding |
| IV | 16646674 | g | A | LSJ1 | Non-coding |
| IV | 16646678 | c | G | N2 | Non-coding |
| IV | 16731877 | g | C | LSJ1 | Non-coding |
| IV | 16780504 | c | T | LSJ1 | Synonymous (Y43D4A.5) |
| IV | 16780515 | c | T | LSJ1 | Nonsynonymous (Y43D4A.5) |
| IV | 16780516 | a | C | LSJ1 | Nonsynonymous (Y43D4A.5) |
| IV | 16785103 | c | A | LSJ1 | Non-coding |
| IV | 16785127 | c | T | N2 | Non-coding RNA (Y43D4A.41) |
| IV | 16785129 | t | C | N2 | Non-coding RNA (Y43D4A.41) |
| IV | 16787024 | c | A | N2 | Non-coding |
| IV | 16787035 | c | T | N2 | Non-coding |
| IV | 16787038 | c | A | LSJ1 | Non-coding |
| IV | 16787189 | t | C | N2 | Non-coding |
| IV | 16787192 | g | A | N2 | Non-coding |
| IV | 16787267 | a | G | LSJ1 | Non-coding |
| IV | 16848636 | * | +A | LSJ1 | Non-coding |
| IV | 16848870 | a | C | LSJ1 | Non-coding |
| IV | 16927845 | c | A | N2 | Non-coding |
| IV | 16964877 | * | -G | N2 | Non-coding |
| IV | 17009357 | t | C | N2 | Non-coding |
| IV | 17095482 | c | T | N2 | Non-coding |
| IV | 17208541 | c | T | N2 | Non-coding |
| IV | 17259369 | * | +C | N2 | Non-coding |
| IV | 17260158 | c | T | N2 | Non-coding |
| IV | 17272214 | c | T | N2 | miRNA target (*gln-5* 3' UTR) |
| IV | 17492346 | g | A | LSJ1 | Non-coding |
| IV | 17493788 | * | +C | N2 | Non-coding |
| V | 64713 | g | A | N2 | Non-coding |
| V | 540666 | a | T | N2 | Non-coding |
| V | 941770 | * | -T | LSJ1 | Non-coding |
| V | 955518 | g | T | LSJ1 | Non-coding |
| V | 956804 | t | A | N2 | Non-coding |
| V | 1192202 | g | T | N2 | Non-coding |
| V | 1210996 | a | T | N2 | Non-coding |
| V | 1252440 | * | -G | N2 | Non-coding |
| V | 1578479 | a | T | N2 | Non-coding |
| V | 1644994 | a | C | LSJ1 | Non-coding |
| V | 1644995 | t | C | LSJ1 | Non-coding |
| V | 1880516 | a | T | N2 | Non-coding |
| V | 1880606 | * | +T | unknown | Non-coding |
| V | 2278930 | a | C | N2 | Non-coding |
| V | 2580331 | * | -G | N2 | Non-coding |
| V | 2618353 | t | A | LSJ1 | Nonsynonymous (F53E10.1) |
| V | 2657933 | c | T | LSJ1 | Non-coding |
| V | 2688519 | * | +G | N2 | Non-coding |
| V | 2699940 | t | C | N2 | Non-coding |
| V | 2705734 | * | -A | N2 | Non-coding |
| V | 3186032 | * | +G | N2 | Non-coding |
| V | 3211622 | c | A | N2 | Non-coding |
| V | 3307488 | * | +A | LSJ1 | Non-coding |
| V | 3555631 | * | -G | N2 | Non-coding |
| V | 3568979 | g | T | LSJ1 | Non-coding |
| V | 3697676 | * | -A | N2 | Non-coding |
| V | 4002551 | c | G | N2 | Nonsynonymous (K09D9.12) |
| V | 4580168 | c | G | N2 | Non-coding |
| V | 4592281 | a | C | N2 | Non-coding |
| V | 4592282 | a | T | N2 | Non-coding |
| V | 4661389 | a | T | N2 | Non-coding |
| V | 4764647 | * | +T | LSJ1 | exonic (C18G1.8) |
| V | 5028975 | a | T | N2 | Non-coding |
| V | 5251529 | a | G | N2 | Non-coding |
| V | 5396323 | g | A | N2 | Non-coding |
| V | 5641585 | a | G | LSJ1 | Non-coding |
| V | 5939122 | c | T | N2 | Non-coding |
| V | 6046025 | c | G | LSJ1 | Nonsynonymous (R01B10.4) |
| V | 6091718 | t | G | N2 | Non-coding |
| V | 6094819 | * | +G | N2 | Non-coding |
| V | 6507892 | * | +C | N2 | Non-coding |
| V | 6657071 | t | A | LSJ1 | Non-coding |
| V | 6683996 | g | C | N2 | Non-coding |
| V | 6700463 | * | +GG | LSJ1 | Non-coding |
| V | 6713265 | * | -G | N2 | Non-coding |
| V | 6944418 | a | T | N2 | Non-coding |
| V | 6944457 | g | T | N2 | Non-coding |
| V | 6944463 | a | T | N2 | Non-coding |
| V | 7212742 | a | T | N2 | Non-coding |
| V | 7335549 | * | -A | N2 | Non-coding |
| V | 7392319 | g | A | N2 | Non-coding |
| V | 7609904 | * | +A | LSJ1 | Non-coding |
| V | 7670216 | c | A | N2 | Non-coding |
| V | 7725338 | t | A | LSJ1 | Nonsynonymous (ZK105.8) |
| V | 7725346 | t | C | LSJ1 | Synonymous (ZK105.8) |
| V | 7725387 | c | T | LSJ1 | Synonymous (ZK105.8) |
| V | 7919682 | a | G | N2 | Non-coding |
| V | 8063694 | * | +TCGACGATGCTCATACTCCA | N2 | Non-coding |
| V | 8176186 | a | T | N2 | Non-coding |
| V | 8180777 | a | T | unknown | Non-coding |
| V | 8224823 | * | +C | LSJ1 | Non-coding |
| V | 8485852 | * | -CGAT | N2 | Non-coding |
| V | 9027394 | g | A | N2 | Non-coding |
| V | 9063880 | c | A | N2 | Non-coding |
| V | 9201141 | * | +C | N2 | Non-coding |
| V | 9603824 | g | C | N2 | Non-coding |
| V | 10274848 | * | +C | N2 | Non-coding |
| V | 10617960 | * | +A | N2 | Non-coding |
| V | 10768953 | g | A | LSJ1 | Non-coding |
| V | 10897169 | g | A | N2 | Non-coding |
| V | 10911329 | a | T | N2 | Non-coding |
| V | 11019541 | g | A | N2 | Non-coding |
| V | 11130569 | a | G | LSJ1 | Non-coding |
| V | 11435293 | * | +T | N2 | Non-coding |
| V | 11505099 | t | G | N2 | Non-coding |
| V | 11505100 | g | A | N2 | Non-coding |
| V | 11560038 | g | T | LSJ1 | Non-coding |
| V | 11596660 | c | A | N2 | Non-coding |
| V | 11688121 | t | A | N2 | Non-coding |
| V | 11692431 | c | T | N2 | Non-coding |
| V | 12127735 | a | G | N2 | Non-coding |
| V | 12167423 | c | G | LSJ1 | Synonymous (*col-37*) |
| V | 12375950 | * | +CAG | N2 | Non-coding |
| V | 12484205 | * | +C | N2 | exonic (*atn-1*) |
| V | 12503974 | a | T | N2 | Non-coding |
| V | 12509937 | t | C | N2 | Non-coding |
| V | 12529189 | c | T | N2 | Non-coding |
| V | 12574684 | * | -A | LSJ1 | Non-coding |
| V | 12864064 | a | T | LSJ1 | Non-coding |
| V | 12864465 | g | C | LSJ1 | Non-coding |
| V | 12864663 | g | A | N2 | Non-coding |
| V | 12864665 | a | G | N2 | Non-coding |
| V | 12990999 | t | A | N2 | Non-coding |
| V | 13001654 | * | +C | N2 | Non-coding |
| V | 13206633 | c | T | N2 | Non-coding |
| V | 13307390 | c | A | LSJ1 | Non-coding |
| V | 13646640 | a | T | LSJ1 | Non-coding |
| V | 13857138 | a | T | N2 | Non-coding |
| V | 13926438 | * | +T | N2 | Non-coding |
| V | 14105438 | c | T | N2 | 3' UTR (D1086.9) |
| V | 14358620 | t | G | N2 | Non-coding |
| V | 14556088 | a | T | N2 | Nonsynonymous (*srg-47*) |
| V | 14743838 | t | C | LSJ1 | Non-coding |
| V | 14744049 | * | +C | N2 | Non-coding |
| V | 14744201 | * | +C | N2 | Non-coding |
| V | 14744704 | * | +C | LSJ1 | Non-coding |
| V | 14744852 | t | C | LSJ1 | Non-coding |
| V | 14744882 | a | C | LSJ1 | Non-coding |
| V | 14745023 | c | G | LSJ1 | Non-coding |
| V | 14754697 | t | A | N2 | Non-coding |
| V | 14755152 | t | A | N2 | Non-coding |
| V | 14755222 | t | A | LSJ1 | Non-coding |
| V | 14756478 | a | C | N2 | Non-coding |
| V | 14952220 | t | A | unknown | Non-coding |
| V | 15072901 | * | -CA | N2 | Non-coding |
| V | 15581676 | t | C | LSJ1 | Non-coding |
| V | 15581801 | t | C | LSJ1 | Non-coding |
| V | 15807371 | g | A | N2 | Non-coding |
| V | 15861990 | * | +GGG | LSJ1 | Non-coding |
| V | 16274160 | a | G | LSJ1 | Non-coding |
| V | 16399241 | c | A | N2 | Non-coding |
| V | 16462873 | a | G | N2 | Non-coding |
| V | 16484368 | t | G | LSJ1 | Non-coding |
| V | 16552426 | t | A | N2 | Non-coding |
| V | 16577054 | c | T | LSJ1 | Non-coding |
| V | 16577086 | a | C | LSJ1 | Non-coding |
| V | 16723977 | t | C | LSJ1 | Non-coding |
| V | 16723993 | g | C | LSJ1 | Non-coding |
| V | 16759819 | c | T | N2 | Non-coding |
| V | 16816476 | t | C | LSJ1 | Non-coding |
| V | 16873557 | c | A | N2 | Non-coding |
| V | 17040210 | t | C | LSJ1 | Non-coding |
| V | 17373098 | g | T | N2 | Non-coding |
| V | 17377316 | * | +A | N2 | Non-coding |
| V | 17412207 | c | A | N2 | Non-coding |
| V | 17412211 | t | A | N2 | Non-coding |
| V | 17412226 | c | A | LSJ1 | Non-coding |
| V | 17456937 | g | A | LSJ1 | Nonsynonymous (*str-200*) |
| V | 17619073 | c | G | LSJ1 | Non-coding |
| V | 17738206 | t | C | N2 | Non-coding |
| V | 17738238 | t | C | N2 | Non-coding |
| V | 17867118 | t | G | LSJ1 | Non-coding |
| V | 17867627 | a | T | LSJ1 | Non-coding |
| V | 17898272 | g | A | LSJ1 | Non-coding |
| V | 17920393 | * | +A | N2 | Non-coding |
| V | 17960690 | a | G | LSJ1 | Non-coding |
| V | 17961088 | t | A | LSJ1 | Non-coding |
| V | 17972051 | g | T | N2 | Non-coding |
| V | 17972070 | g | T | LSJ1 | Non-coding |
| V | 17972080 | g | T | LSJ1 | Non-coding |
| V | 18002416 | a | C | N2 | Non-coding |
| V | 18022415 | g | A | unknown | Non-coding |
| V | 18122218 | t | A | N2 | Non-coding |
| V | 18165205 | c | A | LSJ1 | Non-coding |
| V | 18176199 | t | C | LSJ1 | Non-coding |
| V | 18176214 | g | A | LSJ1 | Non-coding |
| V | 18312867 | c | T | N2 | Non-coding |
| V | 18335625 | c | T | N2 | Non-coding |
| V | 18403393 | t | C | LSJ1 | Non-coding |
| V | 18465993 | t | G | LSJ1 | Non-coding |
| V | 18475616 | t | C | LSJ1 | Non-coding |
| V | 18475622 | t | C | N2 | Non-coding |
| V | 18493716 | a | T | N2 | Non-coding |
| V | 18656480 | t | C | LSJ1 | Non-coding |
| V | 18675105 | c | T | N2 | Non-coding |
| V | 18679767 | c | T | LSJ1 | Non-coding |
| V | 18680362 | a | C | LSJ1 | Non-coding |
| V | 18680591 | g | A | N2 | Non-coding |
| V | 18681585 | g | T | N2 | Non-coding |
| V | 18683437 | g | T | N2 | Non-coding |
| V | 18720594 | t | A | N2 | Non-coding |
| V | 18858782 | t | G | N2 | Non-coding |
| V | 18893990 | g | C | N2 | Non-coding |
| V | 18918372 | a | G | N2 | Non-coding |
| V | 18959045 | a | G | N2 | Non-coding |
| V | 18959077 | g | T | N2 | Non-coding |
| V | 18959114 | a | G | LSJ1 | Non-coding |
| V | 18959149 | a | G | LSJ1 | Non-coding |
| V | 18962810 | c | T | LSJ1 | Non-coding |
| V | 18962826 | * | +G | LSJ1 | Non-coding |
| V | 18980126 | t | A | N2 | Non-coding |
| V | 18980199 | a | T | N2 | Non-coding |
| V | 18980208 | c | T | N2 | Non-coding |
| V | 19100192 | a | T | N2 | Non-coding |
| V | 19111416 | a | C | N2 | Non-coding |
| V | 19116041 | * | -G | N2 | Non-coding |
| V | 19178232 | * | +G | LSJ1 | Non-coding |
| V | 19187353 | t | C | LSJ1 | Non-coding |
| V | 19191369 | a | G | N2 | Non-coding |
| V | 19366738 | t | C | N2 | Non-coding |
| V | 19416203 | * | -A | N2 | Non-coding |
| V | 19499424 | g | C | LSJ1 | Non-coding |
| V | 19506793 | t | A | N2 | Non-coding |
| V | 19569701 | t | C | N2 | Non-coding |
| V | 19754362 | * | -TA | N2 | Non-coding |
| V | 19793550 | * | +T | LSJ1 | exonic (M162.7) |
| V | 19800168 | a | G | LSJ1 | Non-coding |
| V | 19806341 | * | +A | N2 | Non-coding |
| V | 19813794 | * | +A | LSJ1 | Non-coding |
| V | 19816962 | c | T | LSJ1 | Non-coding |
| V | 19820264 | * | -GCTTAG | N2 | Non-coding |
| V | 19868214 | c | G | LSJ1 | Non-coding |
| V | 19877707 | * | -G | N2 | Non-coding |
| V | 19905602 | c | T | N2 | Non-coding |
| V | 19905747 | c | T | N2 | Non-coding |
| V | 19905754 | c | T | LSJ1 | Non-coding |
| V | 20002725 | a | G | LSJ1 | Non-coding |
| V | 20019875 | c | A | N2 | Non-coding |
| V | 20226331 | c | T | N2 | Non-coding |
| V | 20328259 | * | -G | LSJ1 | Non-coding |
| V | 20383584 | g | T | N2 | Non-coding |
| V | 20430268 | t | C | N2 | Non-coding |
| V | 20430303 | c | T | N2 | Non-coding |
| V | 20430334 | a | C | N2 | Non-coding |
| V | 20430338 | t | C | N2 | Non-coding |
| V | 20430347 | t | G | N2 | Non-coding |
| V | 20446699 | * | +T | N2 | Non-coding |
| V | 20447867 | * | +A | LSJ1 | Non-coding |
| V | 20464758 | * | -T | LSJ1 | Non-coding |
| V | 20466134 | c | A | LSJ1 | Non-coding |
| V | 20550701 | c | T | LSJ1 | Non-coding |
| V | 20578178 | * | +C | LSJ1 | Non-coding |
| V | 20705205 | t | C | LSJ1 | Non-coding |
| V | 20756343 | c | A | LSJ1 | Non-coding |
| V | 20775918 | * | -A | LSJ1 | Non-coding |
| V | 20787801 | t | A | N2 | Non-coding |
| V | 20787860 | g | C | LSJ1 | Non-coding |
| V | 20787870 | a | G | LSJ1 | Non-coding |
| V | 20787878 | a | C | LSJ1 | Non-coding |
| V | 20924193 | * | +A | N2 | Non-coding |
| X | 59020 | * | +C | N2 | Non-coding |
| X | 106701 | a | G | LSJ1 | Non-coding |
| X | 347028 | a | T | N2 | Non-coding |
| X | 348173 | * | +A | LSJ1 | Non-coding |
| X | 380320 | t | C | N2 | Non-coding |
| X | 466117 | a | T | N2 | Non-coding |
| X | 626844 | c | T | N2 | Non-coding |
| X | 739474 | * | +G | N2 | Non-coding |
| X | 837210 | * | +C | N2 | Non-coding |
| X | 1155872 | * | -T | N2 | Non-coding |
| X | 1219911 | * | -GG | N2 | exonic (C46H3.2) |
| X | 1244284 | c | G | N2 | Non-coding |
| X | 1287082 | * | +C | LSJ1 | Non-coding |
| X | 1417341 | g | T | N2 | Synonymous (F19G12.4) |
| X | 1589004 | t | A | N2 | Non-coding |
| X | 1615117 | * | -C | N2 | exonic (H11E01.3) |
| X | 1725462 | g | T | N2 | Non-coding |
| X | 1769522 | g | A | N2 | Non-coding |
| X | 1884743 | c | A | LSJ1 | Non-coding |
| X | 2004084 | a | T | LSJ1 | Non-coding |
| X | 2004106 | a | T | N2 | Non-coding |
| X | 2004309 | c | A | N2 | Non-coding |
| X | 2008451 | a | T | N2 | Non-coding |
| X | 2008457 | a | G | N2 | Non-coding |
| X | 2008590 | * | -A | LSJ1 | Non-coding |
| X | 2008664 | * | +T | N2 | Non-coding |
| X | 2008675 | a | C | LSJ1 | Non-coding |
| X | 2008779 | g | A | N2 | Non-coding |
| X | 2042489 | * | -G | N2 | Non-coding |
| X | 2058659 | t | C | LSJ1 | Non-coding |
| X | 2058730 | t | A | N2 | Non-coding |
| X | 2234013 | a | T | N2 | Non-coding |
| X | 2234018 | a | T | N2 | Non-coding |
| X | 2318627 | * | +C | N2 | Non-coding |
| X | 2428303 | * | +T | LSJ1 | Non-coding |
| X | 2493072 | t | C | LSJ1 | Non-coding |
| X | 2626109 | c | G | N2 | Non-coding |
| X | 2702795 | * | +C | N2 | Non-coding |
| X | 2798665 | g | A | LSJ1 | Non-coding |
| X | 3080275 | * | +T | N2 | Non-coding |
| X | 3102868 | * | -A | N2 | Non-coding |
| X | 3207459 | * | -C | LSJ1 | Non-coding |
| X | 3306171 | c | A | LSJ1 | Non-coding |
| X | 3306182 | c | A | N2 | Non-coding |
| X | 3441195 | t | A | N2 | Non-coding |
| X | 3479036 | * | +A | N2 | Non-coding |
| X | 3491911 | * | +ATT | LSJ1 | Non-coding |
| X | 3640045 | * | -T | N2 | Non-coding |
| X | 3645065 | g | A | N2 | Non-coding |
| X | 3796657 | t | C | N2 | Non-coding |
| X | 3812010 | g | C | N2 | Non-coding |
| X | 3812011 | t | G | N2 | Non-coding |
| X | 3812324 | g | T | N2 | Synonymous (F35A5.1) |
| X | 3860487 | * | +T | LSJ1 | Non-coding |
| X | 3902127 | * | -GG | N2 | Non-coding |
| X | 4074374 | * | +G | LSJ1 | Non-coding |
| X | 4576631 | * | +T | LSJ1 | Non-coding |
| X | 4684485 | t | G | N2 | Non-coding |
| X | 4730990 | a | G | N2 | Non-coding |
| X | 4740236 | * | +C | N2 | Non-coding |
| X | 4768758 | c | A | LSJ1 | Nonsynonymous (*npr-1*) |
| X | 4907923 | t | A | N2 | Non-coding |
| X | 5004530 | * | +C | N2 | Non-coding |
| X | 5314254 | t | C | LSJ1 | Non-coding |
| X | 5337459 | a | T | N2 | Non-coding |
| X | 5403139 | * | +G | N2 | Non-coding |
| X | 5634223 | c | T | N2 | Non-coding |
| X | 5649053 | g | A | N2 | Non-coding |
| X | 5649066 | g | A | N2 | Non-coding |
| X | 5795787 | c | T | N2 | Non-coding |
| X | 5889914 | * | +CCC | N2 | Non-coding |
| X | 5912417 | * | +T | N2 | Non-coding |
| X | 6080548 | * | +C | N2 | Non-coding |
| X | 6287251 | t | A | N2 | Non-coding |
| X | 6287441 | t | A | N2 | Non-coding |
| X | 6287474 | * | +A | N2 | Non-coding |
| X | 6518290 | * | +CCACCACCCA | N2 | Non-coding |
| X | 6548503 | c | T | N2 | Non-coding |
| X | 6746867 | c | A | N2 | Non-coding |
| X | 6816786 | c | T | N2 | 3' UTR (*atg-2*) |
| X | 6838641 | c | T | N2 | Non-coding |
| X | 6838650 | g | T | N2 | Non-coding |
| X | 7070508 | * | -A | N2 | Non-coding |
| X | 7070541 | * | +C | N2 | Non-coding |
| X | 7112263 | g | T | N2 | Non-coding |
| X | 7164855 | c | T | LSJ1 | Non-coding |
| X | 7362977 | a | G | LSJ1 | Non-coding |
| X | 7524459 | g | T | N2 | Non-coding |
| X | 7764875 | t | C | N2 | Non-coding |
| X | 7768178 | * | +G | N2 | Non-coding |
| X | 7771422 | a | G | N2 | Non-coding |
| X | 7809295 | a | T | N2 | Non-coding |
| X | 8182099 | c | T | N2 | Non-coding |
| X | 8374922 | * | -TCTAAA | N2 | Non-coding |
| X | 8444383 | * | +CC | N2 | Non-coding |
| X | 8509475 | c | T | N2 | Non-coding |
| X | 8602903 | * | -T | LSJ1 | Non-coding |
| X | 8603039 | * | -A | N2 | Non-coding |
| X | 8880147 | g | C | N2 | Non-coding |
| X | 8906072 | a | C | N2 | Non-coding |
| X | 9051616 | * | -G | N2 | Non-coding |
| X | 9188678 | t | A | N2 | Non-coding |
| X | 9189495 | g | A | LSJ1 | Non-coding |
| X | 9260306 | c | T | LSJ1 | Non-coding |
| X | 9260316 | c | A | LSJ1 | Non-coding |
| X | 9343601 | * | +A | N2 | exonic (*oga-1*) |
| X | 9439291 | g | A | N2 | Non-coding |
| X | 9647216 | * | +G | N2 | Non-coding |
| X | 9748888 | c | T | N2 | Non-coding |
| X | 10006361 | c | T | N2 | Non-coding |
| X | 10361070 | a | T | LSJ1 | Non-coding |
| X | 10396717 | * | -C | N2 | Non-coding |
| X | 10590340 | c | T | N2 | Nonsynonymous (*sdn-1*) |
| X | 11311002 | g | A | LSJ1 | Non-coding |
| X | 11311008 | g | A | LSJ1 | Non-coding |
| X | 11484641 | c | T | N2 | Non-coding |
| X | 11773699 | * | -C | LSJ1 | Non-coding |
| X | 11773745 | * | +G | LSJ1 | Non-coding |
| X | 11807898 | g | C | N2 | Non-coding |
| X | 11886519 | * | +C | N2 | Non-coding |
| X | 11978591 | * | -G | N2 | Non-coding |
| X | 12093630 | t | A | N2 | Non-coding |
| X | 12276466 | t | A | LSJ1 | Non-coding |
| X | 12276610 | t | A | LSJ1 | Non-coding |
| X | 12277051 | t | C | N2 | Non-coding |
| X | 12277056 | g | A | N2 | Non-coding |
| X | 12277064 | a | K | N2 | Non-coding |
| X | 12277183 | c | T | LSJ1 | Non-coding |
| X | 12278271 | * | -C | LSJ1 | Non-coding |
| X | 12352259 | c | T | N2 | Non-coding |
| X | 12352273 | g | T | LSJ1 | Non-coding |
| X | 12419528 | a | T | LSJ1 | Non-coding |
| X | 12578042 | c | A | N2 | Non-coding |
| X | 12689723 | g | A | N2 | Non-coding |
| X | 12812572 | t | G | N2 | Non-coding |
| X | 12828874 | * | -TAG | N2 | Non-coding |
| X | 12842448 | a | G | N2 | Non-coding |
| X | 12842497 | * | -T | N2 | Non-coding |
| X | 12919746 | * | +CC | N2 | exonic (*pqn-18*) |
| X | 13001478 | c | A | N2 | Non-coding |
| X | 13319522 | t | A | N2 | Non-coding |
| X | 13384452 | g | A | N2 | Non-coding |
| X | 13458356 | g | T | N2 | Non-coding |
| X | 13494282 | t | A | N2 | Non-coding |
| X | 13649405 | * | +C | LSJ1 | Non-coding |
| X | 14229654 | * | -G | N2 | Non-coding |
| X | 14321391 | * | +C | N2 | Non-coding |
| X | 14343414 | g | A | LSJ1 | Non-coding |
| X | 14370155 | g | A | LSJ1 | Non-coding |
| X | 14506147 | * | +C | N2 | Non-coding |
| X | 14568178 | g | T | N2 | Non-coding |
| X | 14664168 | * | -C | LSJ1 | Non-coding |
| X | 14673880 | * | +CA | LSJ1 | Non-coding |
| X | 14844790 | * | -T | unknown | Non-coding |
| X | 14899241 | g | T | LSJ1 | Non-coding |
| X | 14936965 | g | A | N2 | Non-coding |
| X | 14955977 | * | +G | LSJ1 | Non-coding |
| X | 15316753 | g | T | LSJ1 | Non-coding |
| X | 15326698 | c | T | N2 | Non-coding |
| X | 15540505 | * | -C | N2 | Non-coding |
| X | 15643458 | c | A | LSJ1 | Non-coding |
| X | 15748037 | g | A | N2 | Non-coding |
| X | 15750323 | t | C | LSJ1 | Non-coding |
| X | 15750324 | g | C | LSJ1 | Non-coding |
| X | 15785551 | * | +G | N2 | Non-coding |
| X | 15842103 | * | +C | N2 | exonic (F59F4.2) |
| X | 15854245 | t | C | LSJ1 | Non-coding |
| X | 15854254 | g | A | LSJ1 | Non-coding |
| X | 15892251 | * | -GGG | N2 | Non-coding |
| X | 15977125 | * | -G | N2 | Non-coding |
| X | 15987672 | t | A | N2 | Non-coding |
| X | 16170184 | c | T | N2 | Non-coding |
| X | 16183520 | c | A | LSJ1 | Non-coding |
| X | 16224807 | * | -G | N2 | Non-coding |
| X | 16275777 | g | T | LSJ1 | Non-coding |
| X | 16275798 | a | T | N2 | Non-coding |
| X | 16305596 | a | T | LSJ1 | Non-coding |
| X | 16305598 | t | G | LSJ1 | Non-coding |
| X | 16531999 | * | +C | N2 | Non-coding |
| X | 16647104 | t | G | LSJ1 | Non-coding |
| X | 16740021 | t | A | N2 | Non-coding |
| X | 16899468 | g | A | LSJ1 | Non-coding |
| X | 16933742 | c | G | N2 | Non-coding |
| X | 16934838 | * | +C | N2 | Non-coding |
| X | 16960745 | g | T | N2 | Non-coding |
| X | 17294860 | g | T | N2 | Non-coding |
| X | 17294866 | c | T | N2 | Non-coding |
| X | 17319592 | * | +C | N2 | Non-coding |
| X | 17358162 | * | +C | LSJ1 | Non-coding |
| X | 17369604 | * | +G | LSJ1 | Non-coding |
| X | 17441958 | * | -T | N2 | Non-coding |
| X | 17491911 | g | T | LSJ1 | Non-coding |
| X | 17620028 | g | T | N2 | Non-coding |
| X | 17707521 | c | G | N2 | Non-coding |
