# Supplementary material for: Whole Genome Sequencing Highlights Genetic Changes Associated with Laboratory Domestication of C. elegans
Source: PLoS One. 2010 Nov 11;5(11):e13922. doi: 10.1371/journal.pone.0013922 (PMC2978686; doi:10.1371/journal.pone.0013922)
Supplement: Table S3 — Larger insertions and deletions found between N2 and LSJ1: size indicates number of bases inserted or deleted. Deletions include the starting coordinate, and insertions occur after this coordinate. (0.03 MB DOC) [file pone.0013922.s006.doc]

| Chromosome | Start | Size | Gene | Protein Function |
| --- | --- | --- | --- | --- |
| *INSERTIONS* |  |  |  |  |
| 2 | 3568799 | 34 | *clec-127* | c-type lectin |
| 5 | 3587027 | 8 | T22F3.2 | ubiquitin carboxyl-terminal hydrolase |
| 5 | 15432407 | 4 | T26H5.8 | unknown |
| *DELETIONS* |  |  |  |  |
| 1 | 8881328 | 281 | F55H12.3 | EGF-like, calcium-binding |
| 2 | 595619 | 9 | *nhl-3* | E3 ubiquitin ligase |
| x | 8941342 | 5 | *trk-1* | receptor tyrosine kinase |
