# Supplementary material for: Whole Genome Sequencing Highlights Genetic Changes Associated with Laboratory Domestication of C. elegans
Source: PLoS One. 2010 Nov 11;5(11):e13922. doi: 10.1371/journal.pone.0013922 (PMC2978686; doi:10.1371/journal.pone.0013922)
Supplement: Table S4 — Predicted reference errors that alter genome annotation: subset of N2 reference sequence errors that are predicted to affect protein coding predictions. (0.31 MB DOC) [file pone.0013922.s007.doc]

| Chromosome | Coordinate | Reference Allele | Actual Allele | Gene | Amino Acid Change |
| --- | --- | --- | --- | --- | --- |
| I | 221369 | * | +G | Y48G1BL.6 | N/A |
| I | 232037 | * | -G | Y48G1BM.6 | N/A |
| I | 742848 | * | +C | *col-46* | N/A |
| I | 1074824 | * | +C | *gsa-1* | N/A |
| I | 1142103 | c | A | Y48G8AL.10 | Gly/Ser |
| I | 2379643 | c | T | Y39G10AR.17 | Met/Ile |
| I | 2710697 | t | C | Y71F9B.13 | N/A |
| I | 4184637 | * | -A | *ZK39.9* | N/A |
| I | 5003390 | * | +C | *dom-3* | N/A |
| I | 5042624 | * | -GGT | C46H11.7 | N/A |
| I | 5301101 | c | A | *let-526* | N/A |
| I | 6801828 | * | +CT | E02D9.1 | N/A |
| I | 7537821 | * | +C | *dcp-66* | N/A |
| I | 9699160 | t | C | C04F12.8 | Asn/Thr |
| I | 10113278 | * | -G | R12E2.1 | N/A |
| I | 10188886 | t | C | *dao-5* | Lys/Asn |
| I | 11145687 | * | +G | C18H9.6 | N/A |
| I | 11764538 | * | +C | *vab-10* | N/A |
| I | 13101698 | * | -A | *rgs-4* | N/A |
| I | 13168446 | c | G | F22G12.5 | His/Asp |
| I | 13491121 | * | +T | Y48G10A.1 | N/A |
| I | 13617730 | * | +T | Y106G6D.3 | N/A |
| I | 13985036 | a | G | Y71A12B.17 | Lys/Glu |
| II | 213638 | * | +G | F48A11.4 | N/A |
| II | 1577786 | * | +G | C17F4.3 | N/A |
| II | 1848045 | * | +C | *fbxb-97* | N/A |
| II | 1926839 | * | +C | F52C6.2 | N/A |
| II | 2678738 | * | +A | F22E5.17 | N/A |
| II | 2796451 | * | +G | F49C5.11 | N/A |
| II | 2879045 | * | +G | Y110A2AL.12 | N/A |
| II | 2925946 | * | +C | ZK355.2 | N/A |
| II | 3241680 | * | -A | VM106R.1 | N/A |
| II | 3521235 | * | +C | *col-72* | N/A |
| II | 4754157 | * | -G | C27D9.1 | N/A |
| II | 4763294 | * | -A | *ehs-1* | N/A |
| II | 5006737 | * | +G | F59A6.4 | N/A |
| II | 5079263 | * | +C | *dsh-2* | N/A |
| II | 5219639 | * | +G | F41G3.10 | N/A |
| II | 5271459 | * | +G | *drn-1* | N/A |
| II | 5647429 | c | T | *ddl-1* | Lys/Asn |
| II | 5667682 | * | +G | C25H3.7 | N/A |
| II | 5670253 | * | +C | C25H3.8 | N/A |
| II | 5814272 | * | +G | ZK1248.9 | N/A |
| II | 5820840 | * | -G | *ehs-1* | N/A |
| II | 5974545 | * | +T | B0034.5 | N/A |
| II | 5998649 | * | +C | *ins-5* | N/A |
| II | 6193649 | * | +G | *cpb-2* | N/A |
| II | 6200178 | * | +G | tag-319 | N/A |
| II | 6500691 | * | +G | R05G9.3 | N/A |
| II | 6559522 | * | +C | F18C5.10 | N/A |
| II | 6700726 | * | +G | *math-38* | N/A |
| II | 6736304 | c | G | T14B4.2 | N/A |
| II | 6736306 | t | C | T14B4.2 | N/A |
| II | 6749216 | * | -G | F26G1.2 | N/A |
| II | 6750712 | * | +G | F41G3.20 | N/A |
| II | 6759907 | * | +T | F41G3.2 | N/A |
| II | 7003347 | t | A | *unc-104* | Val/Glu |
| II | 7479112 | * | +G | *ubc-6* | N/A |
| II | 7642076 | * | +CT | *pde-4* | N/A |
| II | 7739392 | * | -C | *tag-308* | N/A |
| II | 7754506 | * | -C | B0228.6 | N/A |
| II | 7866905 | * | +A | *ztf-17* | N/A |
| II | 7871211 | * | -G | T01H3.3 | N/A |
| II | 8227927 | g | C | *dab-1* | Arg/Pro |
| II | 8227928 | c | G | *dab-1* | Arg/Pro |
| II | 8584093 | c | T | F49C9.11 | Gly/Val |
| II | 8765136 | * | +G | *pah-1* | N/A |
| II | 9234922 | * | -C | T26C5.2 | N/A |
| II | 10829479 | * | +A | Y38E10A.8 | N/A |
| II | 11207621 | * | +A | T06D8.1 | N/A |
| II | 11384797 | * | +G | *shn-1* | N/A |
| II | 12556600 | * | +G | *dsh-1* | N/A |
| II | 12607617 | * | +A | *rgs-4* | N/A |
| II | 12668767 | * | +G | T06D8.1 | N/A |
| II | 12677300 | * | +G | Y38E10A.23 | N/A |
| II | 12677412 | t | G | Y38E10A.23 | Asn/Ser |
| II | 13090005 | t | A | *gcn-2* | Asp/Glu |
| II | 13440583 | g | T | *tbc-15* | Thr/Met |
| II | 13835091 | * | +GCC | *ins-37* | N/A |
| II | 14796259 | g | T | *eif-3.B* | Gly/Val |
| III | 661016 | * | +G | C09F5.1 | N/A |
| III | 691185 | c | T | W02B3.7 | Glu/Asp |
| III | 835904 | * | +C | *dos-3* | N/A |
| III | 847237 | * | +GGC | K02F3.2 | N/A |
| III | 3366747 | * | +G | *prk-2* | N/A |
| III | 3467137 | t | C | *pph-6* | N/A |
| III | 3467466 | c | T | *pph-6* | N/A |
| III | 3664405 | * | +G | C46F11.4 | N/A |
| III | 4059720 | * | -T | *his-70* | N/A |
| III | 4340348 | * | +T | B0285.1 | N/A |
| III | 4628542 | * | +G | *clec-151* | N/A |
| III | 4640495 | * | +G | *clec-154* | N/A |
| III | 4986886 | * | +GC | C27F2.10 | N/A |
| III | 5105938 | * | +G | F54D8.6 | N/A |
| III | 5333259 | * | +C | F52C9.1 | N/A |
| III | 5467690 | * | -A | F48E8.2 | N/A |
| III | 5595877 | * | +G | *ced-6* | N/A |
| III | 6033357 | * | -A | ZK328.7 | N/A |
| III | 6322253 | * | -C | C56G2.5 | N/A |
| III | 7075892 | * | +C | ZK418.6 | N/A |
| III | 7357294 | * | +C | F08F8.10 | N/A |
| III | 7608528 | * | +C | T04A6.3 | N/A |
| III | 8042706 | * | +G | K12H4.6 | N/A |
| III | 8068403 | * | +G | *ceh-26* | N/A |
| III | 8130858 | * | +C | C14B9.10 | N/A |
| III | 8438265 | * | +C | *pqn-96* | N/A |
| III | 8552915 | * | +G | *gsto-2* | N/A |
| III | 8570399 | * | +C | CE7X_3.1 | N/A |
| III | 8650752 | * | +G | *flp-23* | N/A |
| III | 8706589 | * | +AC | B0303.11 | N/A |
| III | 8976440 | * | -T | *pde-2* | N/A |
| III | 9101167 | g | T | ZK507.1 | Gln/STOP |
| III | 9785903 | * | -T | R10E11.5 | N/A |
| III | 10030883 | * | +G | M04D8.4 | N/A |
| III | 10407463 | * | +G | *tag-235* | N/A |
| III | 10407508 | * | +C | *tag-235* | N/A |
| III | 10483638 | * | -C | D2045.8 | N/A |
| III | 11326567 | * | +T | T28D6.6 | N/A |
| IV | 757658 | t | A | *srt-23* | Phe/Tyr |
| IV | 1515189 | * | +G | K03H6.2 | N/A |
| IV | 3382092 | * | -T | B0546.3 | N/A |
| IV | 4931163 | a | C | Y9C9A.8 | Asn/His |
| IV | 4972666 | c | T | *srz-28* | Val/Phe |
| IV | 5208043 | * | +T | Y59E9AL.4 | N/A |
| IV | 5405344 | * | +T | ZK616.1 | N/A |
| IV | 5409611 | * | +G | ZK616.8 | N/A |
| IV | 7600485 | a | T | *tag-80* | Gln/His |
| IV | 7727542 | * | +G | C50F7.5 | N/A |
| IV | 8271544 | * | -T | K07H8.5 | N/A |
| IV | 8632715 | * | +A | *dgk-4* | N/A |
| IV | 9745669 | g | A | C28D4.5 | His/Asn |
| IV | 9840851 | * | +C | *kin-24* | N/A |
| IV | 10414829 | * | +G | F13B12.6 | N/A |
| IV | 10793215 | g | A | *opt-1* | Glu/Lys |
| IV | 10878679 | g | A | *sdz-13* | Arg/Ser |
| IV | 10894240 | * | +C | F13E9.8 | N/A |
| IV | 11050307 | * | -A | F36H1.3 | N/A |
| IV | 11323985 | * | +G | *his-47* | N/A |
| IV | 13790999 | * | -C | Y45F10D.7 | N/A |
| IV | 15965556 | * | +G | Y105C5B.18 | N/A |
| IV | 17228761 | * | +C | Y116A8B.5 | N/A |
| V | 3750651 | * | +T | Y39H10A.6 | N/A |
| V | 4164869 | a | C | Y45G5AM.3 | Ile/Leu |
| V | 6889637 | g | C | *unc-70* | Ala/Pro |
| V | 6956711 | t | C | *mec-1* | Leu/Pro |
| V | 6956743 | c | G | *mec-1* | Arg/Gly |
| V | 6956744 | g | C | *mec-1* | Arg/Gly |
| V | 7205625 | * | -G | C13F10.4 | N/A |
| V | 7546600 | g | A | *ftn-1* | His/Asn |
| V | 8792708 | * | +A | *tag-117* | N/A |
| V | 9162494 | t | G | *col-145* | Gln/Arg |
| V | 9376379 | g | T | E02C12.10 | Gln/STOP |
| V | 9928614 | c | T | T07C12.12 | Gln/His |
| V | 10548751 | * | -G | *srd-34* | N/A |
| V | 11495158 | * | -T | T11F9.12 | N/A |
| V | 12810474 | * | +T | ZC443.2 | N/A |
| V | 13150205 | * | -G | B0365.7 | N/A |
| V | 13506613 | * | +T | R11G10.4 | N/A |
| V | 13665872 | * | +A | F58G11.2 | N/A |
| X | 1202975 | * | +G | T13G4.6 | N/A |
| X | 2165878 | * | +G | F48B9.3 | N/A |
| X | 2691726 | * | -C | T14G11.1 | N/A |
| X | 3237694 | a | G | F40F4.6 | Phe/Cys |
| X | 3413856 | * | +G | *dhs-27* | N/A |
| X | 4025157 | * | +T | *acr-10* | N/A |
| X | 4565291 | * | +C | *amt-4* | N/A |
| X | 4603453 | * | -G | *spat-3* | N/A |
| X | 4662792 | * | -A | F16H11.2 | N/A |
| X | 4662891 | g | A | *nurf-1* | Glu/Lys |
| X | 5462745 | * | +G | *ddr-1* | N/A |
| X | 5526942 | * | +C | *lgx-1* | N/A |
| X | 5590454 | * | +G | *mec-2* | N/A |
| X | 6075482 | * | +G | *ggr-2* | N/A |
| X | 6283375 | * | +G | T07H6.4 | N/A |
| X | 6374837 | * | +C | C03B1.1 | N/A |
| X | 6792031 | g | C | *asp-3* | Glu/Asp |
| X | 7139443 | * | -C | *ist-1* | N/A |
| X | 7145338 | * | +C | *lam-2* | N/A |
| X | 7436793 | * | +T | C01C10.2 | N/A |
| X | 7588883 | t | C | *adt-2* | Ser/Pro |
| X | 7986170 | * | +G | F45E1.4 | N/A |
| X | 8026787 | * | +T | C34D10.2 | N/A |
| X | 8033807 | * | +C | C18A11.6 | N/A |
| X | 8325357 | * | -G | R09F10.8 | N/A |
| X | 8596016 | * | +G | F18E9.7 | N/A |
| X | 8596061 | * | -G | F18E9.7 | N/A |
| X | 8601955 | * | +C | F18E9.1 | N/A |
| X | 8639857 | * | -G | *stg-2* | N/A |
| X | 9235230 | * | +C | *lgc-4* | N/A |
| X | 9861507 | g | T | R07B1.3 | Ser/Ile |
| X | 11423704 | * | +C | *hum-4* | N/A |
| X | 11624629 | t | C | Y79H9A.1 | Asp/Ala |
| X | 12701752 | * | -G | *nhr-214* | N/A |
| X | 12786146 | g | C | *alh-13* | Trp/Cys |
| X | 13404237 | * | +A | F02C12.1 | N/A |
| X | 14595714 | * | -A | C44H4.8 | N/A |
| X | 14614978 | * | +C | *rbc-1* | N/A |
| X | 14634336 | * | +G | F54E4.3 | N/A |
| X | 14766614 | * | -G | Y16B4A.2 | N/A |
| X | 15226797 | g | T | H03A11.2 | Pro/Leu |
| X | 16817032 | t | C | *sto-5* | Glu/STOP |
| X | 17047302 | c | T | *gcy-11* | Glu/STOP |
| X | 17047309 | t | C | *gcy-11* | Glu/Asp |
| X | 17047323 | g | T | *gcy-11* | Glu/STOP |
| X | 17228978 | * | +G | T25G12.6 | N/A |
| X | 17714409 | * | +C | 6R55.2 | N/A |
|  |  |  |  |  |  |
