# Supplementary material for: Whole Genome Sequencing Highlights Genetic Changes Associated with Laboratory Domestication of C. elegans
Source: PLoS One. 2010 Nov 11;5(11):e13922. doi: 10.1371/journal.pone.0013922 (PMC2978686; doi:10.1371/journal.pone.0013922)
Supplement: Table S5 — N2/LSJ1 protein coding changes: SNPs and small indels between N2 and LSJ1 that are predicted to affect protein coding. (0.15 MB DOC) [file pone.0013922.s008.doc]

| Chromosome | Coordinate | N2 allele | LSJ1 allele | Ancestral | Gene | Gene Function | Amino Acid Change |
| --- | --- | --- | --- | --- | --- | --- | --- |
| I | 229535 | g | T | N2 | Y48G1BM.6 | tyrosine kinase | Thr/Asn |
| I | 238077 | t | G | N2 | Y48G1BM.6 | tyrosine kinase | Lys/Thr |
| I | 5351199 | t | C | LSJ1 | F55A12.8 | P-loop ATPase | Ile/Met |
| I | 8881330 | * | +A | N2 | F55H12.3 | EGF-like | N/A |
| I | 10267627 | * | +T | N2 | ZC247.1 | unknown | N/A |
| I | 13089055 | c | T | N2 | Y26D4A.9 | DNA polymerase | Glu/Lys |
| II | 73323 | c | A | N2 | C23H3.9 | unknown | Leu/Phe |
| II | 207834 | g | T | N2 | *chs-2* | chitin synthase | Val/Phe |
| II | 4368150 | * | +C | LSJ1 | *lat-2* | latrophilin receptor | N/A |
| II | 4821506 | a | G | LSJ1 | *lact-5* | beta-lactamase domain | Leu/Ser |
| II | 5601337 | a | C | LSJ1 | C17G10.6 | ShK domain | Tyr/Asp |
| II | 6084903 | * | +G | LSJ1 | *ptc-2* | sterol-sensing domain | N/A |
| II | 6136222 | t | C | N2 | *abts-3* | anion transporter | Leu/Pro |
| II | 7123986 | * | +G | N2 | F21H12.4 | sterol-sensing domain | N/A |
| II | 7642036 | * | +G | LSJ1 | *pde-4* | phosphodiesterase | N/A |
| II | 7698878 | * | +G | LSJ1 | B0495.6 | unknown | N/A |
| II | 8746874 | * | -C | N2 | K08F8.1 | MAP kinase-activated protein kinase 2 | N/A |
| II | 9863937 | c | T | LSJ1 | C08H9.4 | glycoside hydrolase | Ala/Thr |
| II | 9863960 | a | G | LSJ1 | C08H9.4 | glycoside hydrolase | Asn/Asp |
| II | 12730042 | c | T | N2 | Y46G5A.4 | RNA helicase BRR2, DEAD-box superfamily | Ala/Thr |
| II | 14407665 | * | +CGA | N2 | F26H11.2 | nucleosome remodeling factor | N/A |
| III | 336186 | a | C | N2 | Y50D7A.8 | unknown | Ser/Ala |
| III | 502009 | c | T | N2 | *unc-45* | myosin assembly/sexual cycle | Thr/Met |
| III | 6371226 | a | G | LSJ1 | *thoc-2* | transcription factor/nuclear export subunit | Asp/Gly |
| III | 6619218 | c | T | LSJ1 | Y40D12A.1 | FtsJ-like RNA methyltransferase | Leu/Phe |
| III | 7028660 | c | T | LSJ1 | *cyk-1* | cytokinesis defect | Glu/Lys |
| III | 7163701 | a | C | LSJ1 | K04G7.1 | unknown | Lys/Asn |
| III | 7214106 | a | C | LSJ1 | R151.2 | Phosphoribosyltransferase | Ser/Arg |
| III | 7325087 | g | T | LSJ1 | F56C9.11 | unknown | Pro/His |
| III | 8693609 | g | C | LSJ1 | B0303.7 | contains SH3 domain | Cys/Ser |
| III | 9101168 | c | T | LSJ1 | ZK507.1 | Casein kinase | Met/Ile |
| III | 9259899 | a | G | N2 | M01A8.1 | unknown | Asn/Asp |
| III | 9341371 | t | C | LSJ1 | *emb-9* | basement membrane collagen subunit | Arg/Gly |
| III | 9350630 | c | T | LSJ1 | K04H4.2 | chitin-binding | Gly/Glu |
| III | 9350655 | c | T | LSJ1 | K04H4.2 | chitin-binding | Glu/Lys |
| III | 9754432 | c | A | LSJ1 | *vps-53* | related to yeast vacuolar protein sorting factor | Glu/Asp |
| III | 9785416 | * | -A | N2 | R10E11.6 | AP1 subunit gamma-binding protein 1 | N/A |
| III | 11503256 | t | G | N2 | Y66D12A.21 | GTP-binding | Leu/Phe |
| III | 11503425 | t | C | N2 | Y66D12A.21 | GTP-binding | Lys/Arg |
| III | 11503446 | t | C | N2 | Y66D12A.21 | GTP-binding | Lys/Arg |
| III | 11576715 | c | G | LSJ1 | Y66D12A.8 | unknown | Ala/Gly |
| III | 12449255 | t | A | N2 | *ani-1* | anillin | Glu/Val |
| III | 12953213 | t | G | LSJ1 | *ttm-1* | zinc transporter | Asn/Thr |
| IV | 1487461 | t | C | N2 | Y77E11A.16 | membrane protein | Trp/Arg |
| IV | 2087215 | g | A | N2 | *mca-2* | calcium-transporting ATPase | Arg/Gln |
| IV | 3170029 | a | T | N2 | Y67D8B.1 | GTP-binding | Asp/Glu |
| IV | 4612258 | a | T | N2 | F19C7.8 | unknown | Ile/Asn |
| IV | 9400507 | g | T | N2 | F56D5.3 | NADH:flavin oxidoreductase | Leu/Phe |
| IV | 12578261 | c | A | N2 | K08E7.5 | unknown | Pro/Gln |
| IV | 14534781 | g | C | N2 | T27E7.4 | membrane protein | N/A |
| IV | 15575202 | * | +A | N2 | Y105C5A1170 | unknown | N/A |
| IV | 16330795 | a | T | LSJ1 | *srz-74* | serpentine receptor, class Z | Gln/Leu |
| IV | 16780515 | c | T | LSJ1 | Y43D4A.5 | unknown | Ser/Leu |
| IV | 16780516 | a | C | LSJ1 | Y43D4A.5 | unknown | Ser/Phe |
| IV | 16785127 | c | T | N2 | Y43D4A.41 | 21U-RNA | N/A |
| IV | 16785129 | t | C | N2 | Y43D4A.41 | 21U-RNA | N/A |
| V | 2618353 | t | A | LSJ1 | F53E10.1 | Fe-S protein | Ser/Arg |
| V | 4002551 | c | G | N2 | K09D9.12 | unknown | Glu/Gln |
| V | 4764647 | * | +T | LSJ1 | C18G1.8 | unknown | N/A |
| V | 6046025 | c | G | LSJ1 | R01B10.4 | membrane protein | Val/Leu |
| V | 7725338 | t | A | LSJ1 | ZK105.8 | 7-transmembrane receptor | Phe/Tyr |
| V | 12484205 | * | +C | N2 | *atn-1* | alpha-actinin | N/A |
| V | 14556088 | a | T | N2 | *srg-47* | serpentine receptor, class G | Leu/Gln |
| V | 17456937 | g | A | LSJ1 | *str-200* | 7-transmembrane olfactory receptor | Ser/Leu |
| V | 19793550 | * | +T | LSJ1 | M162.7 | unknown | N/A |
| X | 1219911 | * | -GG | N2 | C46H3.2 | SAM domain | N/A |
| X | 1615117 | * | -C | N2 | H11E01.3 | unknown | N/A |
| X | 4768758 | c | A | LSJ1 | npr-1 | neuropeptide receptor | Val/Phe |
| X | 9343601 | * | +A | N2 | oga-1 | O-GlcNAc-selective N-acetyl-beta-D-glucosaminidase | N/A |
| X | 10590340 | c | T | N2 | sdn-1 | transmembrane heparan sulfate proteoglycan | Arg/Lys |
| X | 12919746 | * | +CC | N2 | pqn-18 | prion-like | N/A |
| X | 15842103 | * | +C | N2 | F59F4.2 | membrane protein | N/A |
